# Enantioselective carbohydrate recognition by synthetic lectins in water†
†Electronic supplementary information (ESI) available: Experimental details for synthesis, characterisation and binding experiments; NMR spectra and binding analyses. See DOI: 10.1039/c6sc05399h



**DOI:** 10.1039/c6sc05399h

**Published:** 2017-03-30

**Authors:** Pablo Ríos, Tiddo J. Mooibroek, Tom S. Carter, Christopher Williams, Miriam R. Wilson, Matthew P. Crump, Anthony P. Davis

**Affiliations:** a School of Chemistry , University of Bristol , Cantock's Close , Bristol BS8 1TS , UK . Email: t.j.mooibroek@uva.nl ; Email: Anthony.Davis@bristol.ac.uk

## Abstract

Carbohydrate receptors with a chiral framework have been generated by combining a tetra-aminopyrene and a *C*_3_-symmetrical triamine *via* isophthalamide spacers bearing water-solubilising groups. These “synthetic lectins” are the first to show enantiodiscrimination in aqueous solution, binding *N*-acetylglucosamine (GlcNAc) with 16 : 1 enantioselectivity. They also show exceptional affinities. GlcNAc is bound with *K*_a_ up to 1280 M^–1^, more than twice that measured for previous synthetic lectins, and three times the value for wheat germ agglutinin, the lectin traditionally employed to bind GlcNAc in glycobiological research. Glucose is bound with *K*_a_ = 250 M^–1^, again higher than previous synthetic lectins. The results suggest that chirality can improve complementarity to carbohydrate substrates and may thus be advantageous in synthetic lectin design.

## Abstract 2

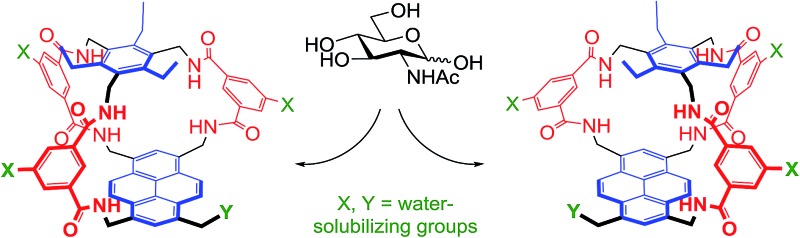
These chiral “synthetic lectins” are the first to discriminate between carbohydrate enantiomers, and also show unprecedented affinities for monosaccharide substrates.

## Introduction

Selective carbohydrate recognition is a long-standing interest of supramolecular chemistry.^1^ The problem has clear relevance for biology, where carbohydrate-binding proteins (lectins) mediate a wide range of processes^2^ including fertilization,^2c–g^ neuronal development,^2d–h^ hormonal activities,^3^ tumour metastasis,^4^ immune surveillance^5^ and inflammatory responses.^6^ At the same time, carbohydrates are exceptionally challenging targets, especially in the natural medium of water.^1*b*^ Typically they are coated with hydroxyl groups and are therefore highly hydrophilic. They are also “hydromimetic” in their resemblance to clusters of water molecules, and are therefore especially difficult to distinguish from competing solvent. Indeed, the affinities of natural lectins for their substrates are notoriously weak on the general scale of biomolecular interactions.^7^ If the challenge can be met, “synthetic lectins” have various potential applications. For example, they may serve as tools for biological research, as diagnostic and therapeutic agents,^1c,8^ and also as models for probing the basis of natural carbohydrate recognition.^9^

A characteristic feature of carbohydrates is their chirality. Indeed, they are often used to illustrate biological asymmetry, and figure strongly in the history of stereochemistry.^10^ Moreover, while natural sugars normally occur as single, specific enantiomers (in most cases *D*), the alternative (*L*) enantiomers are found in some circumstances.^11^ Accordingly, the enantioselective recognition of carbohydrates has attracted much interest.^12,13^ Success has been achieved for organic molecular receptors in organic solvents,^12^ and also for boron-based systems in water.^13^ However, to date there have been no reports of enantioselective recognition by “synthetic lectins”, *i.e.* receptors operating in water through non-covalent interactions.

In previous work, we have developed a number of synthetic lectins which target the all-equatorial family of carbohydrates, *i.e.* glucose, *N*-acetylglucosamine (GlcNAc) and derivatives.^1b,14^ These substrates are important for various reasons. For example glucose is a major analyte in medicine and biotechnology.^15^ Selective glucose receptors have potential as components of glucose monitors, which could be used to aid the management of diabetes and also to follow fermentation and cell growth. Meanwhile GlcNAc, β-linked to serine and threonine, is a dynamic post-translational modification of proteins which is currently under intensive investigation.^16^ Selective GlNAc receptors could be used in stains for modified proteins, and in other tools for β-GlcNAc research.

**Fig. 1:**
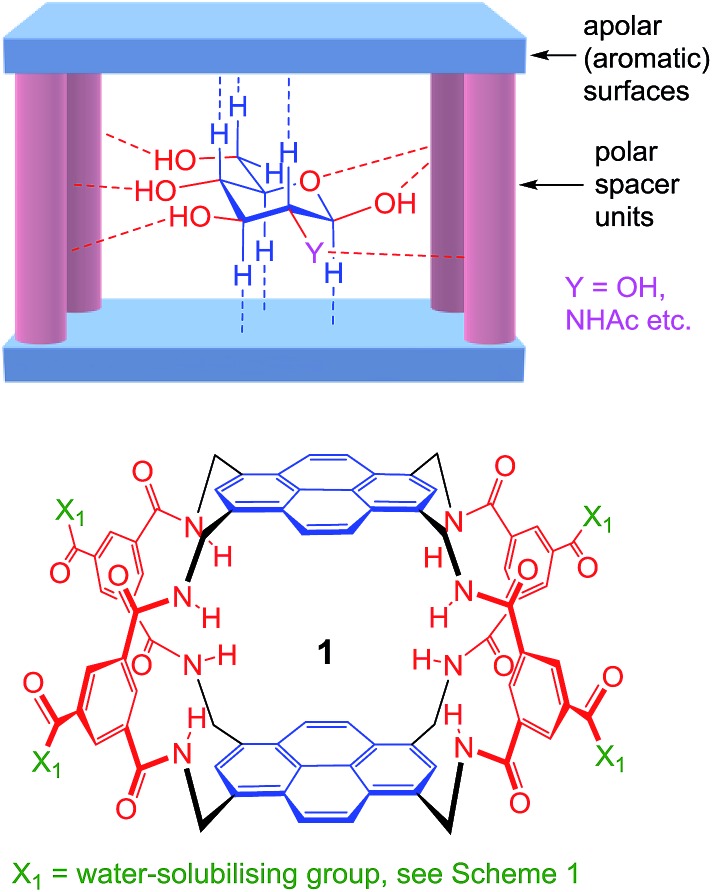
General approach to synthetic lectins for all-equatorial carbohydrates (β-glucose, β-GlcNAc *etc.*), with recent achiral example **1** (see ref. 14a).

As illustrated in Fig. 1, our receptors possess a common architecture in which parallel aromatic surfaces (blue) are separated by rigid polar pillars (red). The aromatic surfaces make hydrophobic/CH-π contacts with substrate CH groups, while the pillars form hydrogen bonds to polar equatorial substituents. In line with the cartoon in Fig. 1, we have named these molecules the “temple” family of carbohydrate receptors.^1*b*^

**Fig. 2:**
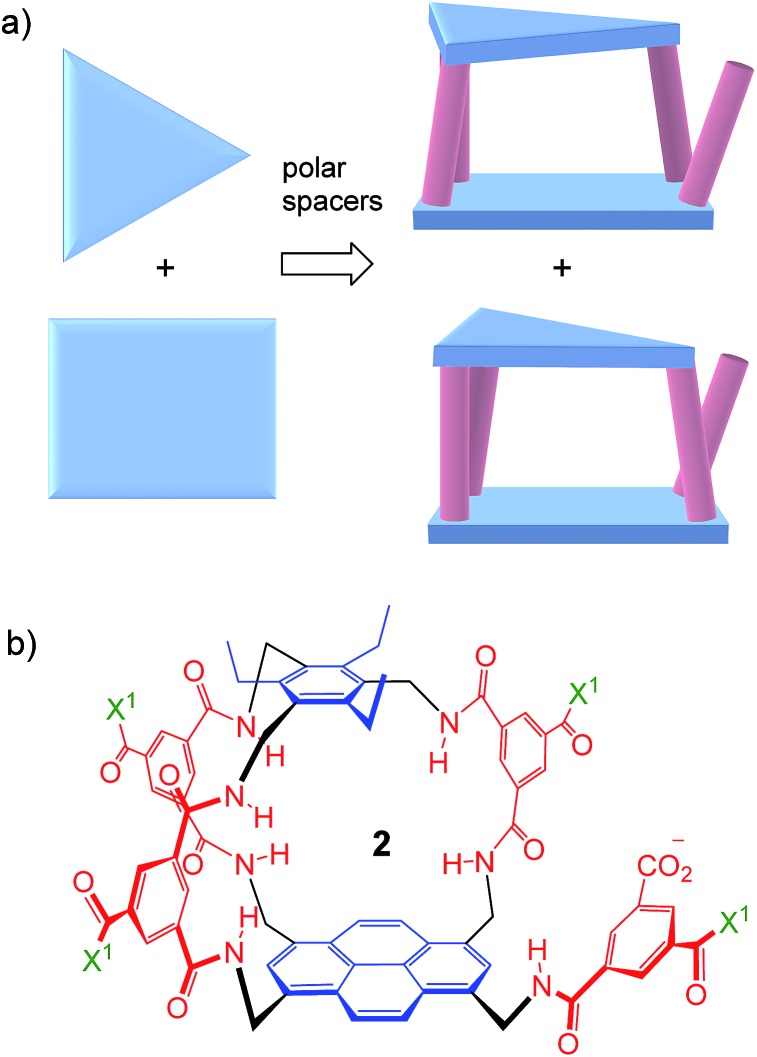
(a) Chiral receptor architecture derived by combining *C*_3_ and *D*_2_ roof/floor components. (b) Specific design **2** featured in this paper. See Scheme 1 for the structure of water-solubilising group X^1^. The framework of **2** is planar chiral and the enantiomer shown is p*S* according to standard nomenclature (see Fig. S35, ESI†).

Thus far, all variants of the temple design have been achiral, with at least one plane of symmetry. However, as illustrated in Fig. 2a, it is possible to generate asymmetric versions by employing roof and floor units of different symmetries. In particular, a *C*_3_-symmetric roof can be combined with a square or rectangular (*D*_2_) floor in just two ways, giving a pair of enantiomeric products. Acting as receptors, these molecules would surround their substrates with chiral cage frameworks which seem likely to favour enantioselectivity. The synthesis would leave an unreacted spacer unit, but this can be quenched in various ways and could be used to advantage.

Herein we report the realisation of this concept in the form of macrobicycle **2** and its enantiomer (Fig. 2b). We show that these prototypes are capable of high levels of enantioselectivity in water, matching the discrimination shown by other systems in unnatural non-aqueous media. In addition, new records are set for binding affinity to simple monosaccharides, suggesting that chirality can improve complementarity in the design of synthetic carbohydrate receptors.

## Results and discussion

**Scheme 1:**
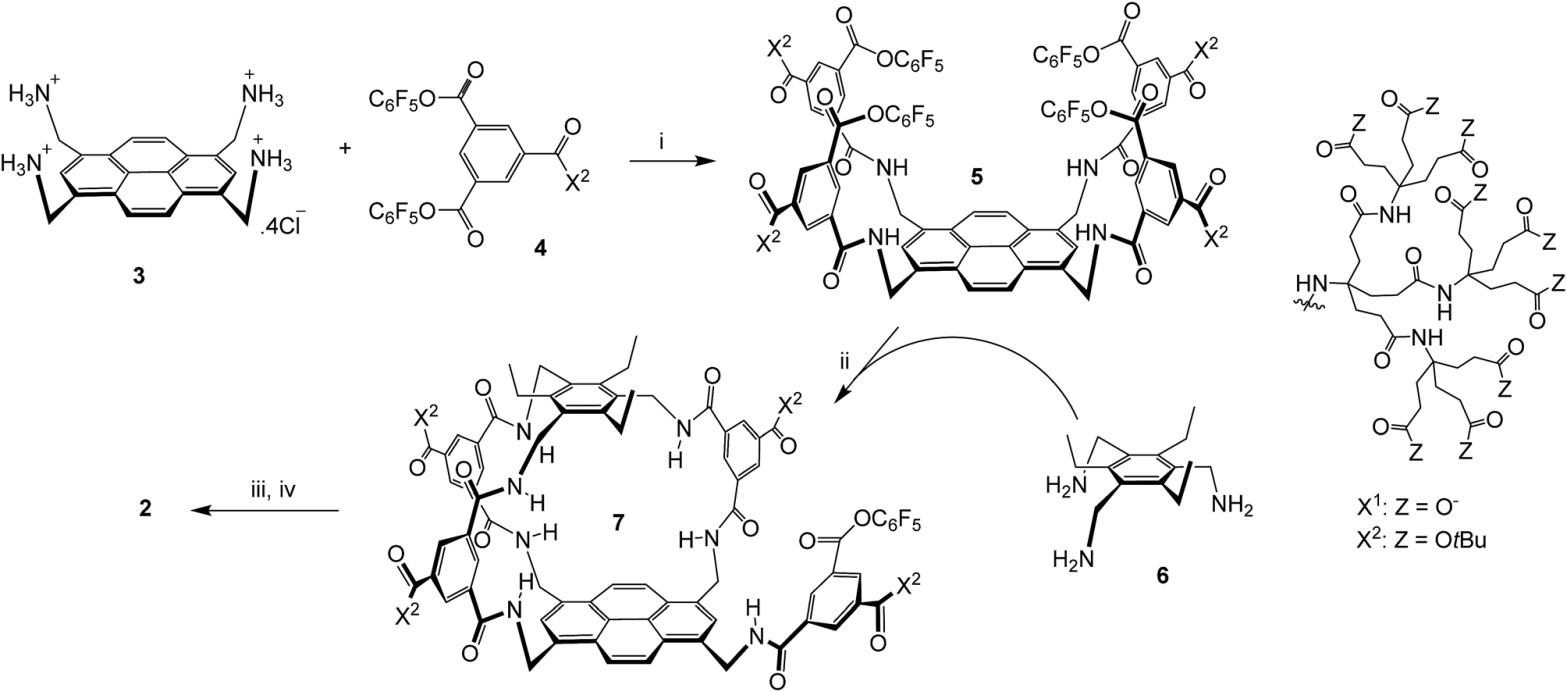
Synthesis of (±)-**2**. (i) THF/water, **4** (8 equivalents), EtN(*i*Pr)_2_, 47%; see ref. 14a. (ii) THF, EtN(*i*Pr)_2_, [**5**] and [**6**] ≤ 0.11 mM, 51%. (iii) TFA, DCM. (iv) NaOH, H_2_O, then Amberlyst 15 hydrogen form, then NaOH (to pH = 7).

The sequence employed to synthesise (±)-**2** is shown in Scheme 1. Intermediate **5** was prepared from protonated tetra-amino pyrene **3** and isophthalate reagent **4**, as reported previously for the synthesis of **1**.^14*a*^ Active ester **5** was then combined with triamine **6** under high dilution to give (±)-**7** in the remarkably good yield of 51%. Triamine **6** was chosen as roof component in the expectation that the ethyl groups would preorganise the amines and improve yields;^17^ this scaffold has previously been employed to construct carbohydrate receptors operating in organic solvents.^18^ The side-chain *t*-butyl esters were then cleaved with TFA, and the unreacted pentafluorophenyl ester was converted to carboxyl by basic hydrolysis. Finally the pH was adjusted to 7 using acidic ion exchange resin and NaOH, to give (±)-**2** as a salt-free racemate.

Receptors **2** gave well-resolved ^1^H NMR spectra in D_2_O with minimal changes on dilution below 0.5 mM, implying that they do not self-aggregate in this concentration range (Fig. S13 and S24†). The spectra were more complex than those of previous synthetic lectins, reflecting the asymmetry of the framework. A full assignment of signals due to framework CH protons could be made using 2D NMR spectroscopy (Fig. S14–S23†).
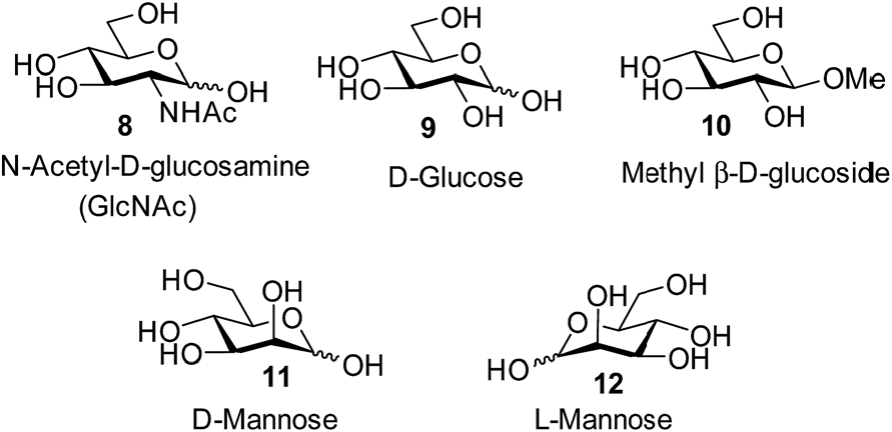


**Fig. 3:**
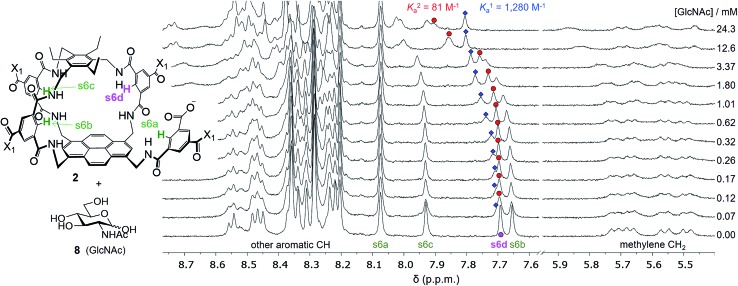
Selected partial spectra for a ^1^H NMR binding study of receptors (±)-**2** (0.15 mM each) with d-GlcNAc **8** in D_2_O. The labelling system used for **2** and a full NMR assignment are detailed in the ESI.† Signals due to protons s6a–d appear in the region 7.6–8.1 ppm and are readily observed during the titration. In particular, the signal due to s6d splits into two peaks which can be followed throughout.

**Fig. 4:**
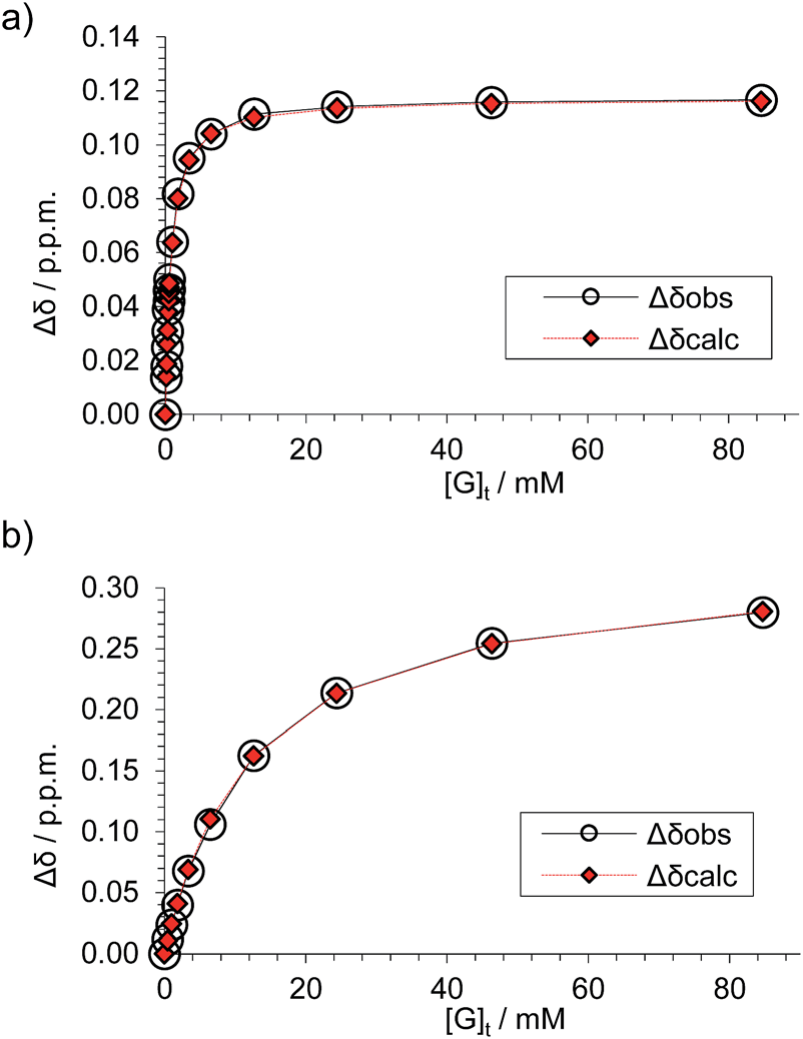
Data analyses for the ^1^H NMR titration of (±)-**2** with d-GlcNAc **8** (see Fig. 3) assuming a 1 : 1 binding model. The signal due to proton s6d was followed for both diastereomeric complexes. (a) Analysis of peaks marked by blue diamonds in Fig. 3 spectra; *K*_a_ = 1280 M^–1^ ± 2%, limiting Δ*δ* = 0.117 ppm. (b) Analysis of peaks marked by red circles in Fig. 3 spectra, *K*_a_ = 81 M^–1^ ± 5%, limiting Δ*δ* = 0.331 ppm.

Attempts to separate the enantiomers of **2** were unsuccessful,^19^ but the binding properties of both receptors could be studied through ^1^H NMR studies on the racemate. Addition of carbohydrates **8–12** to (±)-**2** caused substantial movements of receptor signals, many splitting as expected for the formation of diastereomeric complexes (one for each enantiomer of **2**). The inward-directed spacer CH signals s6b–d (see Fig. 2) were relatively shielded in free **2** and showed especially large changes during the titrations. The titration with GlcNAc (**8**) was particularly informative (Fig. 3 and S25†). In this case signals due to spacer protons s6d (see Fig. 3) could be followed throughout the titration for both receptor enantiomers. Both sets of data gave excellent fits to a 1 : 1 binding model, yielding binding constants *K*_a_ of 1280 M^–1^ and 81 M^–1^ for the diastereomeric complexes (Fig. 4).^20^

These values are significant in two respects. Firstly, the enantioselectivity of 16 : 1 matches the highest previously measured for monosaccharide binding by synthetic receptors in any medium.^12i,k^ Secondly, the affinity for the more tightly-bound complex is the highest yet observed for this substrate, breaking the record of 520 M^–1^ previously held by the bis-pyrenyl system **13** (the “staggered” regioisomer of **1**).^14*a*^ Indeed, it appears to be the highest for biomimetic recognition of any underivatised, uncharged^21^ monosaccharide. It also compares well with the binding constant of 410 M^–1^ measured for the lectin Wheat Germ Agglutinin (WGA) for the same substrate.^22^ Notably, the high affinity of (one enantiomer of) **2** for **8** is achieved without the benefit of statistical factors available to receptors of higher symmetry. For example, in *D*_2h_ receptors such as **1** there are four equivalent orientations for a carbohydrate within the binding site, each contributing to the binding affinity.^23^ For chiral receptor **2** there is no degeneracy. The additional binding energy from improved complementarity must therefore be sufficient to compensate for this loss.

Although the receptors **2** could not be resolved, the separation of signals due to the diastereomeric complexes presented an opportunity to assign the structure of the stronger binding enantiomer. NMR studies were performed on a mixture of (±)-**2** and **8**, chosen so that receptor signals were well-separated and that the more strongly-bound complex was present at relatively high concentrations. NOESY and TOCSY spectra allowed a nearly complete assignment of signals due to the more strongly bound enantiomer of **2**. Intermolecular NOE signals indicated that the carbohydrate CH_2_OH was positioned in the smallest of the three portals of the receptor (between s6b and s6c), with the α-face directed towards the pyrene. Further connections led to the conclusion that stronger-binding enantiomer was p*R*-**2** (*i.e.* the antipode of the structure in Fig. 2b). Details of the assignment and spectra are given in the ESI (see Fig. S35–S55†).
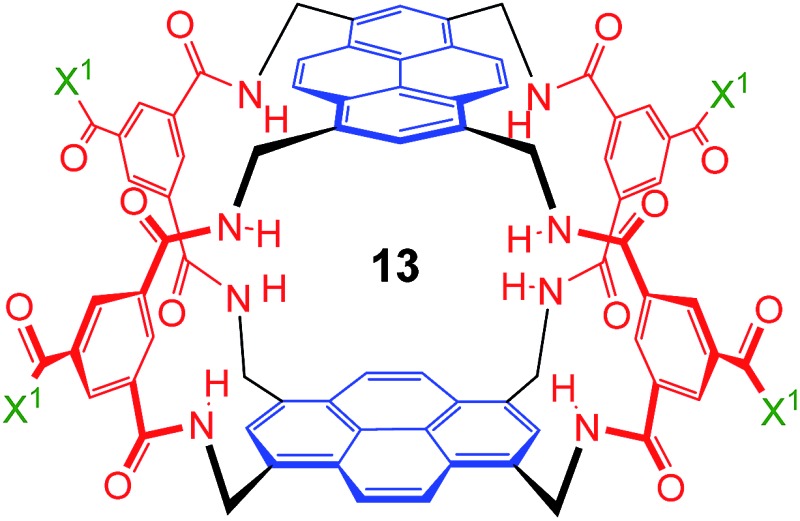


The other all-equatorial carbohydrates **9** and **10** proved more difficult to study, although enantiodiscrimination was apparent for both substrates. In the case of glucose **9**, addition to (±)-**2** caused receptor ^1^H NMR signals to move and split as expected for diastereomeric complex formation, but those due to one diastereomer then broadened (presumably due to slow exchange) and could not be followed (Fig. S27†). A signal from the second complex could be analysed, yielding a binding constant of 250 M^–1^ (Fig. S28†).^20^ Again, this breaks previous records for biomimetic recognition of this important substrate; earlier studies have yielded values up to 190 M^–1^, again for **13**.^14*a*^ Moreover, while the signals for the second diastereomeric complex could not be analysed, initial movements occurred early during the titration suggesting that the affinity may be still higher.^17^ Similar changes were observed when methyl β-d-glucoside **10** was added to **2**. In this case two signals could be followed to the end of the titration, but both appeared to belong to one diastereomeric complex and gave *K*_a_ = 250 M^–1^ on analysis (Fig. S29 and S30†).

The non-target substrate mannose was studied both as single enantiomer **12** and as a racemate, formed by mixing **11** and **12**. For the single enantiomer, the formation of both diastereomeric complexes could be followed, with *K*_a_ = 7.6 and 2.3 M^–1^ respectively (Fig. S31 and S32†). For racemic mannose + racemic **2** a single set of signals was observed, as expected for two racemic diastereomeric complexes equilibrating rapidly on the ^1^H NMR timescale. Analysis gave an apparent *K*_a_ of 4.8 M^–1^, close to the average of the separately-measured values (Fig. S33 and S34†). The selectivity for all-equatorial substrates **8–10** *vs.* mannose **11**/**12** is consistent with previous work on the “temple” family of receptors.^1b,14^

## Conclusions

In conclusion we have demonstrated a straightforward, high-yielding method for introducing chirality into the framework of a synthetic lectin. The approach has yielded the first synthetic receptors capable of enantioselective carbohydrate recognition in water through the application of purely non-covalent interactions. Moreover the level of enantioselectivity matches that achieved by earlier systems in non-aqueous (and therefore unnatural) media. For matched enantiomers, the receptors are also exceptionally powerful. The highest affinity measured, 1280 M^–1^ for GlcNAc, is more than twice that for any earlier receptor with the same substrate, and three times that for the lectin WGA. This success may reflect the principle that a chiral substrate requires a chiral receptor for ideal complementarity, even though low symmetry incurs an entropic penalty.^23^

The strategy employed to make **2** also allows generalization to other “roofs”, through use of different triamines in the cyclisation step. All such systems would require resolution, and experience with **2** suggests that the problem may not be trivial. However for receptors with potential applications solutions could surely be found. Further elaboration should also be feasible through nucleophilic attack on the unreacted active ester groups in intermediates such as **7**. For example, this would provide a ready means of attachment to a polymer support, to generate a carbohydrate-selective stationary phase. Alternatively, the binding site could be supplemented by attaching peptidic units, potentially in combinatorial format.^24^ This approach thus seems promising for the development of improved synthetic lectins for β-glucosyl, β-GlcNAc and related “all-equatorial” carbohydrates.
