# Supplementary material for: Enantioselective carbohydrate recognition by synthetic lectins in water
Source: Chem Sci. 2017 Mar 30;8(5):4056–61. doi: 10.1039/c6sc05399h (PMC5465552; doi:10.1039/c6sc05399h)
Supplement: Supplementary file 1 [file SC-008-C6SC05399H-s001.pdf]

Electronic Supplementary Material (ESI) for Chemical Science.
This journal is © The Royal Society of Chemistry 2017

Supplementary Information

Enantioselective Carbohydrate Recognition by Sythetic Lectins in Water

Pablo Ríos, Tiddo J. Mooibroek,* Tom S. Carter, Christopher Williams, Miriam R. Wilson, Matthew P. Crump and Anthony P. Davis*

School of Chemistry, University of Bristol, Cantock's Close, Bristol BS8 1TS, United Kingdom.

Table of Contents

1. Synthesis and characterizations.. S2

Synthesis of protected receptors (±)-**7**.. S4
Characterization of protected receptors (±)-**7**... S6
Synthesis of water-soluble receptors (±)-**2**.. S16
Characterization of water-soluble receptors (±)-**2**..................................... S18

2. ^{1}H NMR titrations.. S30

With *N*-acetyl-D-glucosamine **8**.. S31
With D-glucose **9**.. S33
With methyl-β-D-glucoside **10**... S35
With L-mannose **12**.. S37
With 1:1 D- and L-mannose **12**... S39

3. Identifying the stronger-bound enantiomer from the (±)2 + 8 titration S41

Summary S41
NMR spectra and assignments of *N*-acetyl-D-glucosamine **8**.................... S47
NMR spectra and assignments of of (±)-**2** + **8**.. S53

1. Synthesis and characterisations

All commercially available chemicals were purchased from Sigma-Aldrich, Alfa-Aesar, Acros Organics, ABCR or Carbosynth Ltd., and used without further purification, unless stated otherwise. Solvents were utilized as supplied unless otherwise stated. Anhydrous solvents (e.g. THF or CH_2Cl_2) were dried by passing through a modified Grubbs system[1] manufactured by Anhydrous Engineering. Anhydrous DMF and DMSO were dried by vacuum distillation from P_2O_5 and CaH_2, respectively.[2] Flash column chromatography was carried out using chromatography grade silica 60 Å (Sigma-Aldrich, particle size 35-70 micron). TLC analysis was performed using pre-coated silica gel TLC plates (Merck silica gel 60 F254). Spots were visualised by means of UV light (254 or 365 nm) or using solutions of phosphomolybdic acid, alizarin, potassium permanganate, or ninhydrin. HPLC chromatography was performed using a Waters 600 controller with a Waters 2998 photodiode array detector. For analytical runs a XSELECT CSH C_{18} 5 μm (4.6 × 150 mm) column was used, and for preparative runs a XSELECT CSH Prep C_{18} 5 μm OBD (19 × 250 mm) column was utilised.

^{1}H-, ^{13}C-, ^{19}F-, and 2D-NMR spectra were acquired at 298 K (unless otherwise specified) at 400, 500, or 600 MHz using the following spectrometers: Jeol ECS 400, Varian 400-MR, Varian VNMRS 500 equipped with a triple resonance ^{1}H observe probe or, X-observe probe, or a Varian VNMRS 600 equipped with a cryogenically cooled ^{1}H-observe triple resonance probe. Chemical shifts (δ) are reported in parts per million (p.p.m.). Residual solvent resonances were used as internal reference for δ-values in ^{1}H-, and ^{13}C-NMR,[3] while ^{19}F-NMR spectra were externally referenced to CF_3COOH (–76.55 p.p.m.). ESI-HRMS (electrospray ionisation high resolution mass spectrometry) was performed on a Bruker Daltonics micrOTOF II and MALDI-MS (matrix-assisted laser desorption/ionisation) was performed on an Applied Biosystems 4700 or a Bruker Daltonics UltrafleXtreme.

---

[1] A. B. Pangborn, M. A. Giardello, R. H. Grubbs, R. K. Rosen and F. J. Timmers, *Organometallics*, 1996, **15**, 1518-1520.

[2] W. F. Armarego and C. L. L. Chai, *Purification of Laboratory Chemicals*, Elsevier, Oxford, 2009.

[3] G. R. Fulmer, A. J. M. Miller, N. H. Sherden, H. E. Gottlieb, A. Nudelman, B. M. Stoltz, J. E. Bercaw and K. I. Goldberg, *Organometallics*, 2010, **29**, 2176-2179.

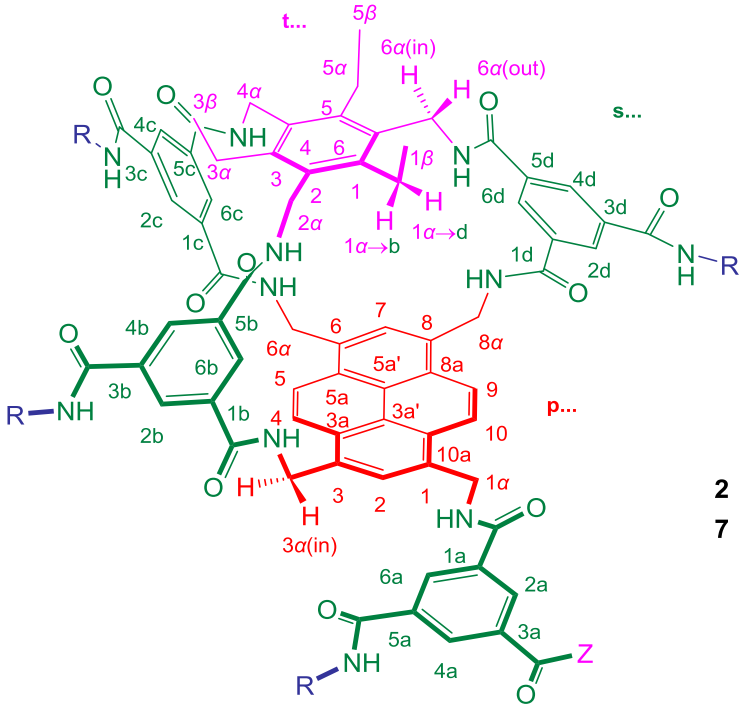

2 Z = ONa, R = R_1

7 Z = OC_6F_5, R = R_2

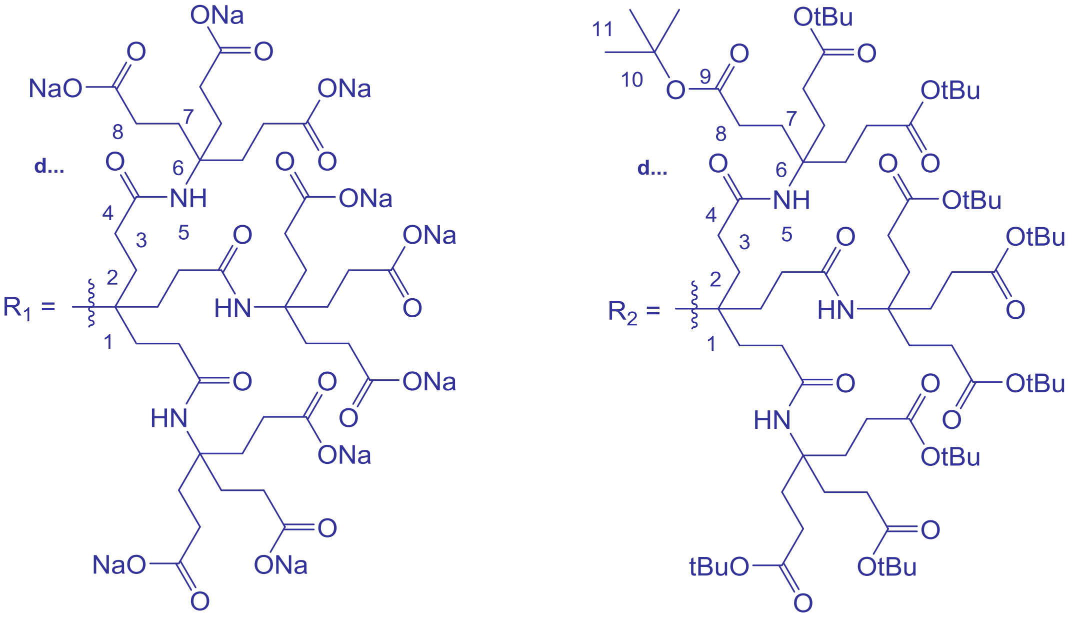

Figure S1. Numbering system for receptors (±)-**2** and precursors (±)-**7**, as used for the assignment and discussion of NMR spectra. Pyrenyl proton designations (red) are prefixed with “p” (i.e. p4, p1α etc.) while protons from the different spacers (green) are prefixed with an “s” and suffixed by “a,b,c,d” to denote which of the four spacers is involved. Protons from the triamine unit (pink) are prefixed “t” and protons from the dendritic side-chains (blue) are prefixed with “d”. Special designations are used for some benzylic hydrogens (e.g. t1α→b is the proton on ethyl group 1 closer to spacer b).

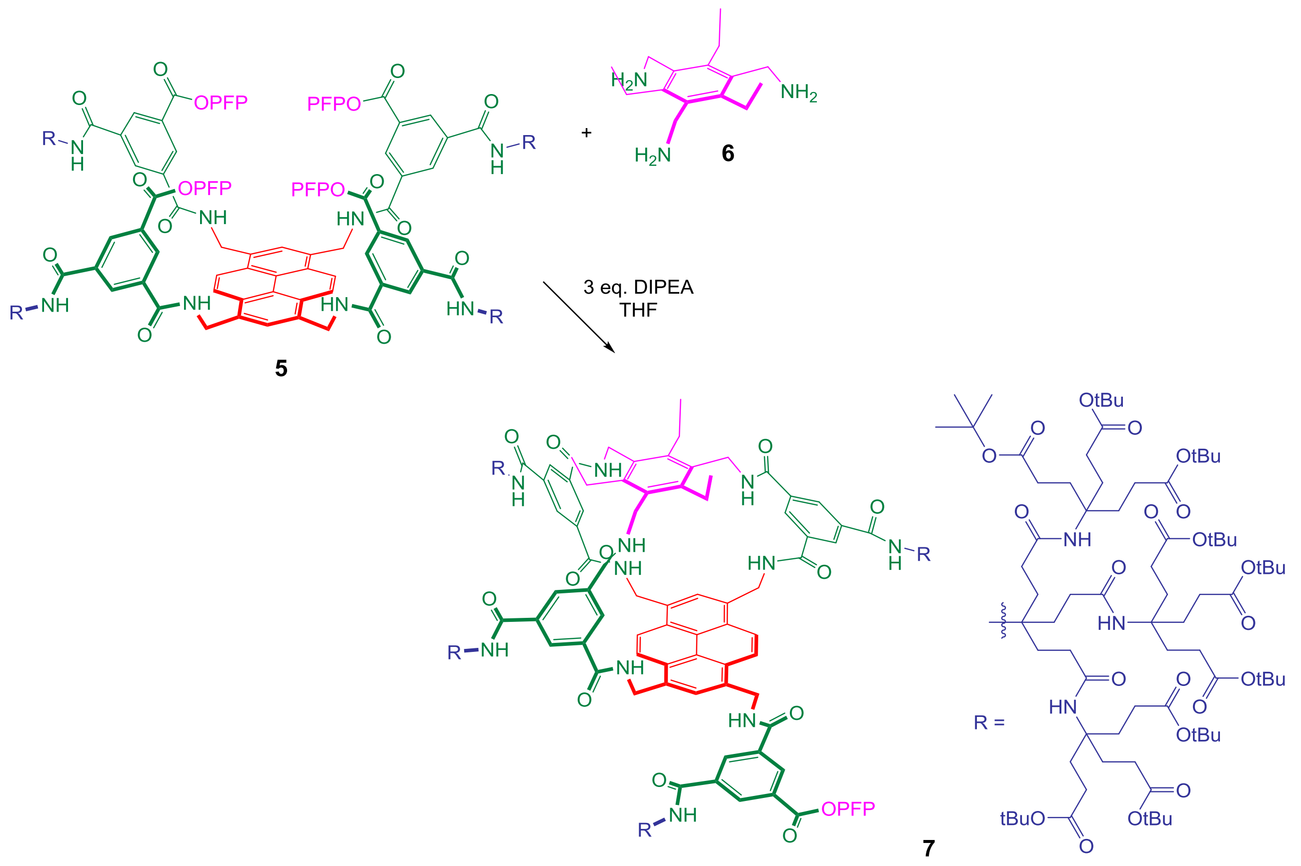

Protected receptors (±)-7. A 500 mL round-bottom flask was charged with a magnetic stirrer bar, tetraester **5**[4] (158 mg, 21.2 μmol) and dry and N_2-saturated tetrahydrofuran (THF, 190 mL). A solution of 1,3,5-tris(aminomethyl)-2,4,6-triethylbenzene (5.3 mg, 21.2 μmol) in dry and N_2-saturated THF (20 mL) and N,N'-diisopropylethylamine (DIPEA, 10.5 μL, 63.6 µmol) was then added in a dropwise manner over 12 h using a syringe pump. After stirring for 12 more hours, saturated ammonium chloride (1 mL) was added to the reaction mixture, and the mixture was left stirring for 10 min. The solvent was then evaporated to a volume of around 10 mL. The remaining suspension was extracted with dichloromethane (DCM, 3 x 30 mL) and the organic phase was washed with H_2O (3 x 30 mL), dried over $MgSO_4$, filtered and concentrated. The residue was dissolved in acetone/water (70:30), passed through a 0.2 µm filter, injected into a preparative HPLC apparatus fitted with a reverse phase column and eluted with acetone/water (70:30 to 85:15 over 15 min, 85:15 to 100:0 over 60 min and then 100:0 for further 5 min). The component eluting at 46 min was collected (see Figure S2 for trace), concentrated using a rotary evaporator (T < 30 °C) and freeze-dried to yield protected receptors (±)-**7** as a pale yellow solid (77.5 mg, 10.9 μmol, 51%). The products were identified by 1 dimensional ^{1}H and ^{19}F-NMR as well as 2 dimensional {^{1}H-^{1}H}-NOESY, {^{1}H-^{1}H}-COSY, and {^{1}H-^{13}C}-HSQC, as detailed below and in Figure S3 - Figure S12. ^{1}H NMR (CH_3OD, 600 MHz): δ = 1.10 (m, 1H, t1β), 1.12 (m, 1H,

[4] P. Rios, T. S. Carter, T. J. Mooibroek, M. P. Crump, M. Lisbjerg, M. Pittelkow, N. T. Supekar, G.-J. Boons and A. P. Davis, *Angew. Chem., Int. Ed.*, 2016, **55**, 3387-3392.

t3β), 1.14 (m, 1H, t5β), 1.4 (s, 324H, d11), 1.75 – 2.43 (m, 192H, d2+d3+d7+d8), 2.44 (m, 1H, t1α→d), 2.47 (m, 1H, t1α→b), 2.58 (m, 1H, t3α→b), 2.62 (m, 1H, t5α→d), 2.64 (m, 1H, t3α→c), 2.67 (m, 1H, t5α→c), 4.19 (d, 1H, *J* = 12.9 Hz, t2α(in)), 4.23 (d, 1H, *J* = 13.2 Hz, t6α(out)), 4.58 (d, 1H, *J* = 14 Hz, t4α(in)), 4.59 (d, 1H, t6α(in)), 4.73 (d, 1H, t2α(out)), 4.77 (d, 1H, p6α(in)), 4.85 (d, 1H, t4α(out)), 4.86 (d, 1H, p8α(in)), 4.98 (d, 1H, *J* = 15.8 Hz, p1α(in)), 4.98 (d, 1H, *J* = 13.9 Hz, p3α(in)), 5.55 (d, 1H, *J* = 15.7 Hz, p1α(out)), 5.63 (d, 1H, *J* = 13.8 Hz, p8α(out)), 5.71 (d, 1H, *J* = 15.1 Hz, p3α(out)), 5.78 (d, 1H, *J* = 14 Hz, p6α(out)), 7.70 (s, 1H, s6b), 7.72 (s, 1H, s6d), 8.02 (s, 1H, s6c), 8.24 (s, 1H, p2), 8.27 (s, 1H, p7), 8.40 (d, 1H, *J* = 10.8 Hz, p10), 8.43 (s, 1H, s4b), 8.46 (d, 1H, *J* = 10.8 Hz, p5), 8.48 (d, 1H, p9), 8.48 (s, 1H, s2b), 8.50 (s, 1H, s4d), 8.56 (s, 1H, s2c), 8.57 (s, 1H, s2d), 8.64 (d, 1H, *J* = 9 Hz, p4), 8.65 (s, 1H, s4c), 8.69 (s, 1H, s2a), 8.82 (s, 1H, s6a), 8.86 (s, 1H, s4a) p.p.m.; ^{19}F NMR (CH$_3$OD, 470 MHz) δ = -154.49 (d, 2F, *J* = 19.2 Hz, oF), -160.32 (m, 1F, pF), -164.75 (m, 2F, mF) p.p.m.; ^{13}C-NMR (CD$_3$OD, 125 MHz): δ = 17.38 (t3β), 17.44 (t5β), 17.76 (t1β), 24.59 (t3α), 24.76 (t5α), 25.64 (t1α), 40.96 (t2α), 41.08 (t4α), 41.29 (t6α), 43.79 (p3α), 44.29 (p1α), 44.64 (p6α), 46.26 (p8α), 125.72 (p5), 125.73 (p10), 126.51 (p9), 126.62 (p4), 129.33 (s6c), 129.52 (s6b), 129.62 (s6d), 131.36 (s2d), 131.41 (s4b), 132.41 (p2), 132.50 (s2c), 132.51 (s2b), 132.61 (s4c), 132.69 (s4d), 134.00 (s4a), 134.76 (p7), 134.83 (s2a), 134.90 (s6a) p.p.m.. HRMS (ESI): m/z calculated for $C_{381}H_{586}F_5N_{23}O_{97}NaK$ [M+Na$^+$+K$^+$]$^{2+}$: 3596.06, found 3595.80.

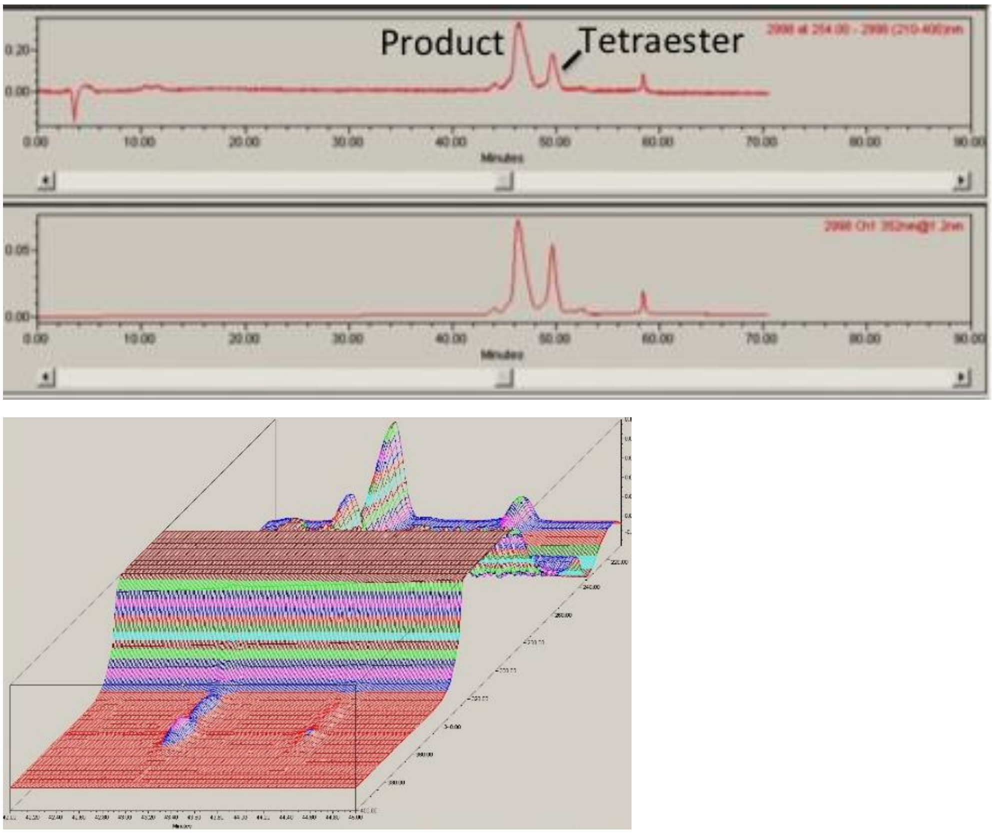

Figure S2. UV-Vis Chromatograms from preparative HPLC of protected receptors (±)-**7**. Top left = 254 nm; bottom left = 352 nm; right = 3D chromatogram.

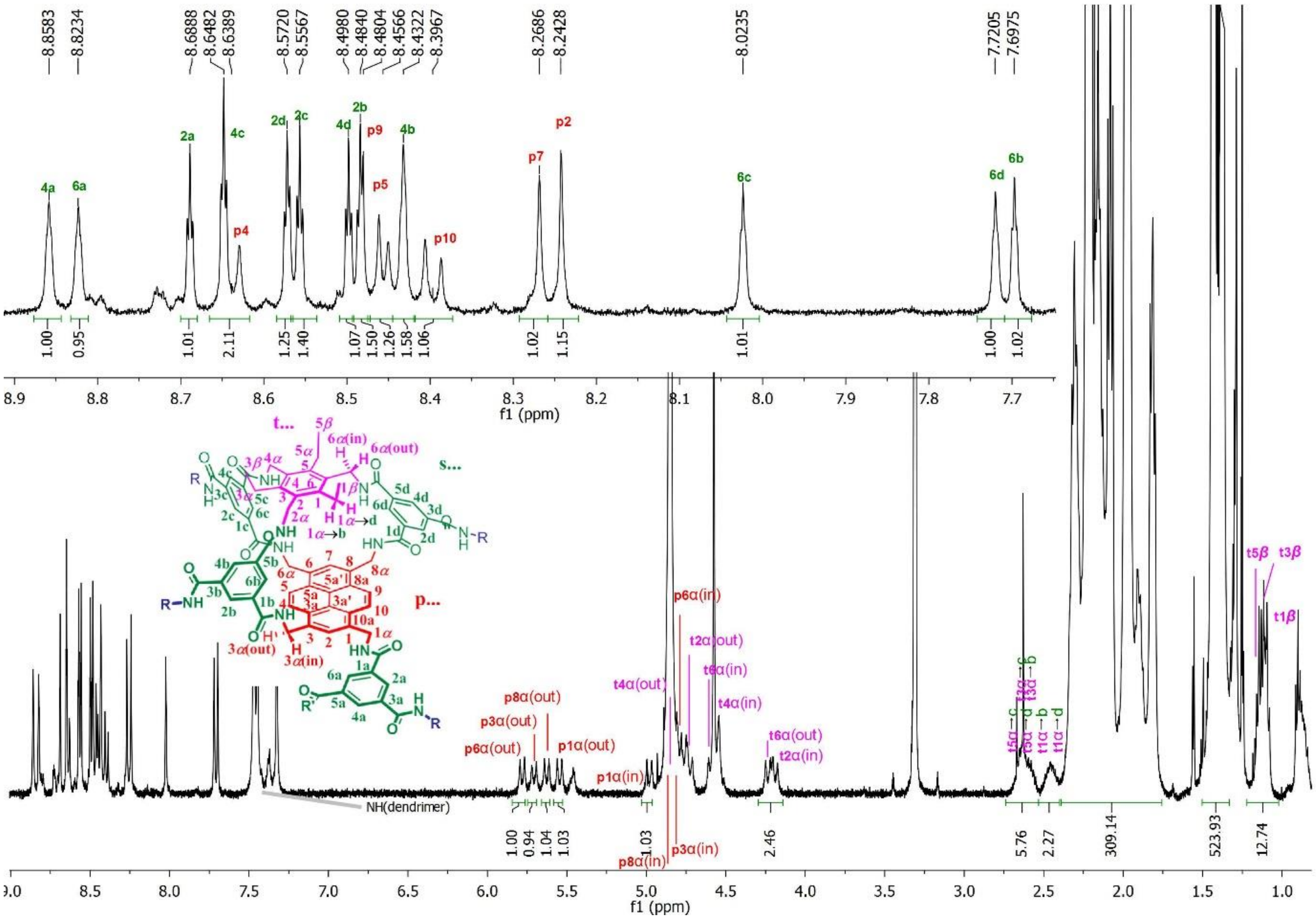

Figure S3. ^{1}H-NMR (600 MHz) in CD_3OD for protected receptors (±)-**7**.

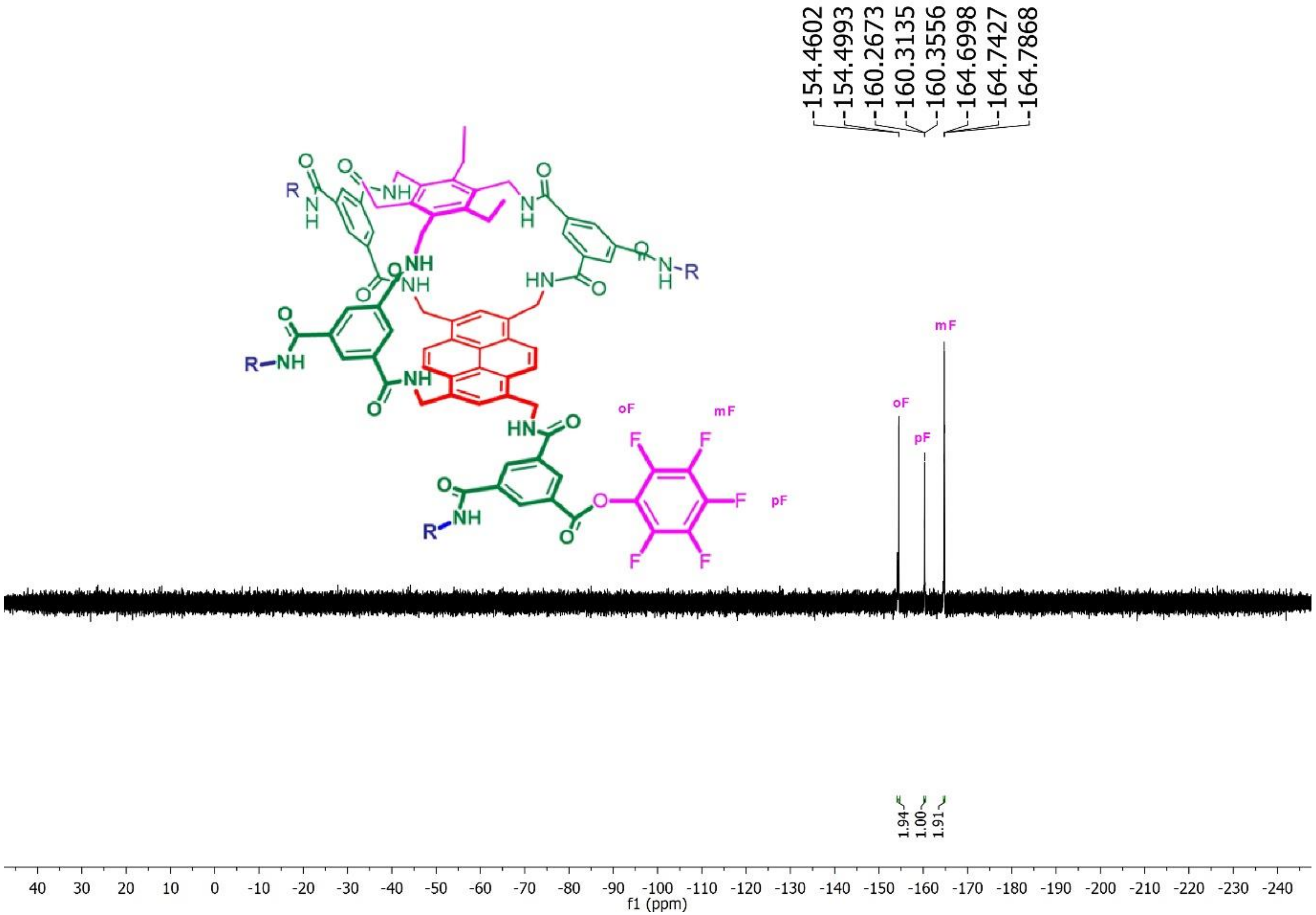

Figure S4. ^{19}F-NMR (470 MHz) in CD_3OD for protected receptors (±)-**7**.

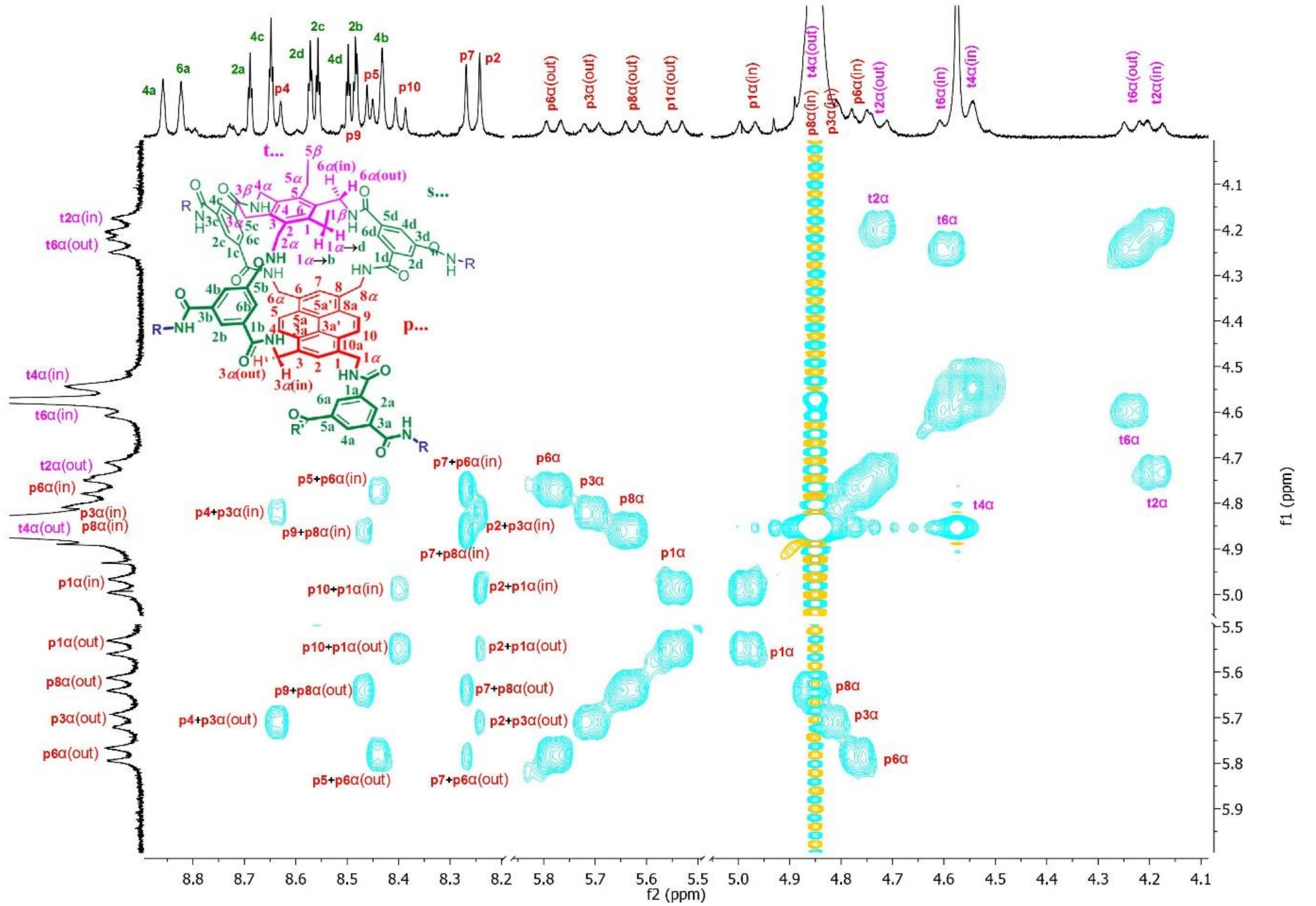

Figure S5. Partial {^{1}H-^{1}H}-NOESY (600 MHz) in CD_3OD for protected receptors (±)-**7**.

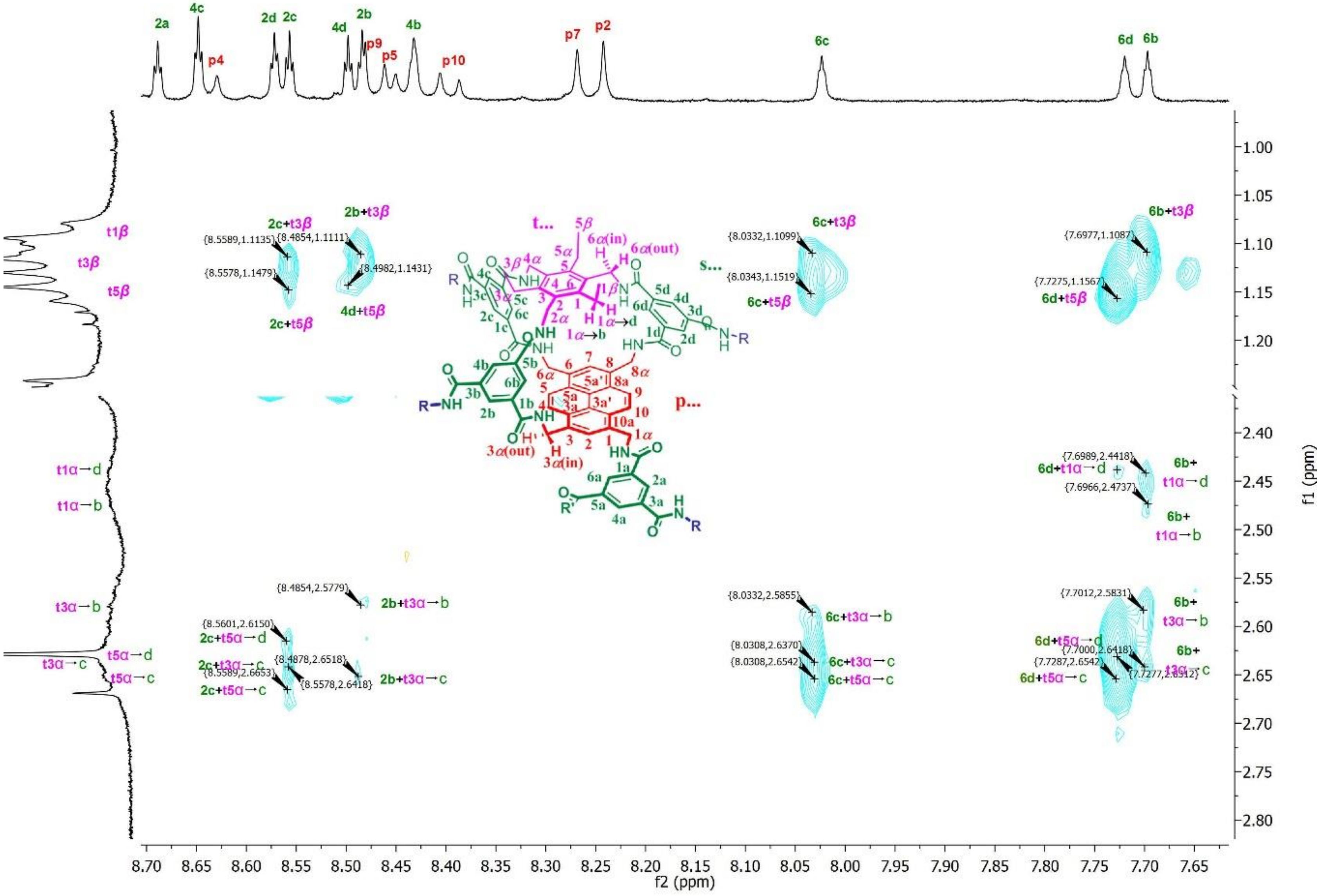

Figure S6. Partial {^1H-^1H}-NOESY (600 MHz) in CD_3OD for protected receptors (±)-**7**.

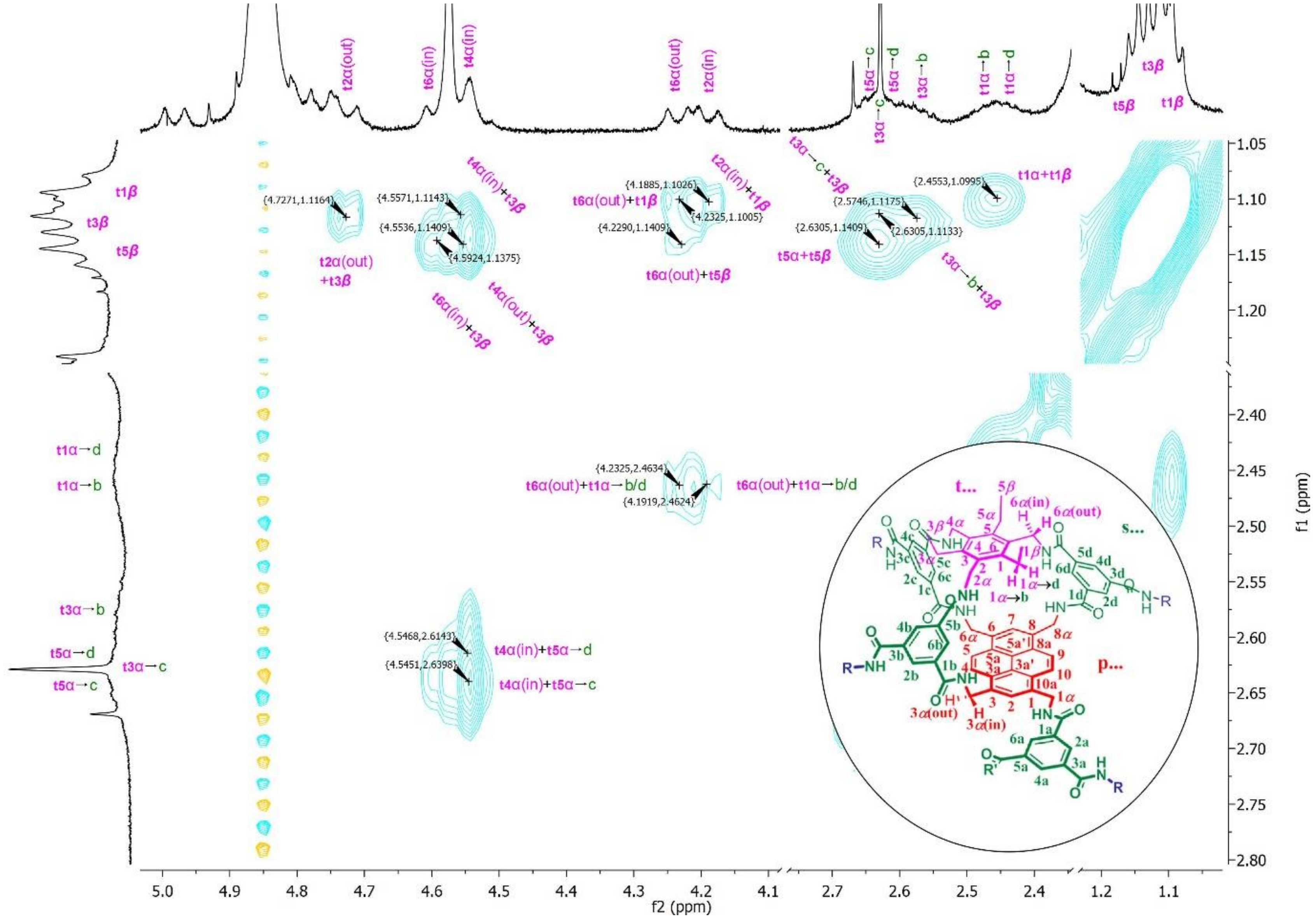

Figure S7. Partial {^{1}H-^{1}H}-NOESY (600 MHz) in CD_3OD for protected receptors (±)-**7**.

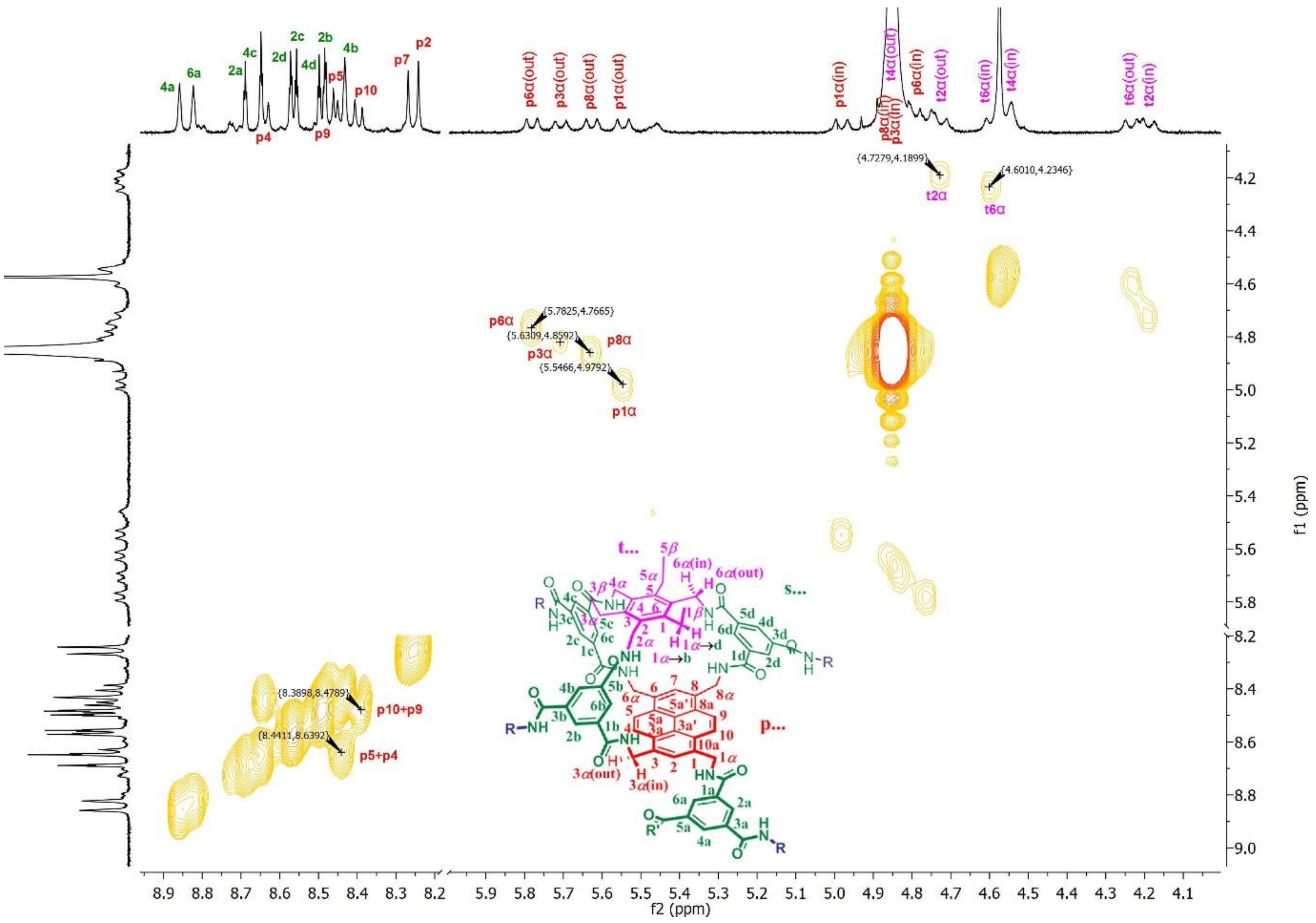

Figure S8. Partial {^1H-^1H}-COSY (600 MHz) in CD_3OD for protected receptors (±)-**7**.

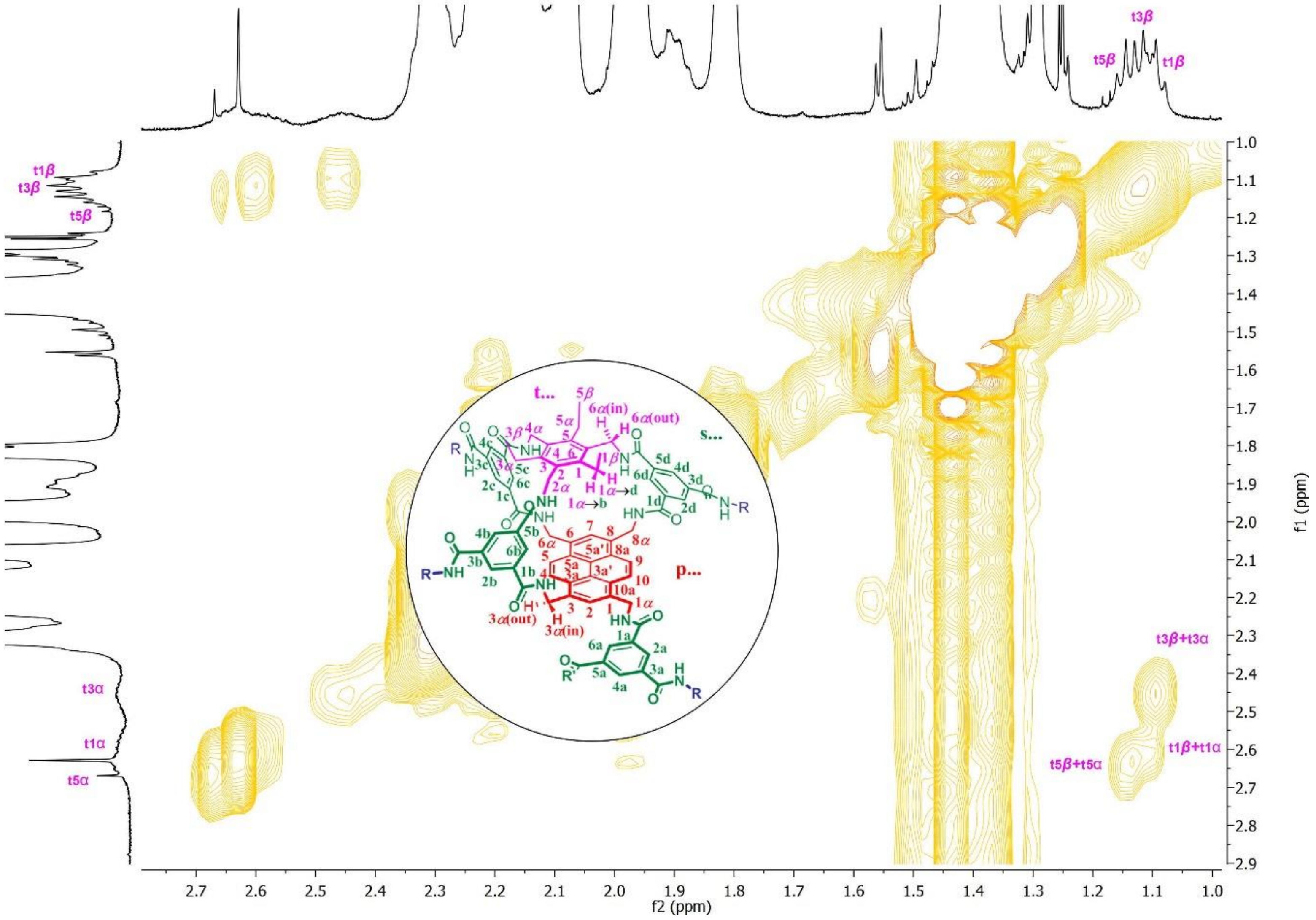

Figure S9. Partial {^{1}H-^{1}H}-COSY (600 MHz) in CD_3OD for protected receptors (±)-**7**.

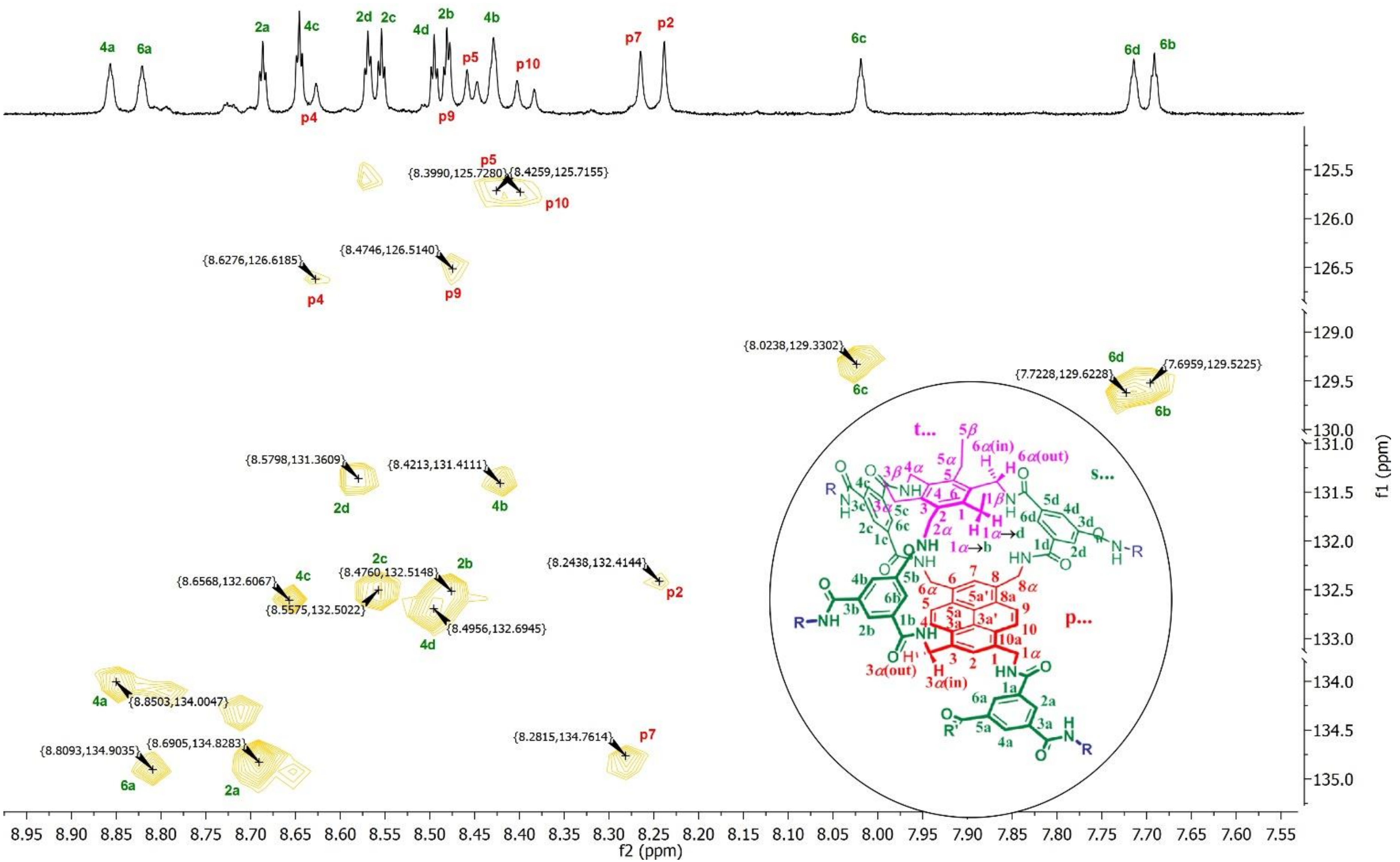

Figure S10. Partial {^{1}H-^{13}C}-HSQC (600 MHz) in CD_3OD showing cross-peaks for aromatic signals of protected receptors (±)-**7**.

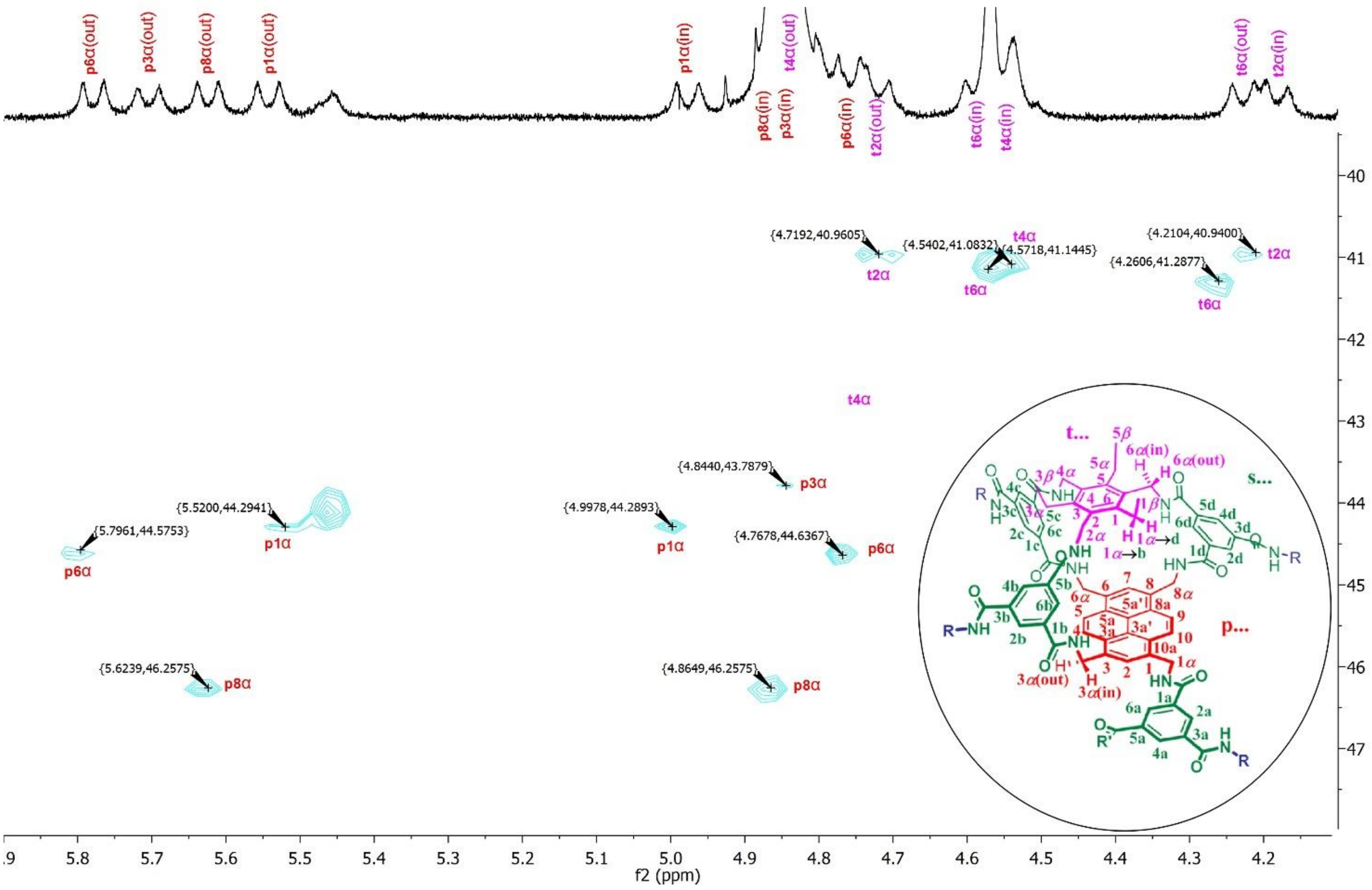

Figure S11. Partial {^{1}H-^{13}C}-HSQC (600 MHz) in CD_3OD showing cross-peaks for CH_2 signals of protected receptors (±)-**7**.

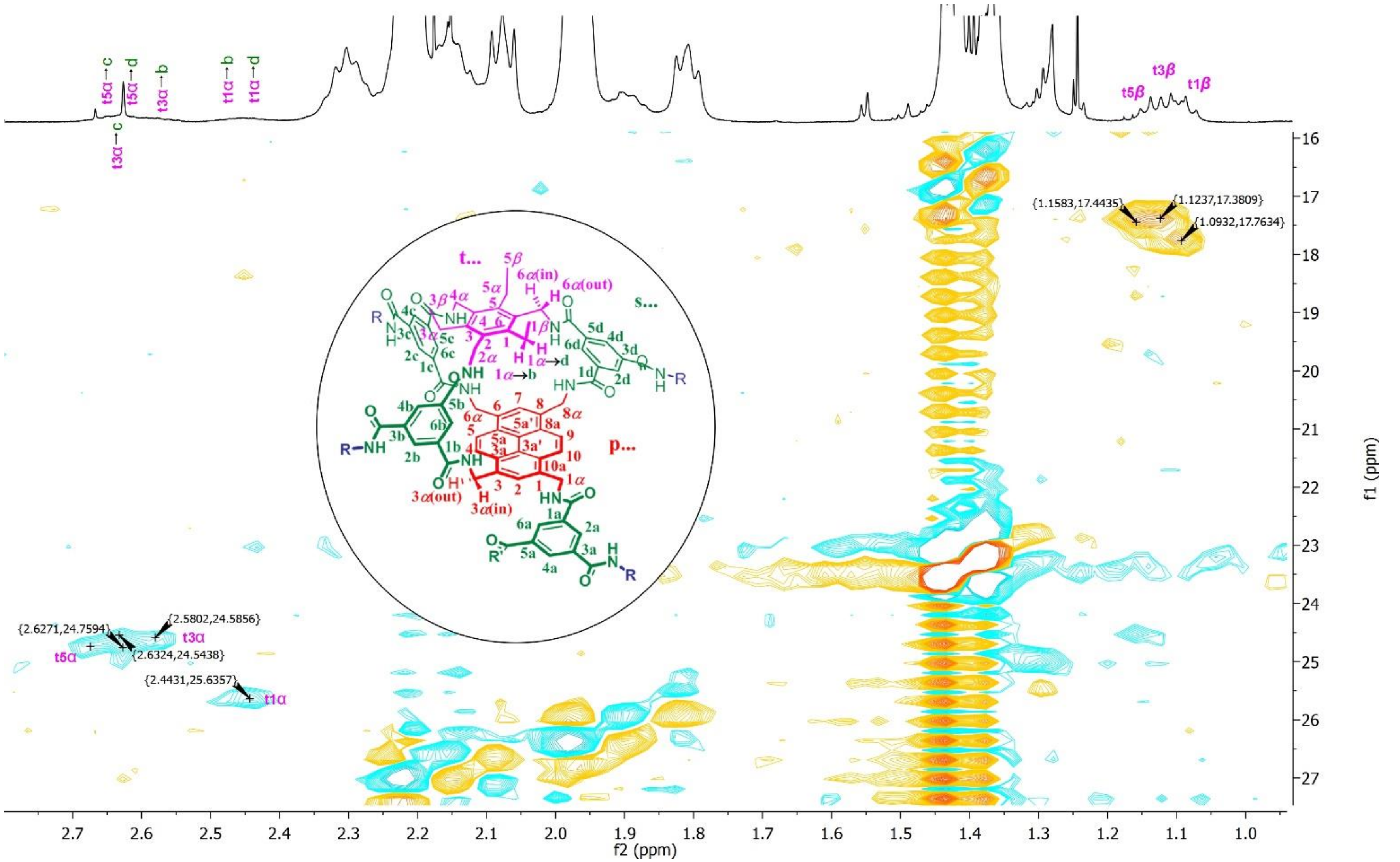

Figure S12. Partial {^{1}H-^{13}C}-HSQC (600 MHz) in CD_3OD showing aliphatic signals of protected receptors (±)-**7**.

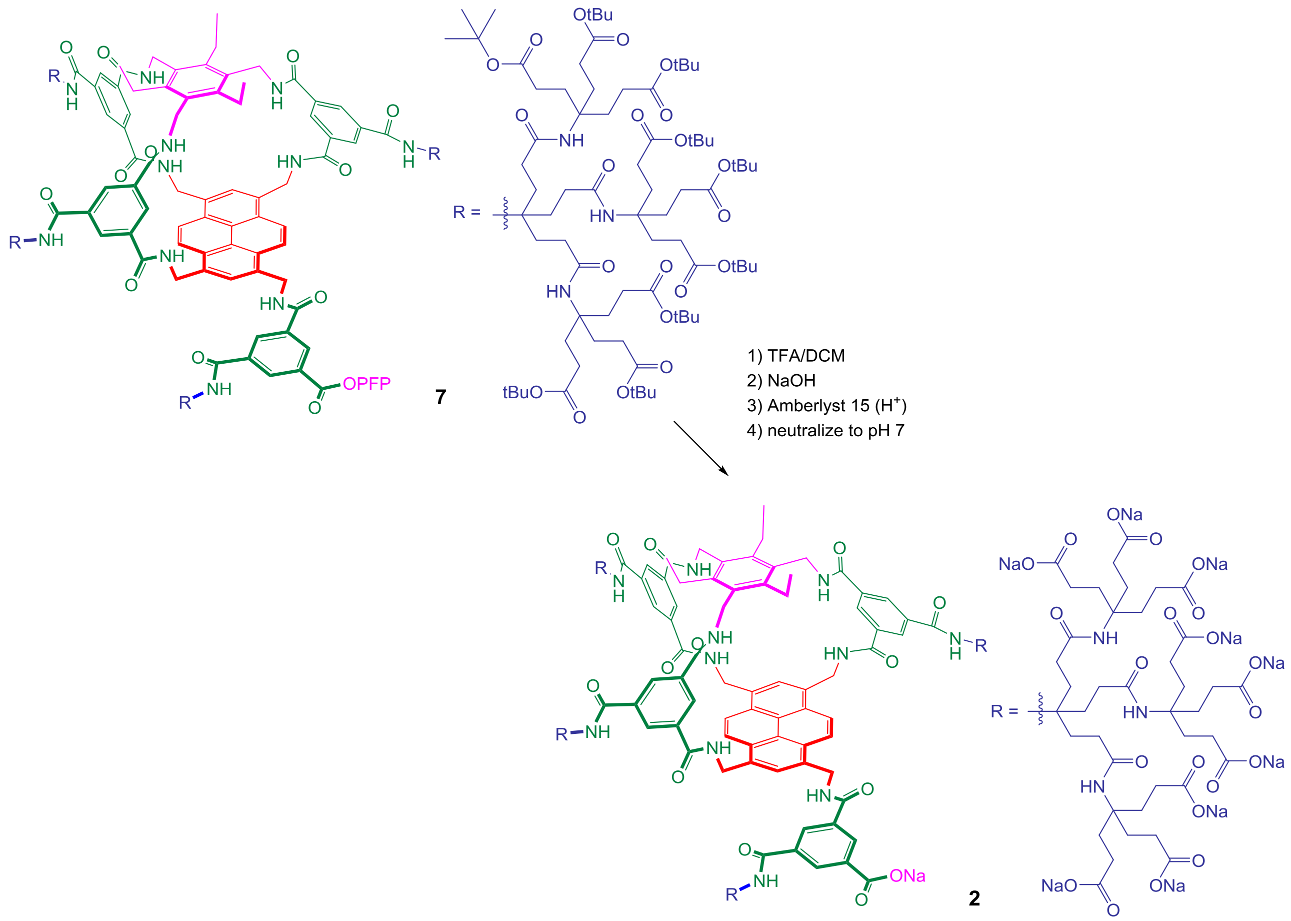

Deprotected receptors (±)-**2.** A 10 mL round-bottom flask was charged with a magnetic stirrer bar, protected receptors (±)-**7** (17 mg, 2.38 μmol) and dichloromethane (2.5 mL). The solution was cooled to 0 °C, and trifluoroacetic acid (2 mL) was added in a dropwise manner. The solution was allowed to warm to room temperature and left stirring for 24 hours, after which the solvents were removed by applying a gentle flow of N_2. H_2O (2 mL) was then added to the residue, which was sonicated and freeze-dried. To the remaining solid, H_2O (2 mL) was added. Aqueous NaOH (0.2 g/mL) was added to the suspension until pH = 14. Then, Amberlyst 15 (Hydrogen form) resin was added until the pH reached below 7 and a solid was formed. This suspension was then neutralized with NaOH to pH = 7 (resulting in a clear solution), filtered through a 0.2 μM filter and freeze-dried, affording receptors (±)-**2** (13.7 mg, 2.38 µmol) as a brown solid in quantitative yield. The products were identified by 1 dimensional ^{1}H and ^{19}F-NMR as well as 2 dimensional {^{1}H-^{1}H}-NOESY, {^{1}H-^{1}H}-COSY and {^{1}H-^{1}H}-TOCSY as is detailed below and in Figure S13 - Figure S23. A dilution study of receptors (±)-**2** in the range 1 mM – 125 μM revealed that these molecules are monomeric at concentrations < 0.5 mM (see Figure S24). ^{1}H NMR (D_2O, 600 MHz): δ = 0.05 (m, 1H, t1β), 0.98 (m, 1H, t5β), 0.99 (m, 1H, tβb), 1.53 – 2.25 (m, 192H, d2+d3+d7+d8), 2.40 (m, 1H, t1α→b), 2.54 (m, 1H, t1α→d), 2.50 (m, 1H, t3α→c), 2.52 (m, 1H, t3α→b), 2.52 (m, 1H, t5α→d), 2.54 (m, 1H, t5α→c), 4.26 (m, 1H, t2α(in)), 4.28 (m, 1H, t6α(out)), 4.49 (m, 1H, t4α(in)), 4.55 (m, 1H, t4α(out)), 4.57 (m, 1H, t6α(in)), 4.60 (m, 1H, t2α(out)), 4.76 (d, 1H, p8α(in)),

4.73 (d, 1H, p3α(in)), 4.91 (d, 1H, *J* = 15.8 Hz, p1α(in)), 5.50 (d, 1H, *J* = 14 Hz, p6α(out)), 5.57 (d, 1H, *J* = 15.7 Hz, p1α(out)), 5.66 (d, 1H, *J* = 15.1 Hz, p3α(out)), 5.72 (d, 1H, *J* = 13.8 Hz, p8α(out)), 7.68 (s, 1H, s6b), 7.73 (s, 1H, s6d), 7.93 (s, 1H, s6c), 8.07 (s, 1H, s6a), 8.20 (s, 1H, s4a), 8.21 (s, 1H, p2), 8.23 (s, 1H, p7), 8.29 (s, 1H, s2b), 8.29 (s, 1H, s2d), 8.29 (s, 1H, s4d), 8.31 (s, 1H, s6c), 8.36 (s, 1H, s4b), 8.36 (d, 1H, p9), 8.36 (s, 1H, s4c), 8.45 (d, 1H, *J* = 10.7 Hz, p10), 8.47 (d, 1H, *J* = 11.2 Hz, p5), 8.55 (d, 1H, *J* = 9.4 Hz, p4), 8.87 (s, 1H, s2a) p.p.m.; ^{19}F NMR (D_2O, 470 MHz): silent. MALDI-TOF (from 5% v/v formic acid in water): *m/z* calculated for $C_{231}H_{299}N_{23}O_{97}Na$ [M (COOH-form) + Na^+]$^+$; 4972.9, found 4972.6; $C_{231}H_{301}N_{23}O_{98}Na$ [M (COOH-form) + Na^+ + H_2O]$^+$; 4991.0, found 4990.6; $C_{232}H_{301}N_{23}O_{99}Na$ [M (COOH-form) + Na^+ + HCOOH]$^+$; 5019.0, found 5018.8.

Separation of (±)-**2** was attempted by Chiral Technologies Europe on Chiralpak analytical columns type IA, IB, IF and ZWIX, all 250 mm long and with 3 mm internal diameter. The eluents tested were acetonitrile/water (from 9:1 to 1:9), methanol/water ((from 9:1 to 1:9), and 0.2% formic acid in methanol/water (7:3 and 6:4). The analyte was injected (10 µL of 0.1 mM solution) in acetonitrile/water (1:1) and the eluent was running at 1 mL/min. Detection was achieved using a UV-VIS spectrophotometer. One single peak always eluted either at the front or at several minutes, depending on the eluent.

In another attempt, GlcNAc separopore® 4B agarose resin (Bioworld)[5] was packed into a 1 ml borosilicate FPLC column, and the column was fitted on an AKTA Purifier system (GE Biosciences) at ambient temperature. Initially the receptor mixture was diluted to 0.1 mg/ml in H_2O and 1 ml was loaded onto the column and eluted with an isocratic flow rate of 1 ml/min for 60-180 min. The eluent was monitored at 215 and 280 nm. As all the material eluted in the void volume of the column, the run was repeated (i) with incubation of (±)-**2** on the column for 30 minutes before elution, and (ii) using a lower flow rate of 0.1 ml/ml. Neither measure yielded any change. The attempted separation was repeated in 0.1% formic acid/H_2O, with isocratic elution at 0.1 ml/min, but again all the material appeared in the void volume. GlcNAc separopore® 4B is used to separate GlcNAc-binding lectins such as Wheat Germ Agglutinin, which binds to GlcNAc more weakly than one enantiomer of **2**. It thus seems that the presentation of the GlcNAc units on this column is incompatible with binding to the synthetic lectin.

[5] http://www.bio-world.com/productinfo/2_18_162_669/1315/N-Acetyl-glucosamine-GlcNAc-Separopore-Agarose-B-CL.html

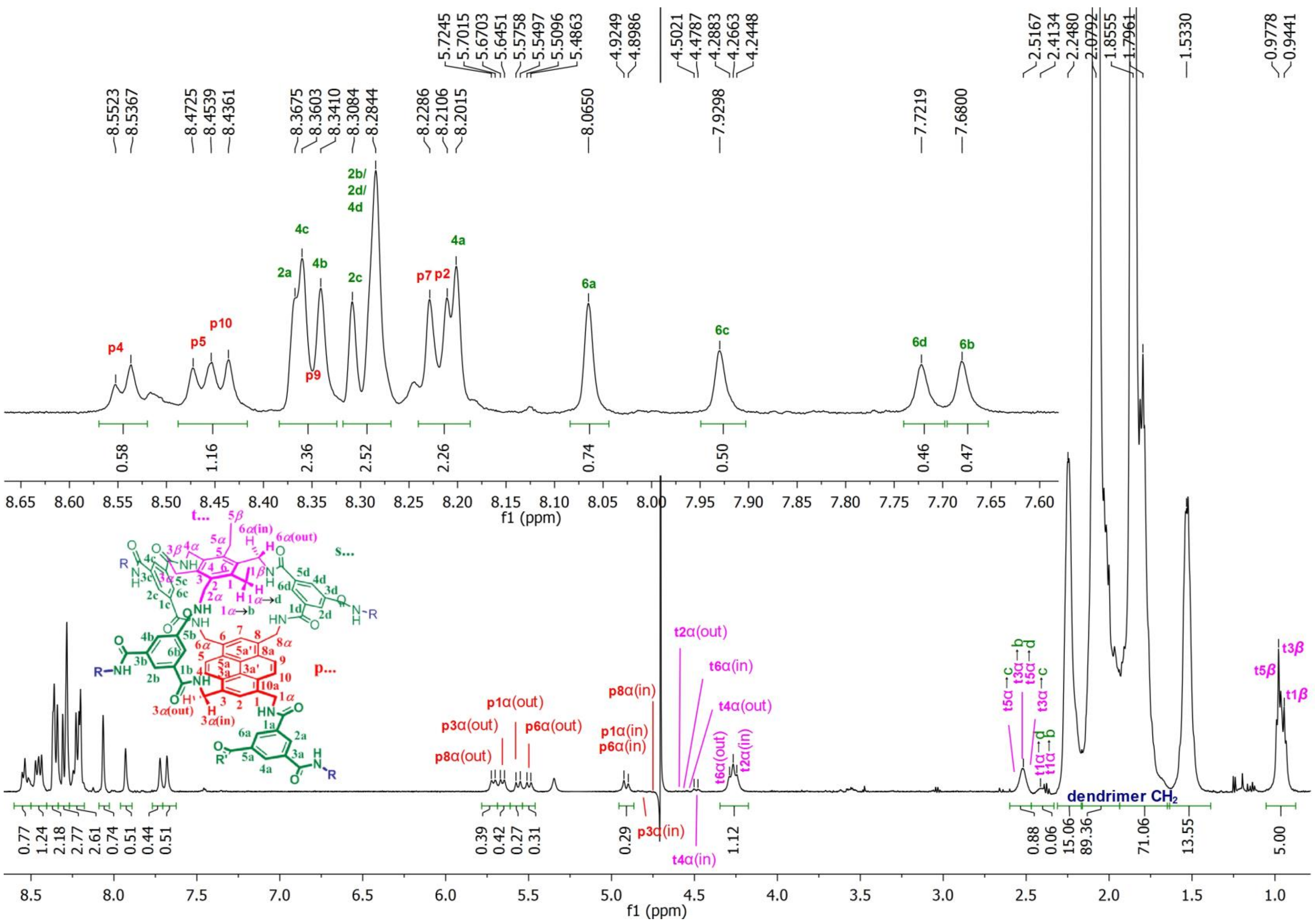

Figure S13. ^{1}H-NMR (600 MHz) spectrum of receptors (±)-**2**, at 1 mM concentration in D_2O.

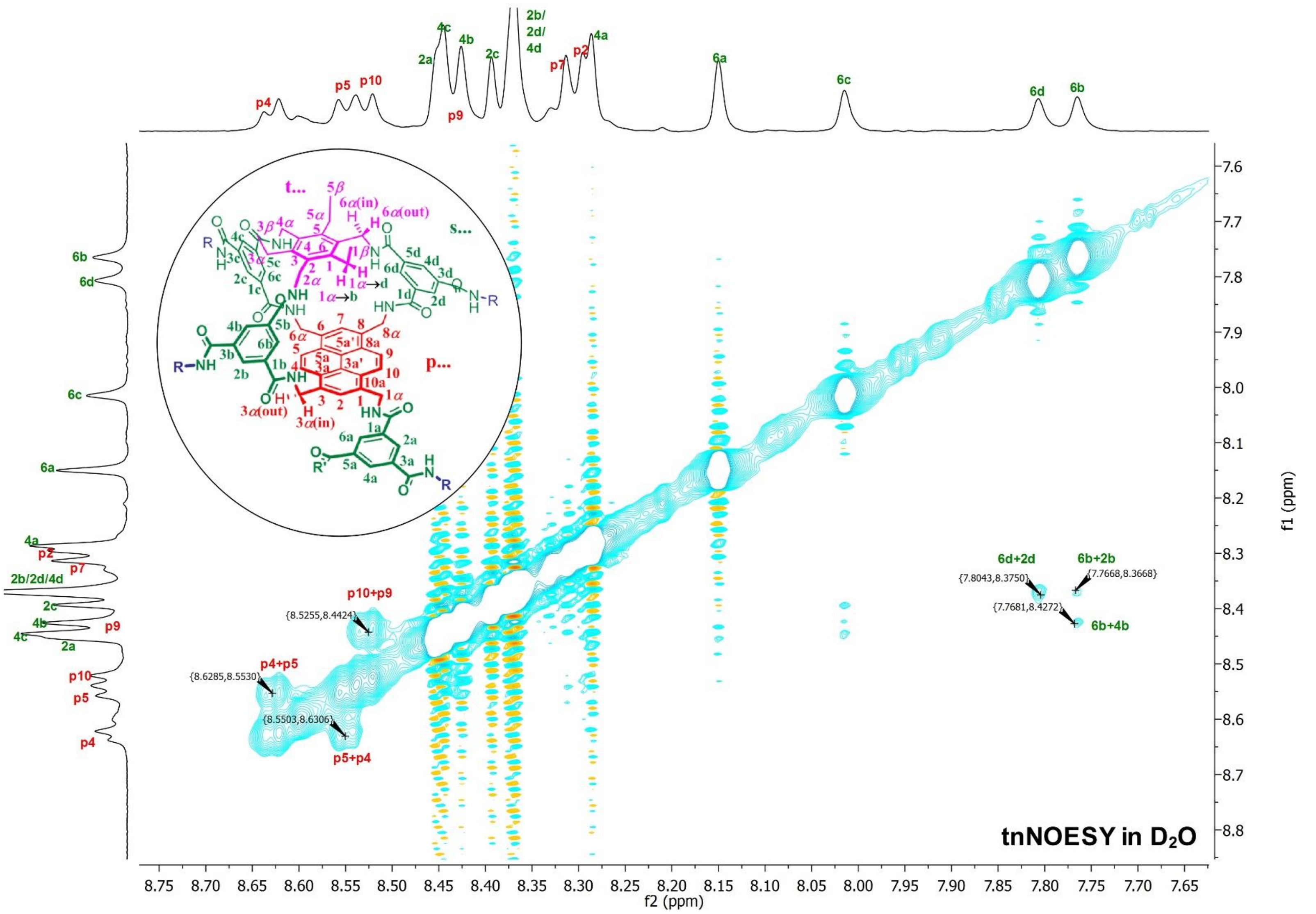

Figure S14. Partial {^{1}H-^{1}H}-NOESY (600 MHz) spectrum of receptors (±)-**2**, at 1 mM concentration in D$_2$O. NB: chemical shifts offset by 0.09 p.p.m.

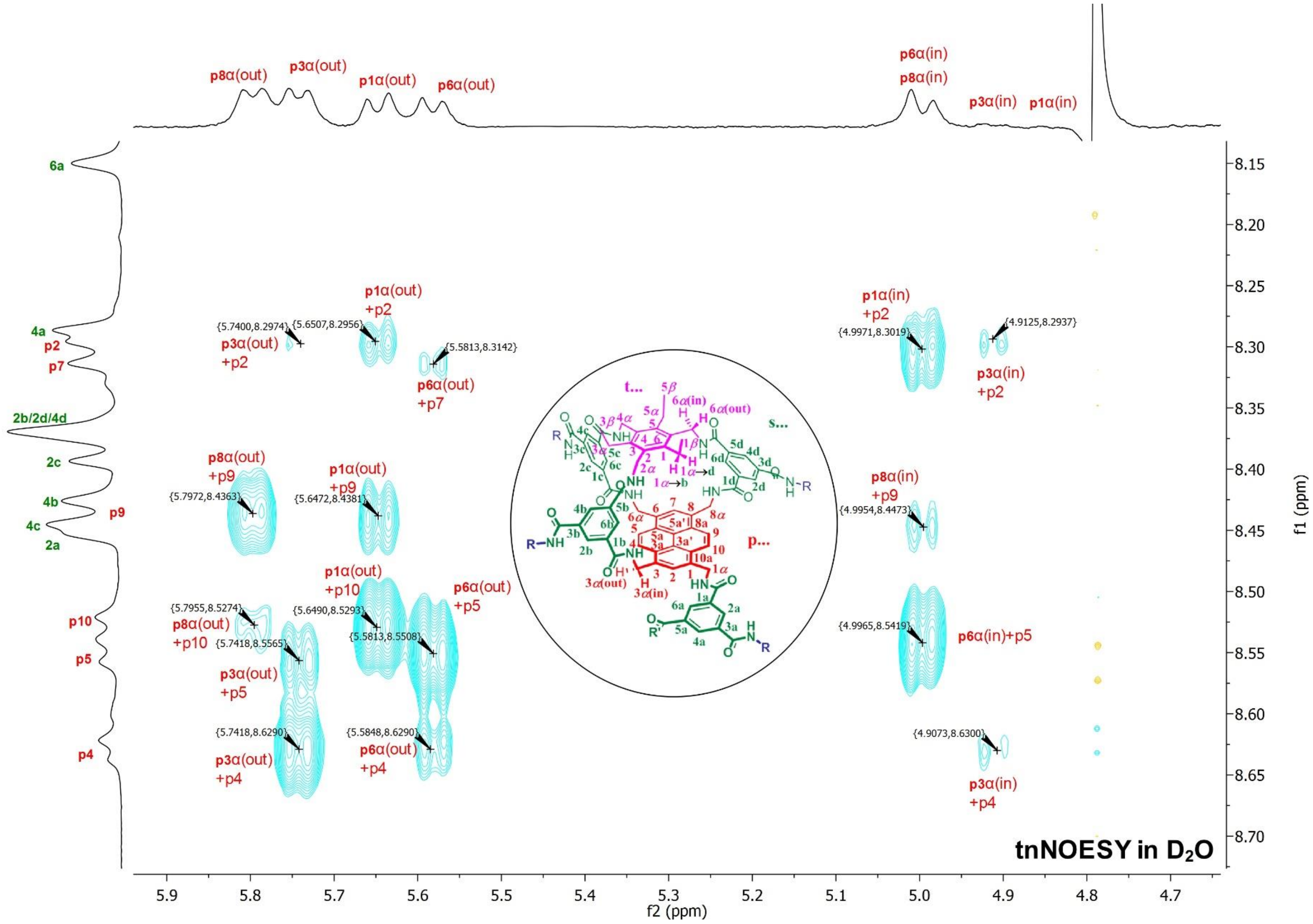

Figure S15. Partial {^{1}H-^{1}H}-NOESY (600 MHz)spectrum of receptors (±)-**2**, at 1 mM concentration in D_2O. NB: chemical shifts offset by 0.09 p.p.m.

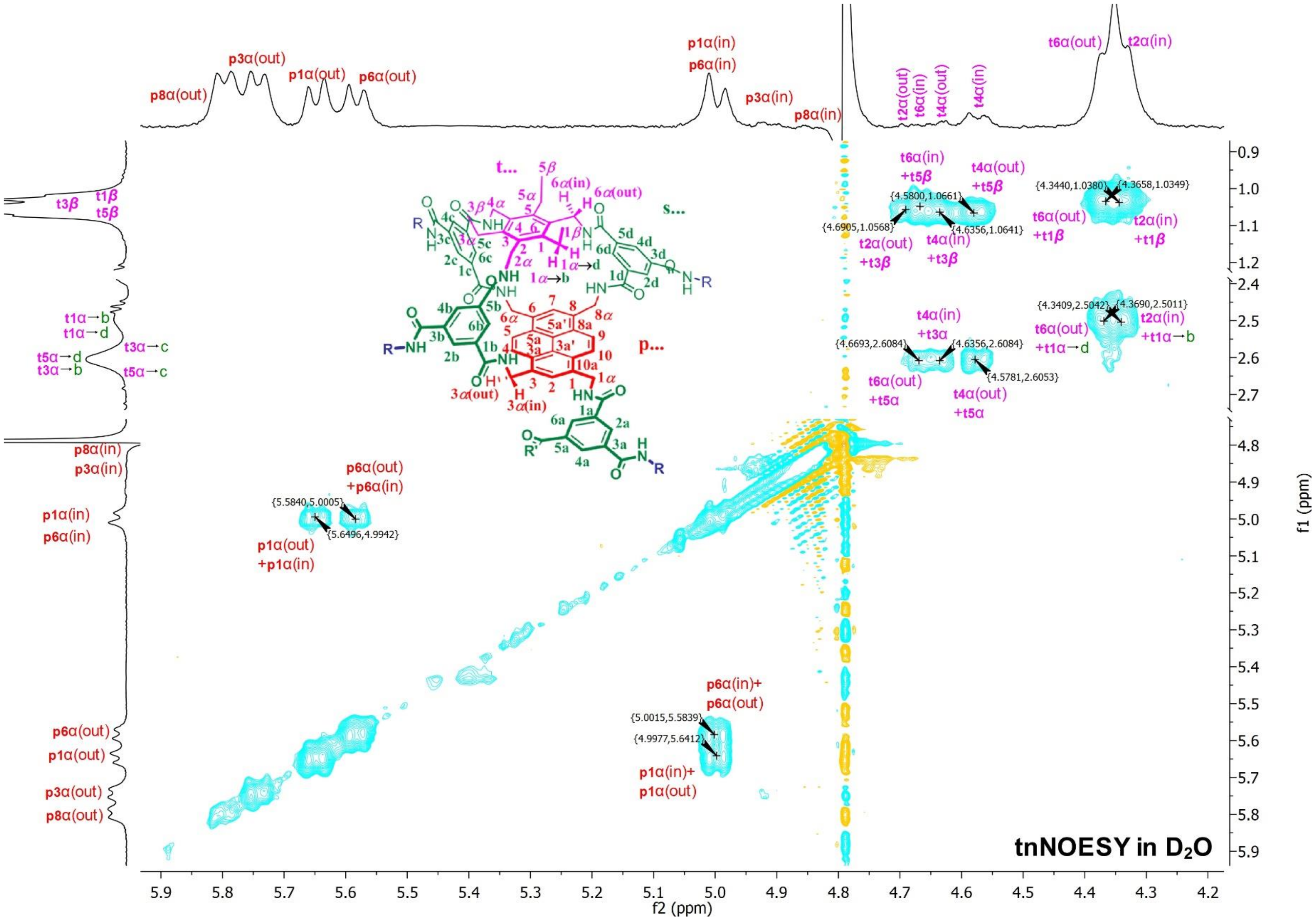

Figure S16. Partial {^{1}H-^{1}H}-NOESY (600 MHz) spectrum of receptors (±)-**2**, at 1 mM concentration in D_2O. NB: chemical shifts offset by 0.09 p.p.m.

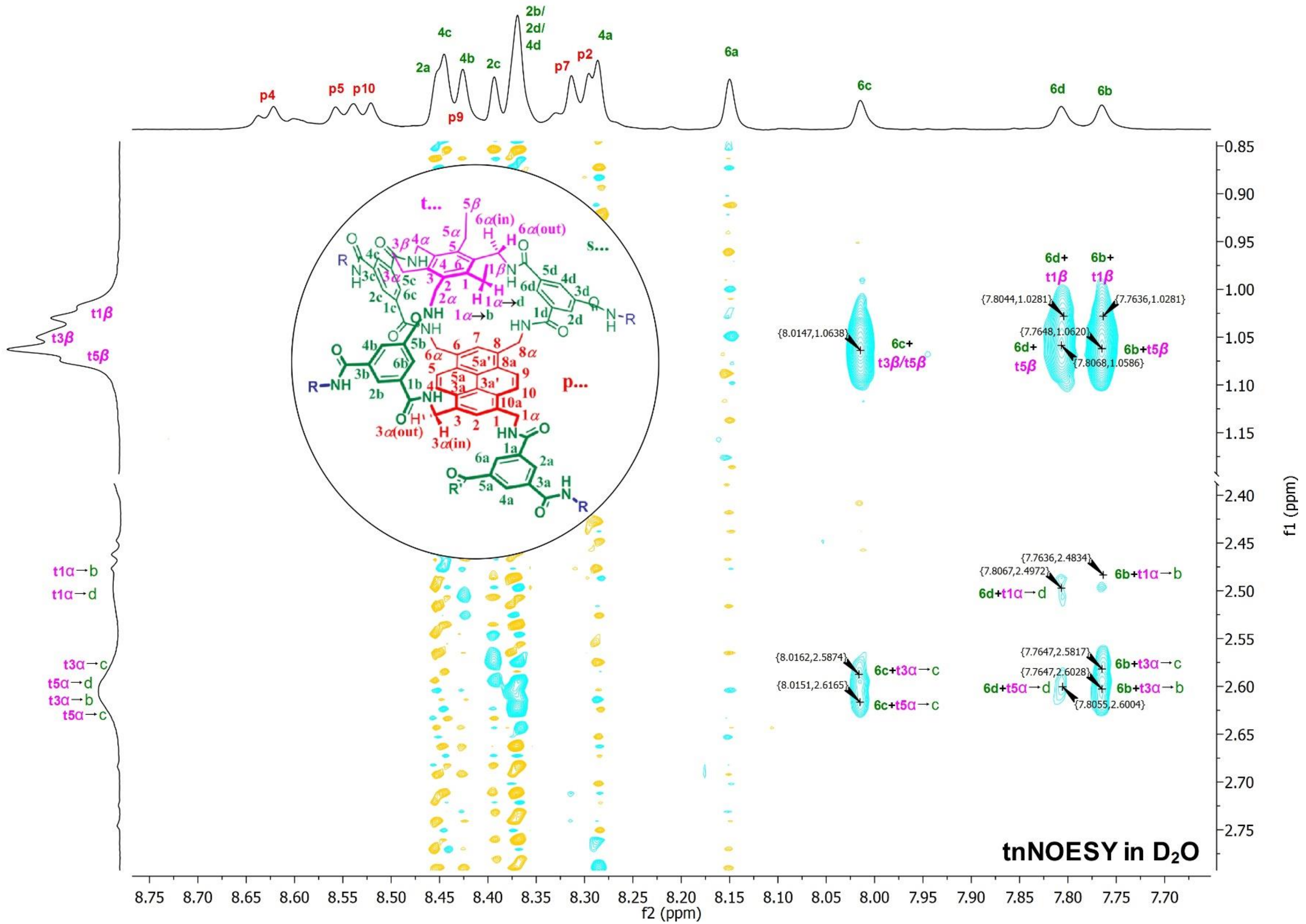

Figure S17. Partial {^{1}H-^{1}H}-NOESY (600 MHz) spectrum of receptors (±)-**2**, at 1 mM concentration in D_2O. NB: chemical shifts offset by 0.09 p.p.m.

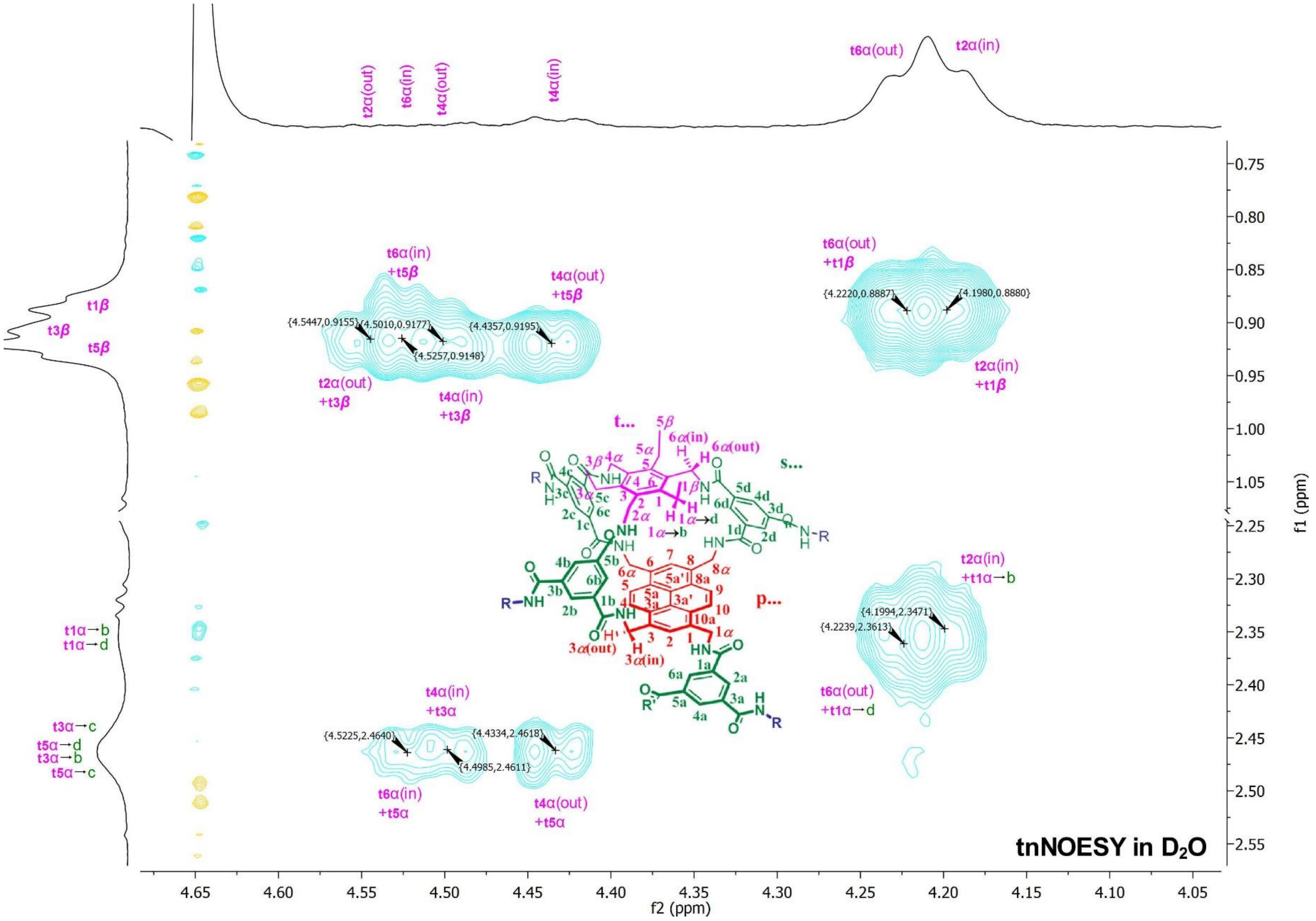

Figure S18. Partial {^{1}H-^{1}H}-NOESY (600 MHz) spectrum of receptors (±)-**2**, at 1 mM concentration in D_2O. NB: chemical shifts offset by 0.09 p.p.m.

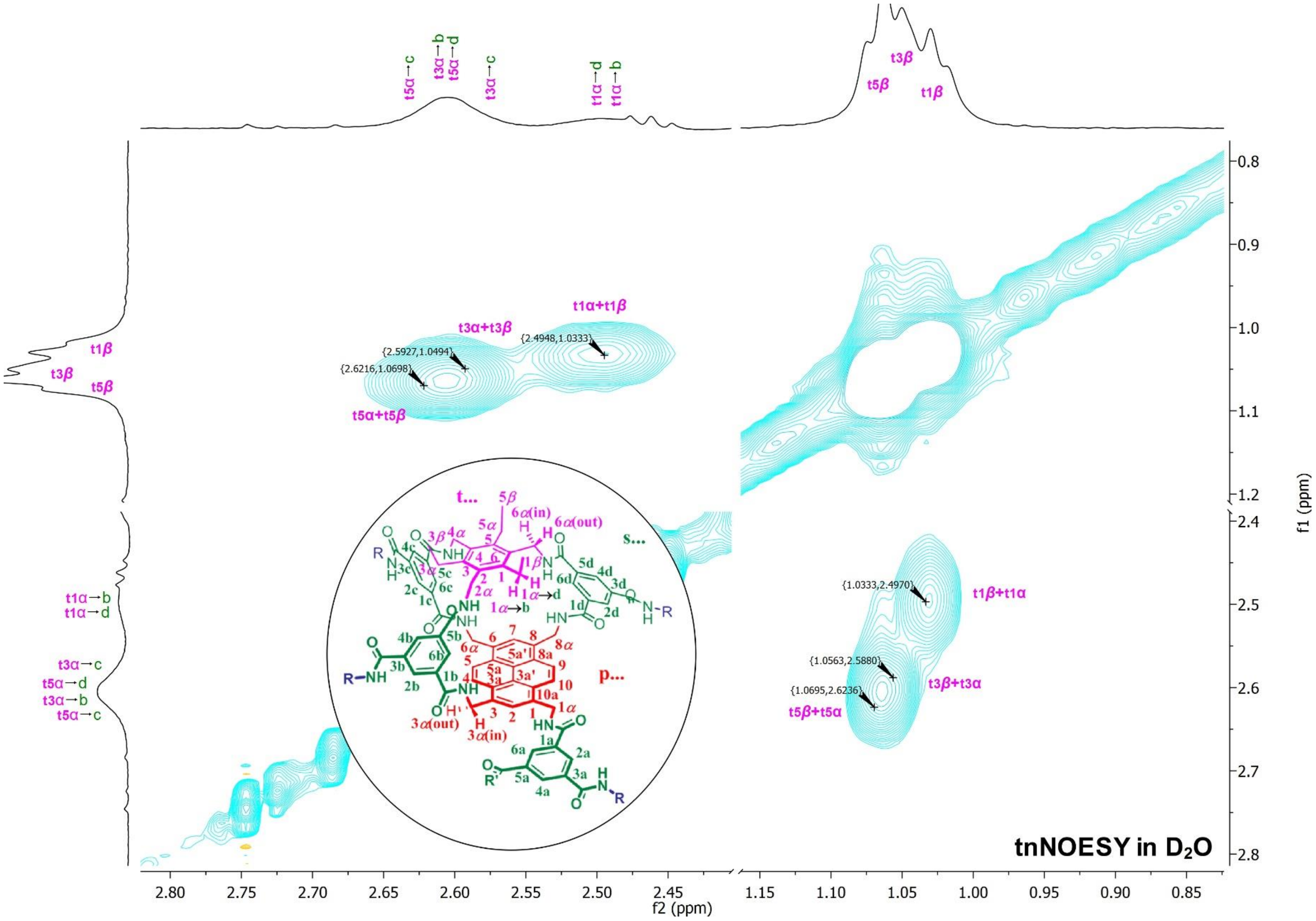

Figure S19. Partial {^{1}H-^{1}H}-NOESY (600 MHz) spectrum of receptors (±)-**2**, at 1 mM concentration in D_2O. NB: chemical shifts offset by 0.09 p.p.m..

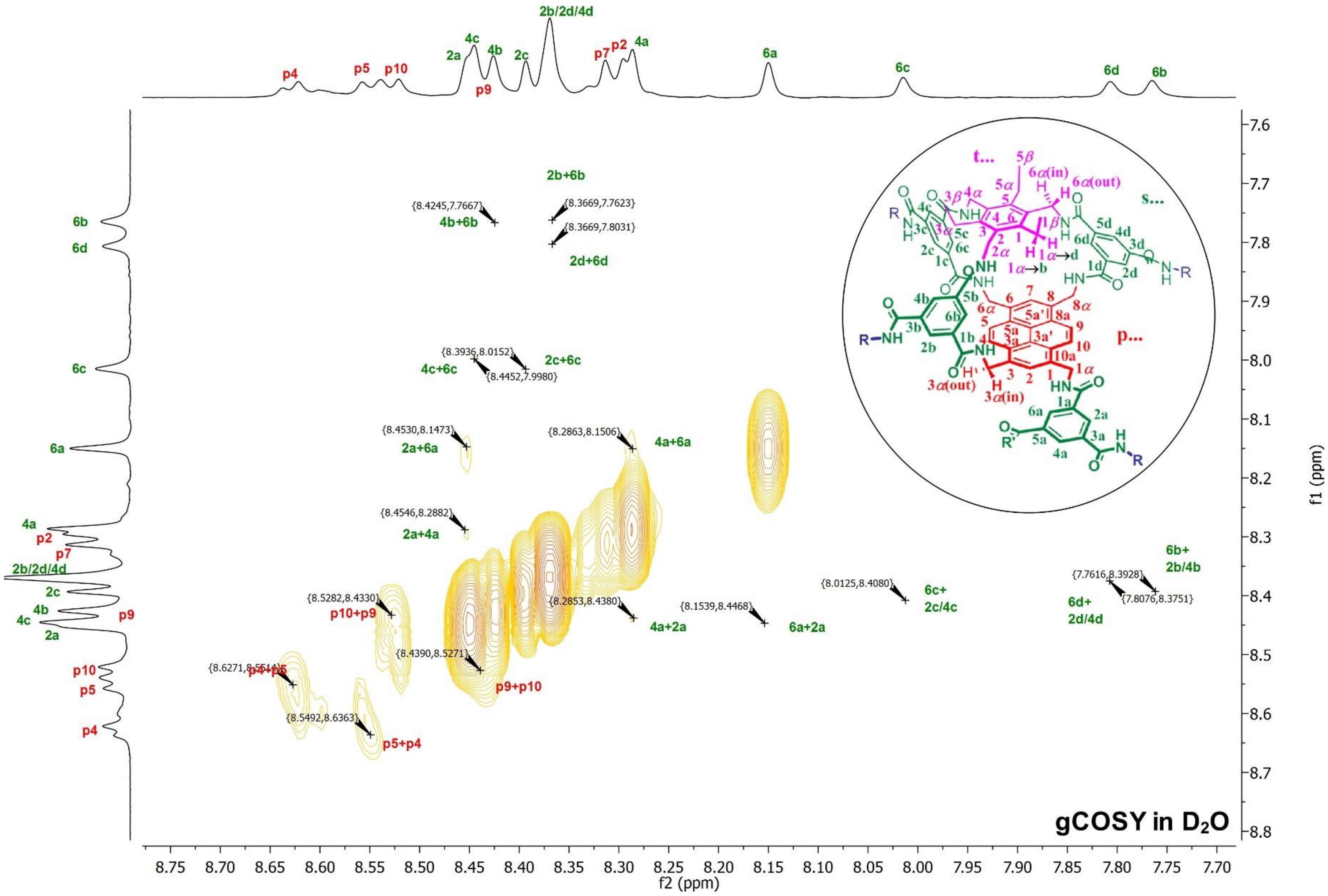

Figure S20. Partial {^{1}H-^{1}H}-COSY (600 MHz) spectrum of receptors (±)-**2**, at 1 mM concentration in D_2O. NB: chemical shifts offset by 0.09 p.p.m.

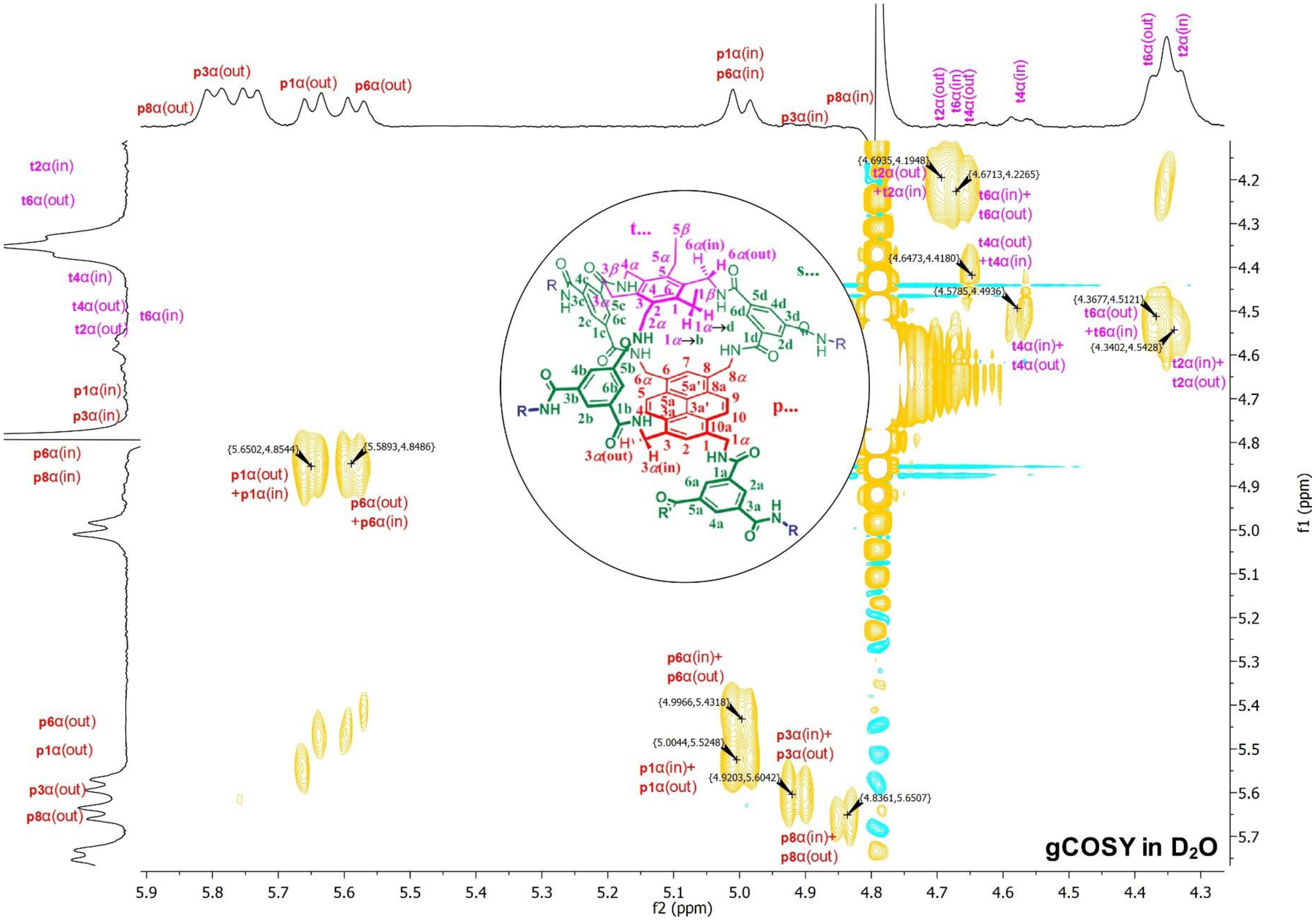

Figure S21. Partial {^{1}H-^{1}H}-COSY (600 MHz) spectrum of receptors (±)-**2**, at 1 mM concentration in D_2O. NB: chemical shifts offset by 0.09 p.p.m.

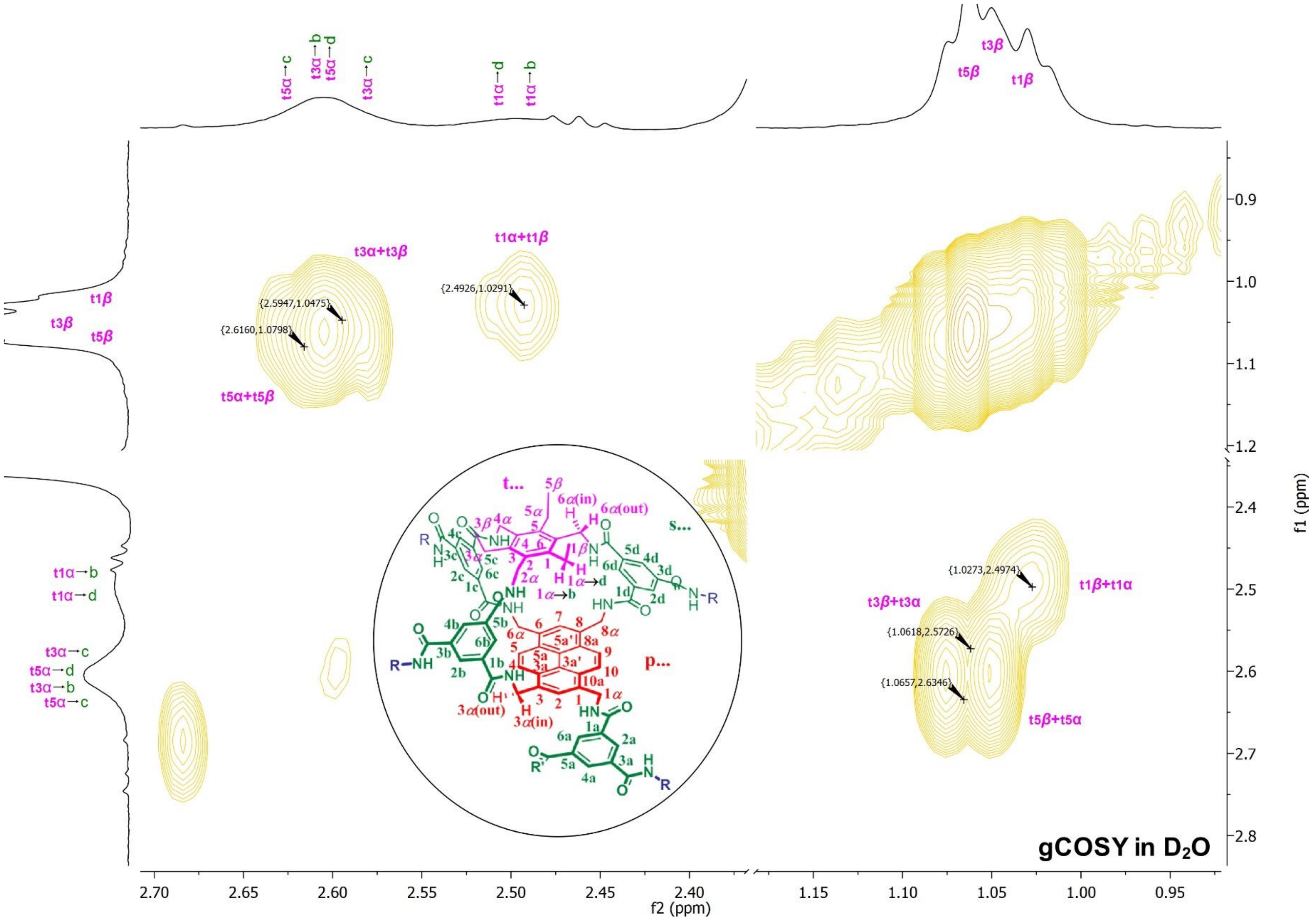

Figure S22. Partial {^1H-^1H}-COSY (600 MHz) spectrum of receptors (±)-**2**, at 1 mM concentration in D_2O. NB: chemical shifts offset by 0.09 p.p.m..

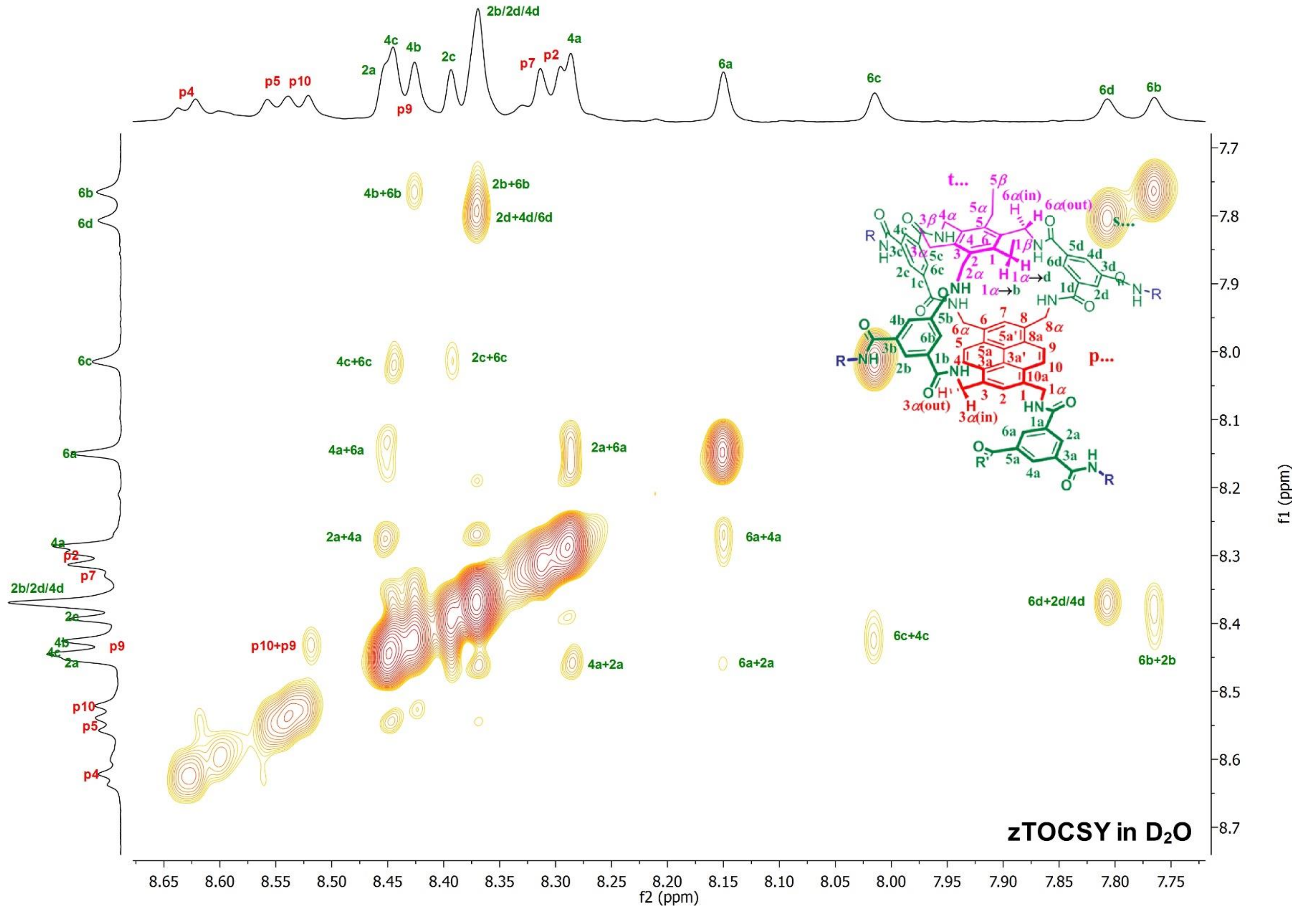

Figure S23. Partial {¹H-¹H}-TOCSY (600 MHz) spectrum of receptors (±)-**2**, at 1 mM concentration in D_2O. NB: chemical shifts offset by 0.09 p.p.m.

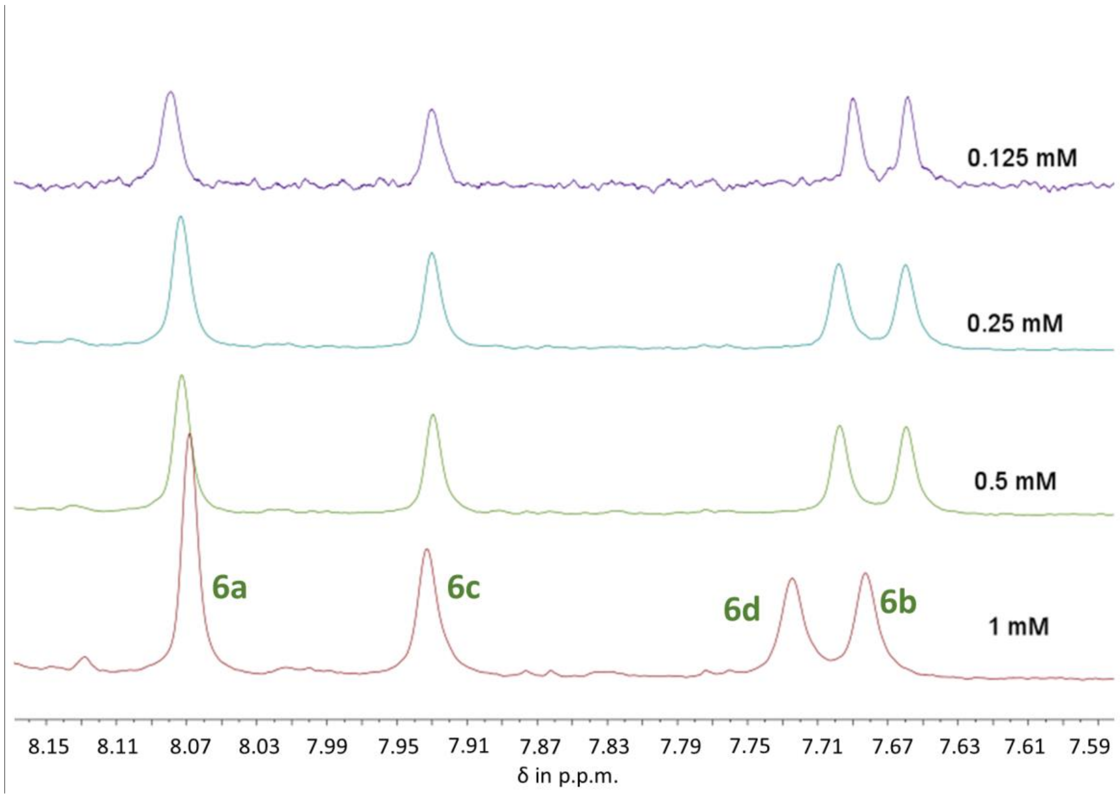

Figure S24. ^{1}H NMR (600 MHz) dilution study of receptors (±)-**2** in D_2O in the range 1 mM – 125 μM, showing the 'inwards-facing' aromatic protons (6). The marginal change from 1 mM→0.5 mM and lack of any change below 0.5 mM implies that (±)-**2** are monomeric below 0.5 mM.

2. ^{1}H NMR titrations

NMR spectra were recorded at 298 K and 600 MHz on a Varian VNMRS 600 equipped with a cryogenically cooled ^{1}H-observe triple resonance probe. Chemical shifts (δ) are reported in parts per million (p.p.m.). A solution of receptors (±)-**2** in D_2O (99.9% -D, typically 0.15 mM of each receptor) was prepared and typically 400 μL was transferred into a new or thoroughly cleaned and dried NMR tube. This same receptor solution was then used to prepare a stock solution of carbohydrate, ensuring that the concentration of (±)-**2** did not change during the subsequent titration. In the case of reducing sugars, the stock solution was left overnight so that α and β forms could equilibrate. Aliquots of increasing volume were added from the stock solution into the NMR tube and, after thorough mixing, the ^{1}H-NMR spectrum was recorded. The first addition typically was 0.5 μL; subsequent additions were made using double the volume of the previous addition (i.e. 1.0, 2.0, 4.0, 8.0 μL, etc.). Signals due to receptor protons were observed to move, as expected for binding with fast exchange on the ^{1}H NMR timescale, although broadening was also observed in many cases. Where feasible, changes in chemical shift ($\Delta\delta$) were fitted to a 1:1 binding model using a non-linear least squares curve-fitting programme implemented within Excel. The programme yields binding constants K_a and limiting Δδ as output. An estimated error for K_a could be obtained from individual data points by assuming the determined K_a and δ_{HG}. The fits obtained were good in all cases (with $r \geq 0.999$), and the thus obtained K_a and Δδ were considered trustworthy. Spectra and analysis curves are shown in Figure S28 - Figure S34.

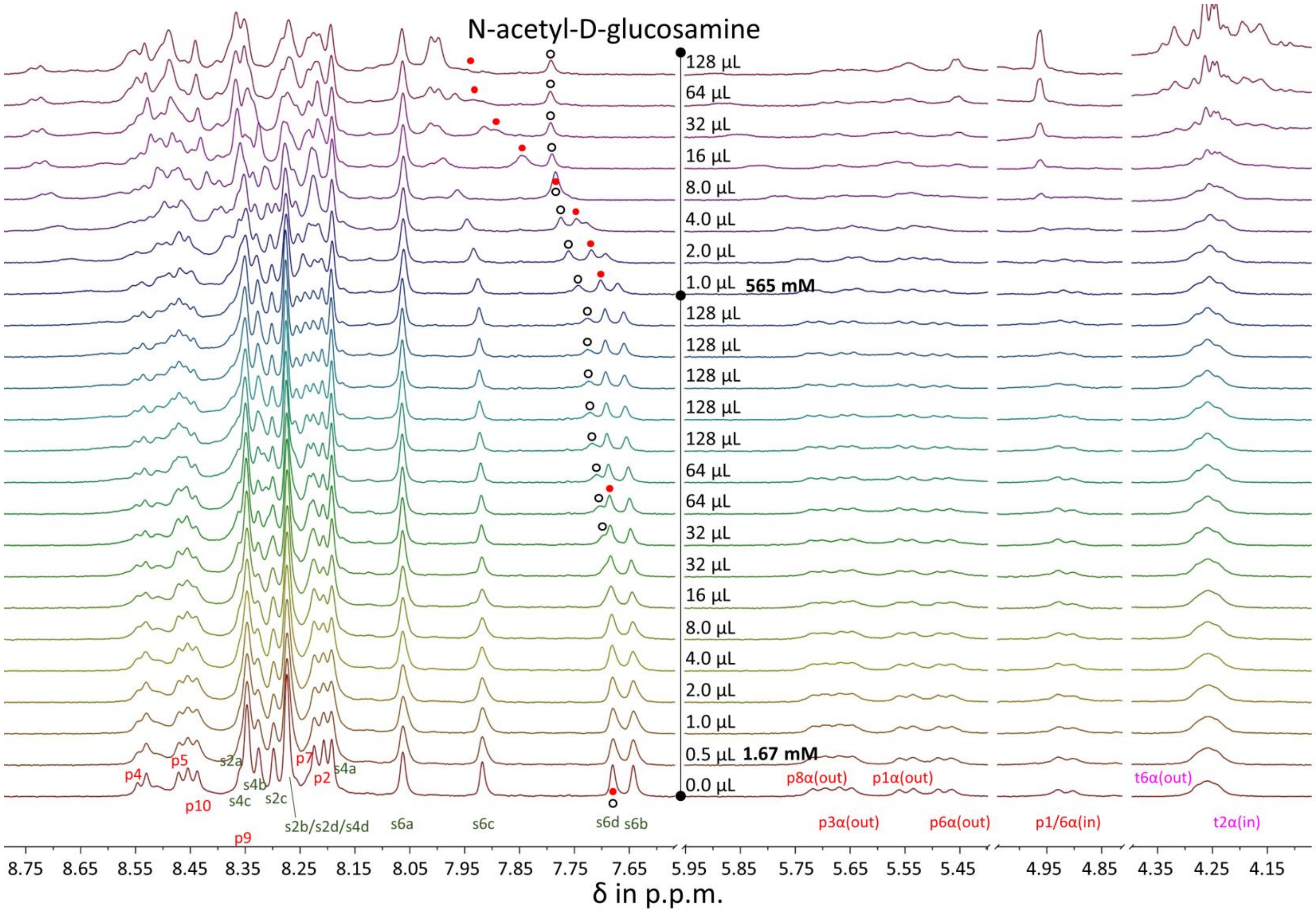

Figure S25. Partial ^{1}H NMR spectra from the titration of receptors (±)-**2** (0.15 mM each, 400 *µ*L) with *N*-acetyl-D-glucosamine **8** (0.90 mM then 501 mM). Peaks marked with empty and red circles were analysed to give binding constants for the diastereomeric complexes (see Figure S26)**.**

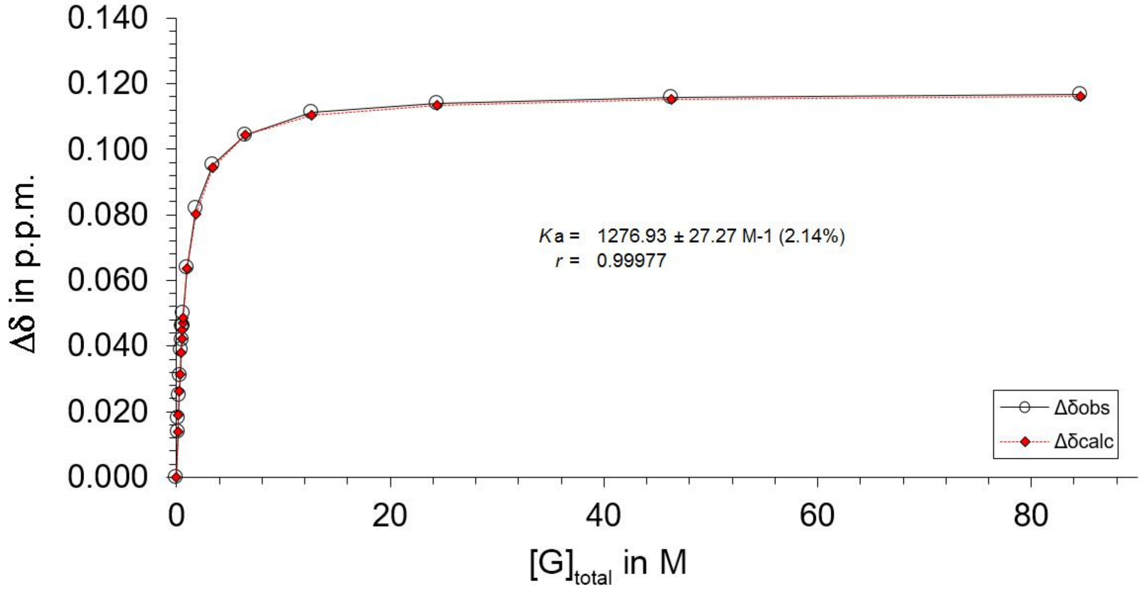

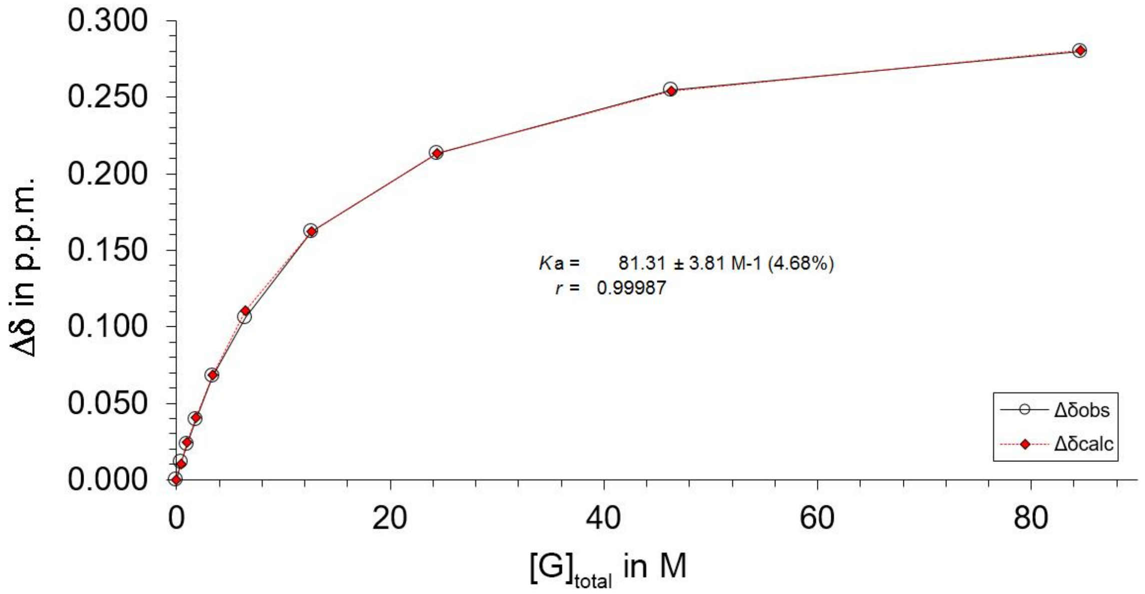

Figure S26. Data analysis for ^{1}H NMR titration of receptors (±)-**2** with *N*-acetyl-D-glucosamine **8** (see Figure S25). Top: K_a = 1277 ± 27 M^{-1} (2.14 %); *r* = 0.99977; limiting Δδ = 0.117 p.p.m. Bottom: K_a = 81.3 ± 4.9 M^{-1} (4.68 %); *r* = 0.99987; limiting Δδ = 0.331 p.p.m..

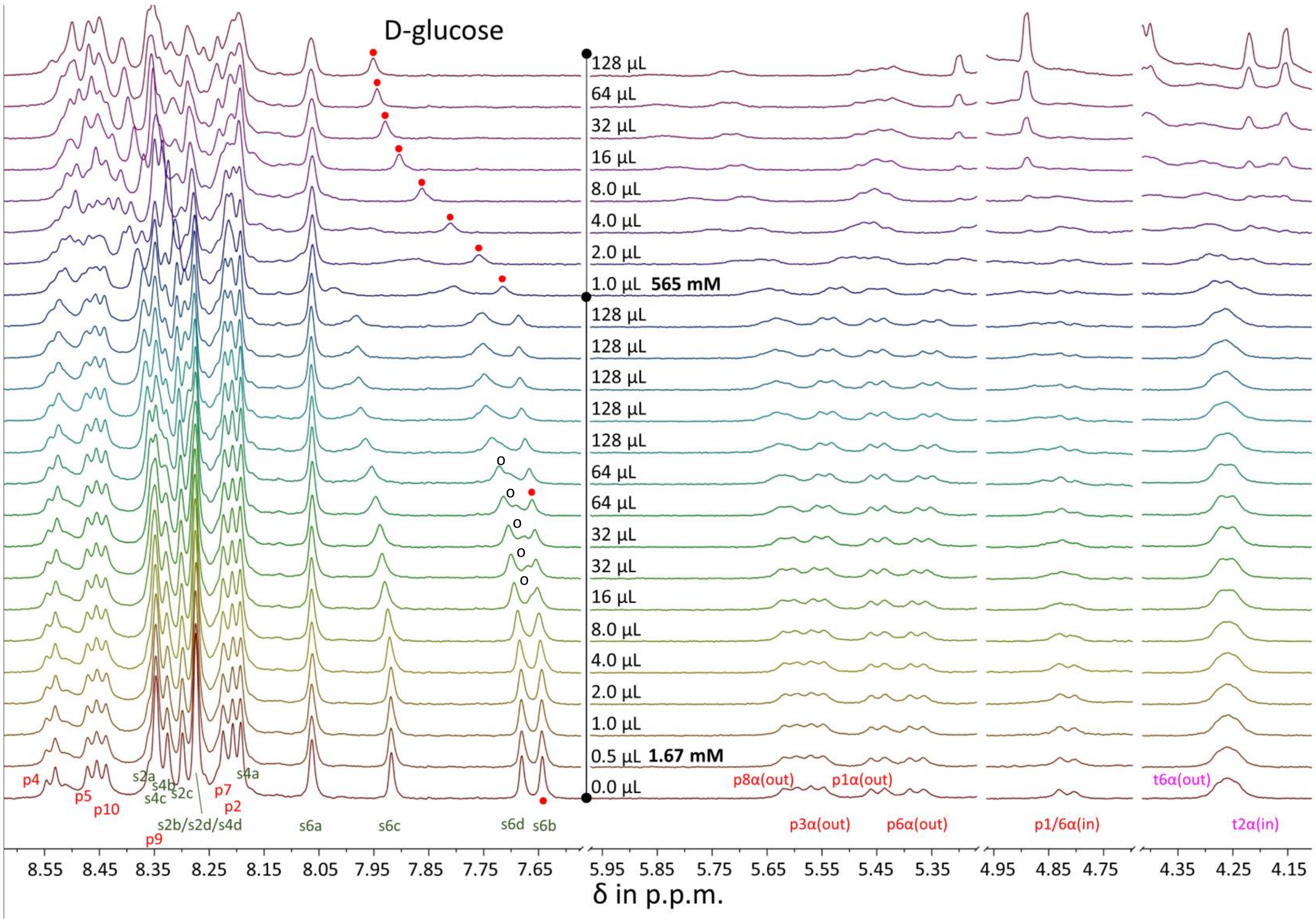

Figure S27. Partial ^{1}H NMR spectra from the titration of receptors (±)-**2** (0.15 mM each, 400 μL) with D-glucose **9** (1.67 mM then 565 mM). Peaks marked with red circles were analysed to give the binding constant of ~250 M^{-1} for one of the diastereomeric complexes (see Figure S28). Peaks marked with empty circles are assigned to the second diastereomer. Their early movement during the titration suggests a high binding constant, perhaps larger than that calculated for the first diastereomer.

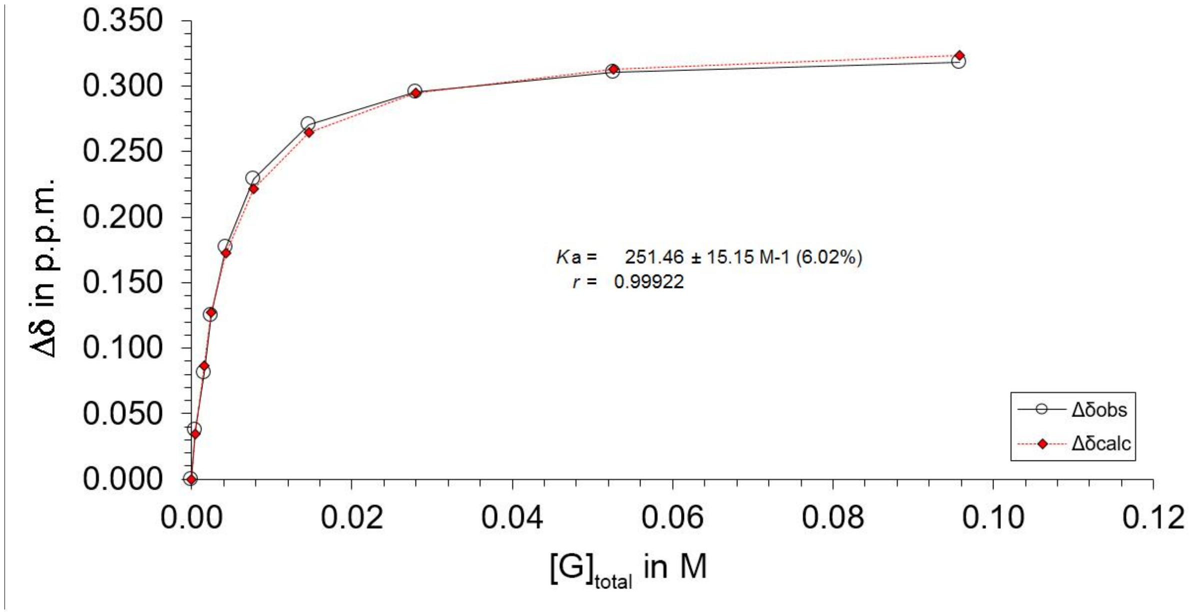

Figure S28. Data analysis for ^{1}H NMR titration of one of the diastereomers of receptors (±)-**2** with D-glucose **9** (see Figure S27) K_a = 251.46 ± 15.15 M^{-1} (6.02 %); r = 0.99922 ; limiting Δδ = 0.337 p.p.m..

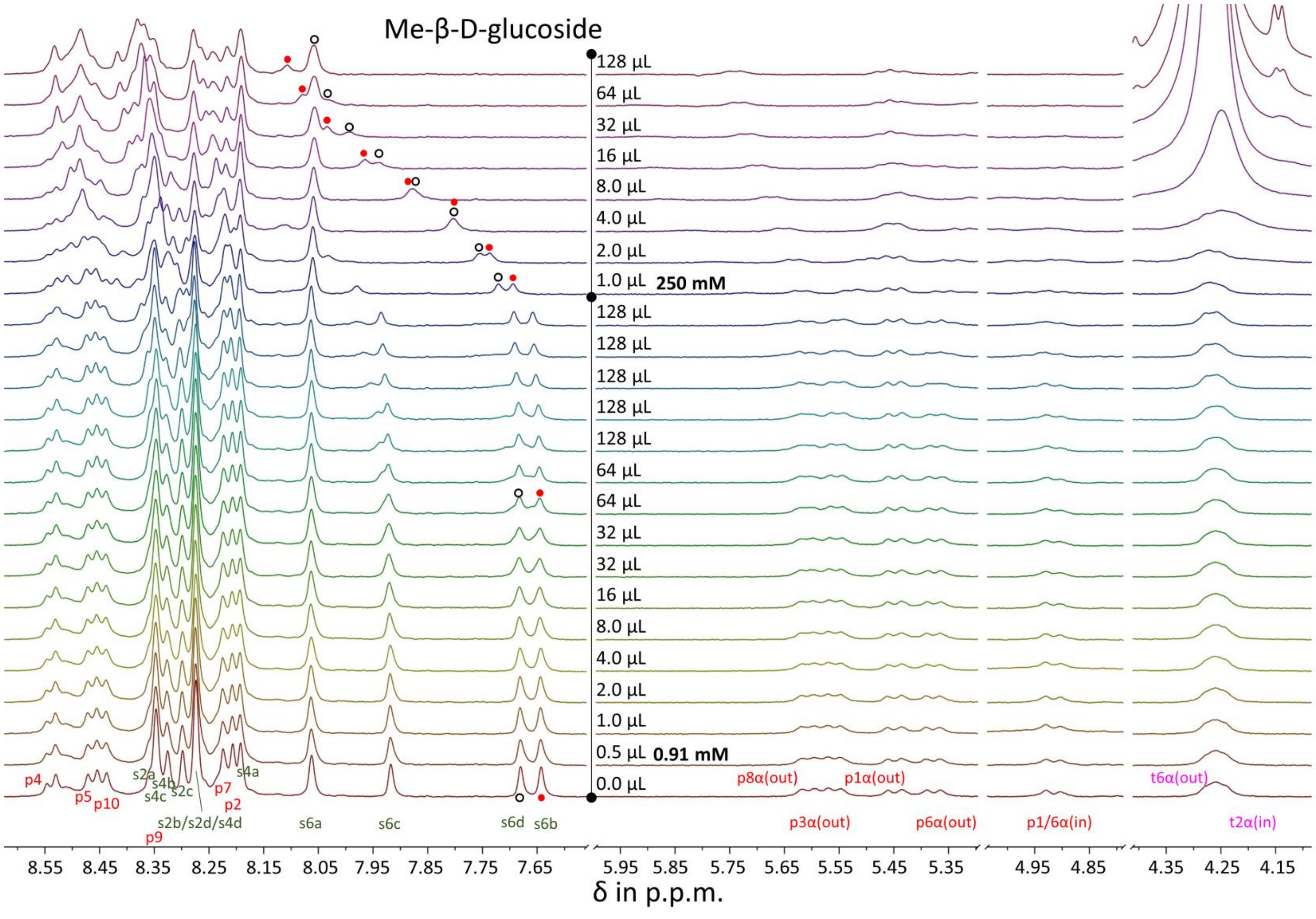

Figure S29. Partial ^{1}H NMR spectra from the titration of receptors (±)-**2** (0.15 mM each, 400 μL) with methyl-β-D-glucoside **10** (0.91 mM then 250 mM). Peaks marked with empty and red circles were analysed to give the binding constant for one of the diastereomeric complexes (see Figure S30).

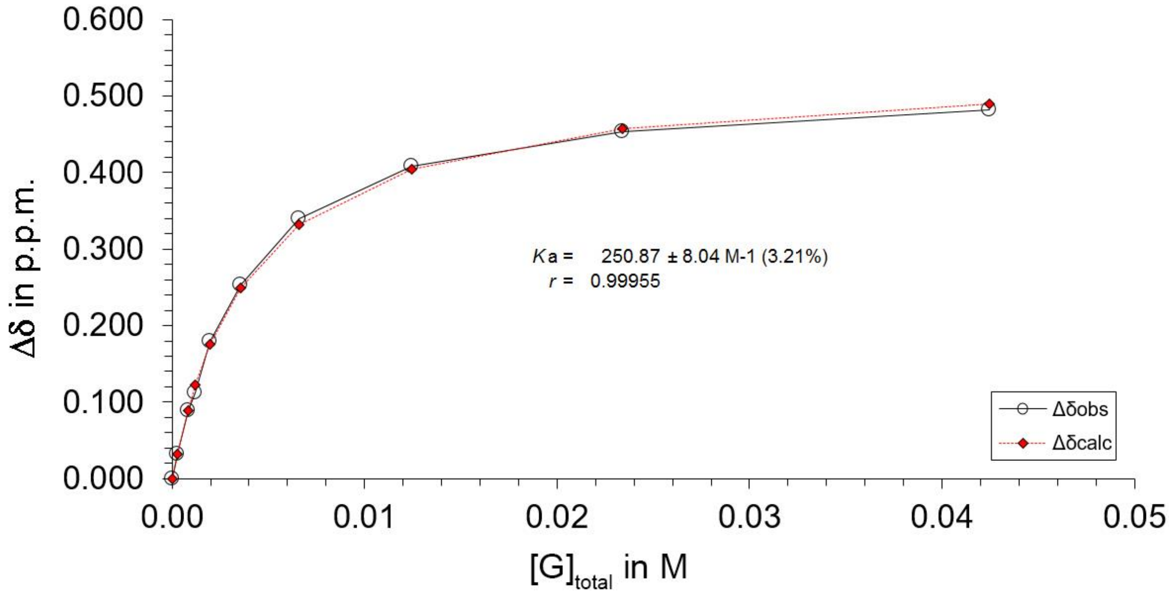

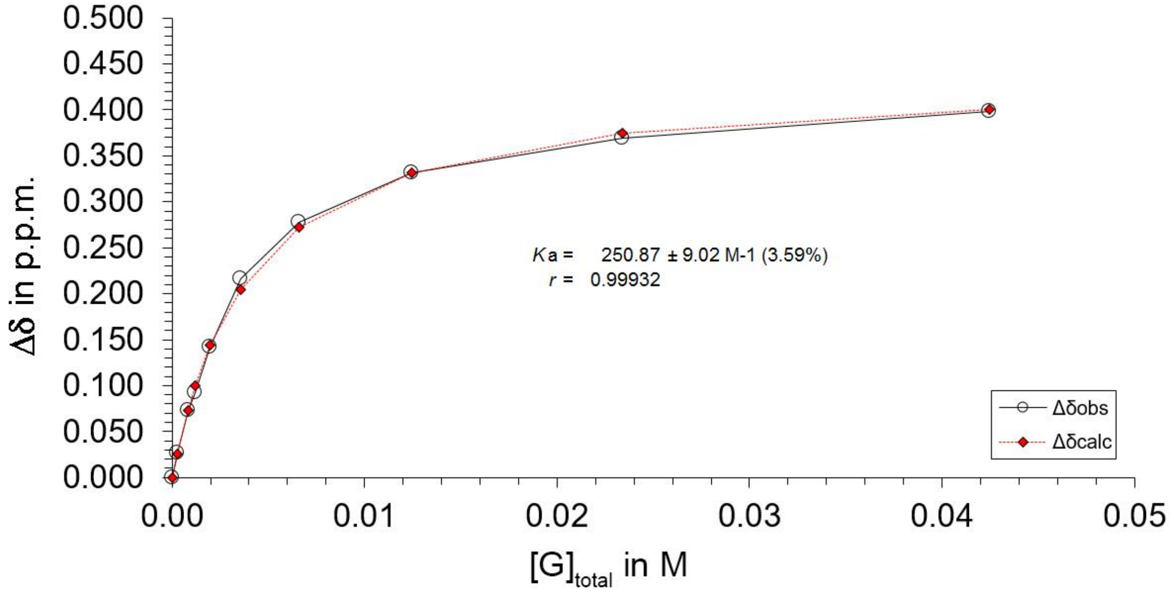

Figure S30. Data analysis for ^{1}H NMR titration of one of the enantiomers of receptors (±)-**2** with methyl-β-D-glucoside **10** (see Figure S29). Top: Peak marked with red circles in Figure S29, K_a = 250.87 ± 8.04 M^{-1} (3.21 %); *r* = 0.99955 ; limiting Δδ = 0.535 p.p.m.. Bottom: Peak marked with empty circles in Figure S29, K_a = 250.87 ± 9.02 M^{-1} (3.59 %); *r* = 0.99932 ; limiting Δδ = 0.439 p.p.m..

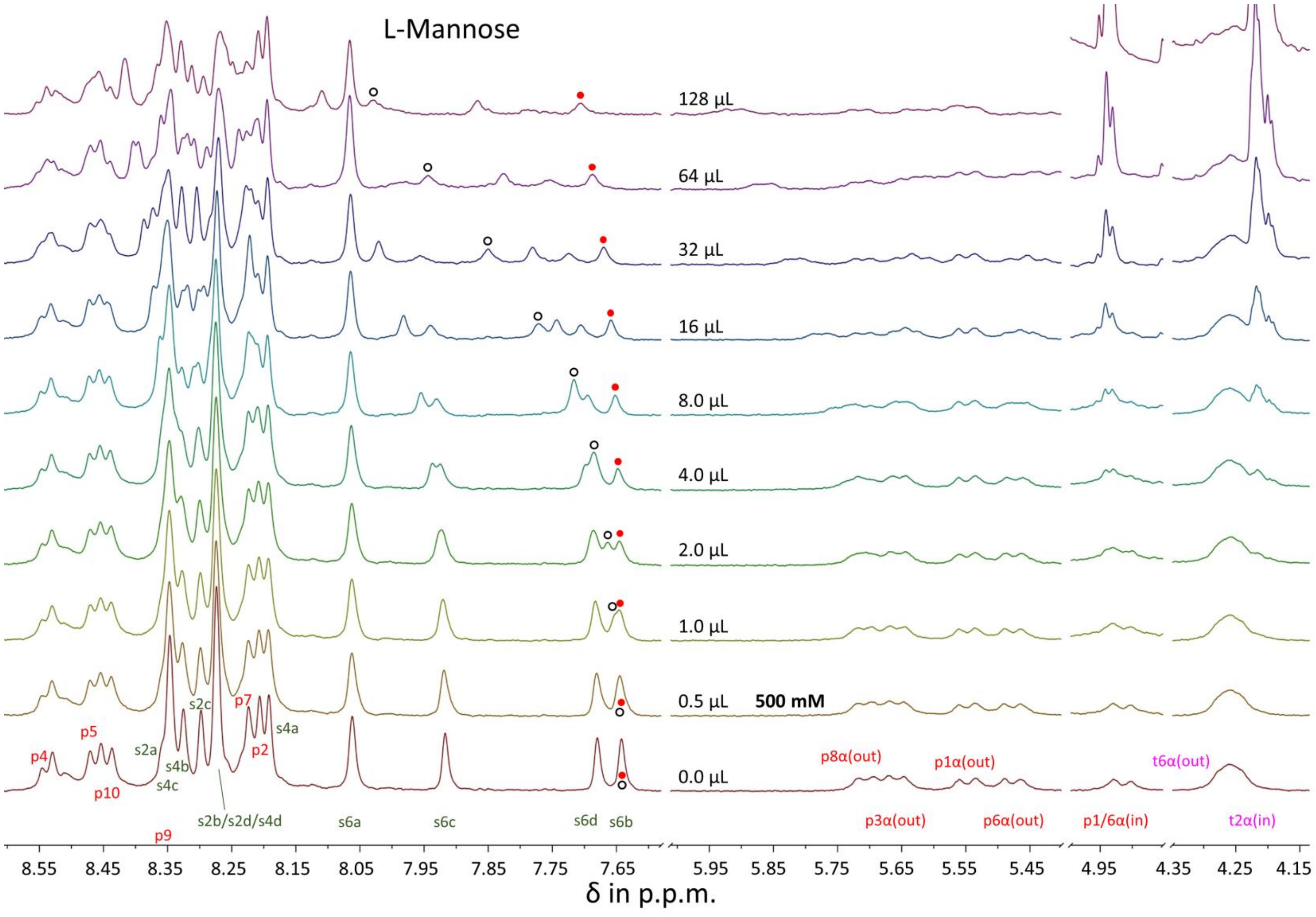

Figure S31. Partial ¹H NMR spectra from the titration of receptors (±)-**2** (0.15 mM each, 400 μL) with L-mannose **12** (500 mM). Peaks marked with empty and red circles were analysed to give binding constants for the diastereomeric complexes (see Figure S32).

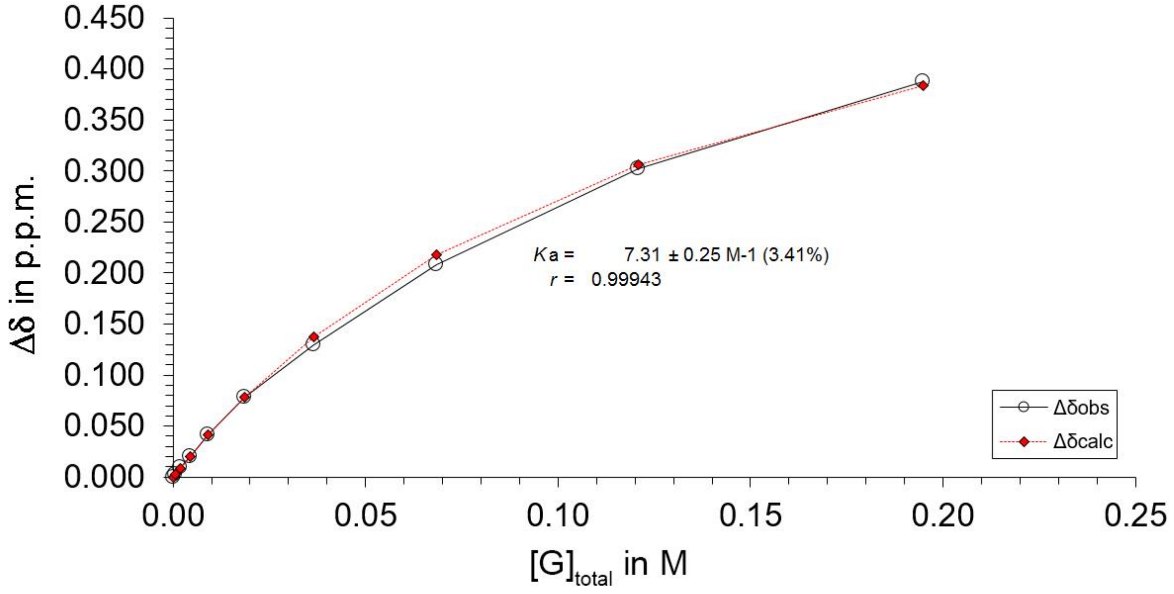

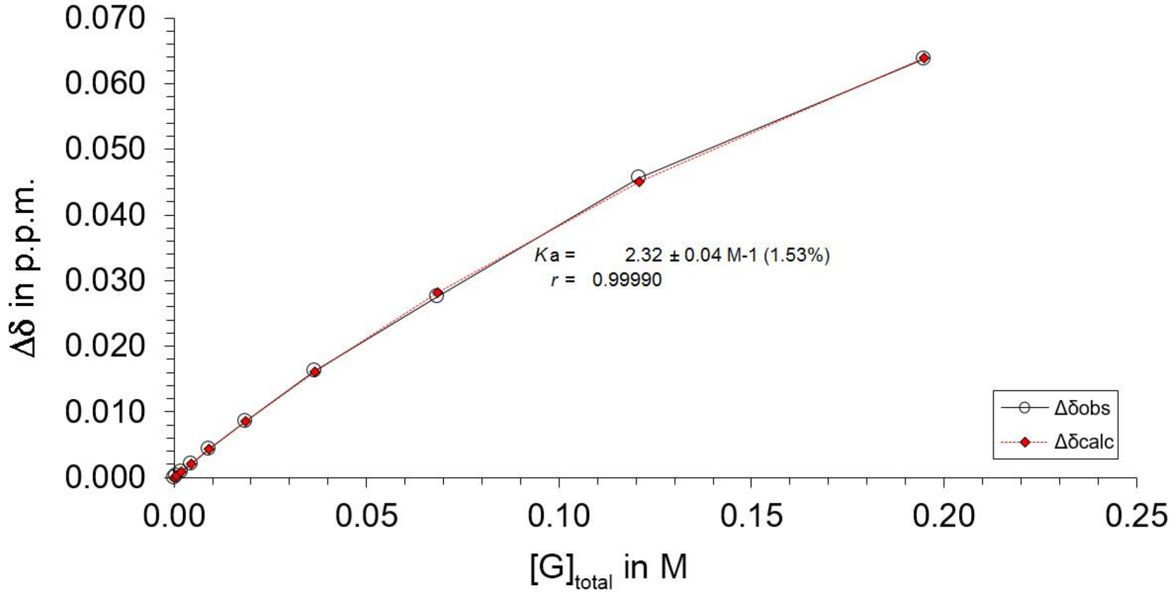

Figure S32. Data analysis for ^{1}H NMR titration of receptors (±)-**2** with L-mannose **12** (see Figure S31). Top: Peak marked with empty circles in Figure S31, K_a = 7.31 ± 0.25 M^{-1} (3.41 %); r = 0.99943; limiting Δδ = 0.653 p.p.m.. Bottom: Peak marked with red circles in Figure S31, K_a = 2.32 ± 0.04 M^{-1} (1.53 %); r = 0.99990; limiting Δδ = 0.205 p.p.m..

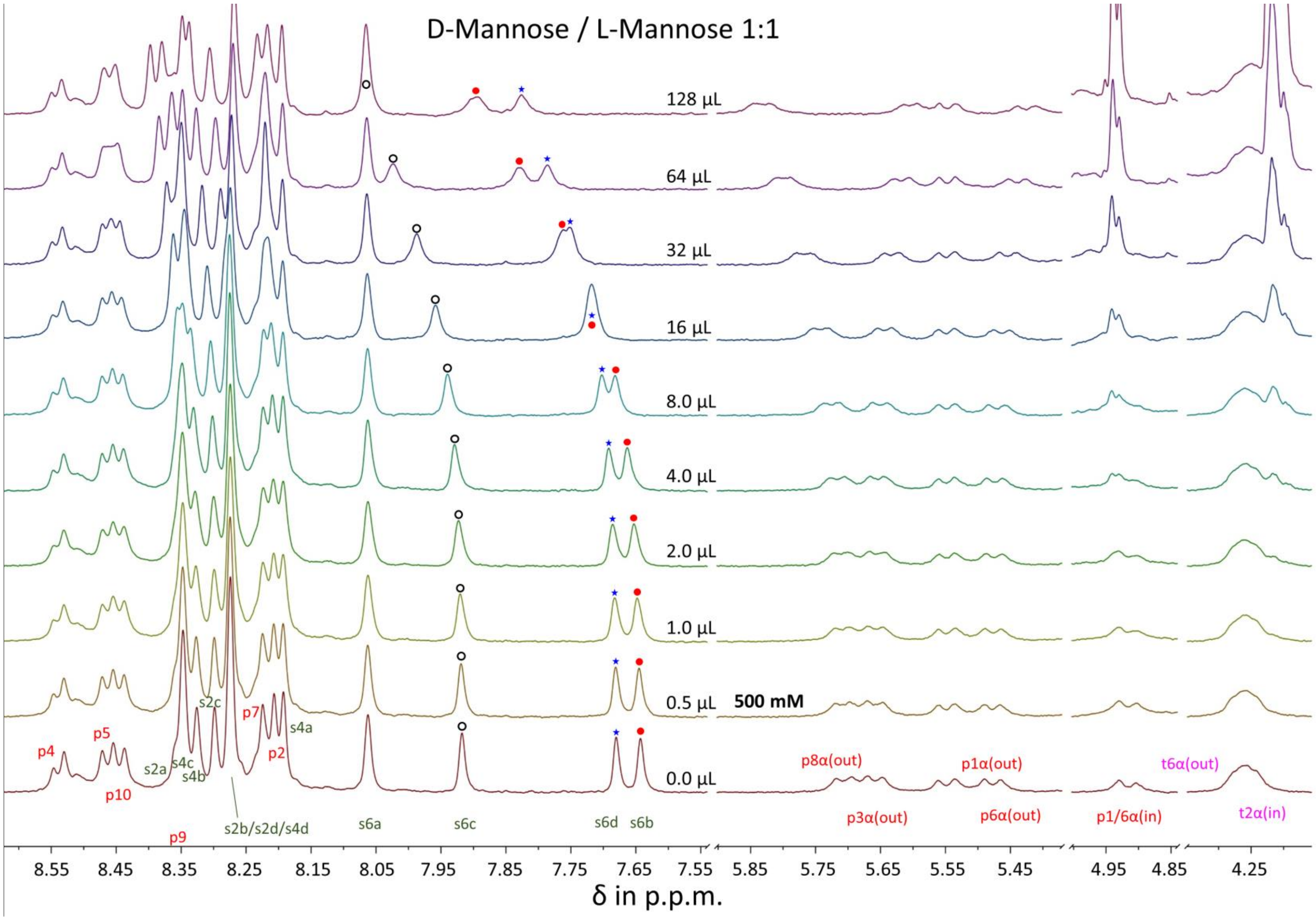

Figure S33. Partial ^{1}H NMR spectra from the titration of receptors (±)-**2** (0.15 mM each, 400 μL) with a 1:1 mixture of D-mannose **11** (250 mM) and L-mannose **12** (250 mM).

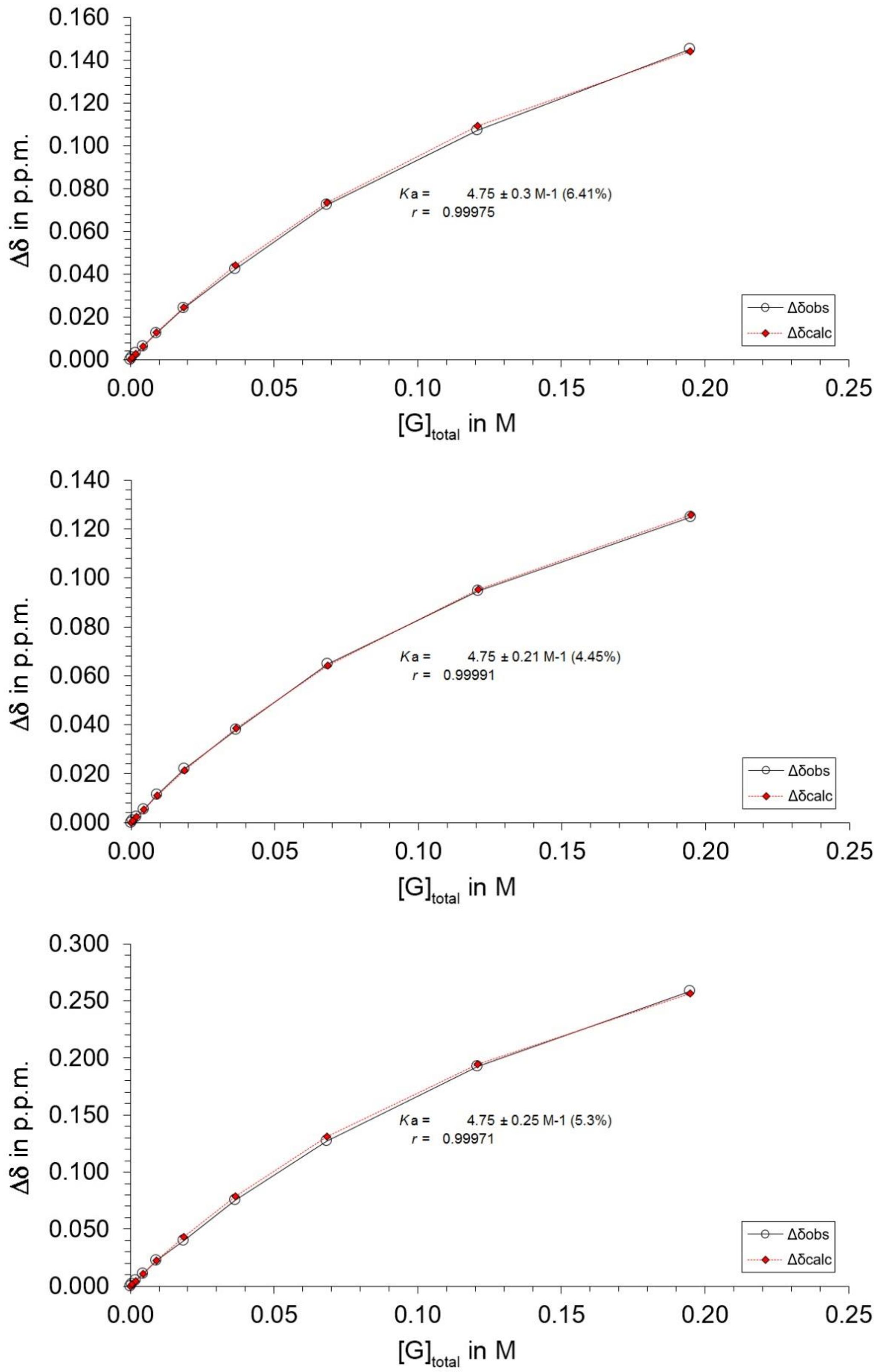

Figure S34. Data analysis for ^{1}H NMR titration of receptors (±)-**2** with a 1:1 mixture of D-mannose **11** and L-mannose **12** (see Figure S33), assuming a 500 mM 'mannose' concentration. Top: Peak marked with empty circles in Figure S33, K_a = 4.75 ± 0.30 M^{-1} (6.41 %); r = 0.99975 ; limiting Δδ = 0.300 p.p.m.. Middle: Peak marked with blue asterisk in Figure S33, K_a = 4.75 ± 0.21 M^{-1} (4.45 %); r = 0.99991 ; limiting Δδ = 0.262 p.p.m.. Bottom: Peak marked with red circle in Figure S33, K_a = 4.75 ± 0.25 M^{-1} (5.30 %); r = 0.99971 ; limiting Δδ = 0.533 p.p.m.. Fit for all three combined: K_a = 4.75 ± 0.25 M^{-1} (5.23 %); r = 0.99975.

3. Identifying the stronger-bound enantiomer from the (±)2 + 8 titration

Summary

To assist discussion, it is useful first to label the enantiomers of **2** according to standard practice. Following Eliel et al.,[6] the bicyclic structure of **2** is considered planar chiral, in which chiral plane is that containing the pyrene unit and pendant spacer. To assign a descriptor one first locates the "pilot atom". This is the out-of-plane atom which is closest to the plane and, compared to others at the same distance, is closest to the atom which ranks highest according to CIP rules. In this case the pilot atom is the N attached to p3α, which earns highest priority through its proximity to the CO_2 group on the pendant spacer (Figure S35). The sequence of in-plane atoms attached to the pilot atom is labelled a,b,c, choosing atoms of highest CIP precedence where necessary. In this case c is carbon p3a, rather than p2. When viewed from the pilot atom, the order of a,b,c (clockwise or anticlockwise) determines whether the descriptor should be p*R* or p*S* (the prefix p denoting planar chirality). The enantiomer shown in Figure S35 is therefore p*S*.

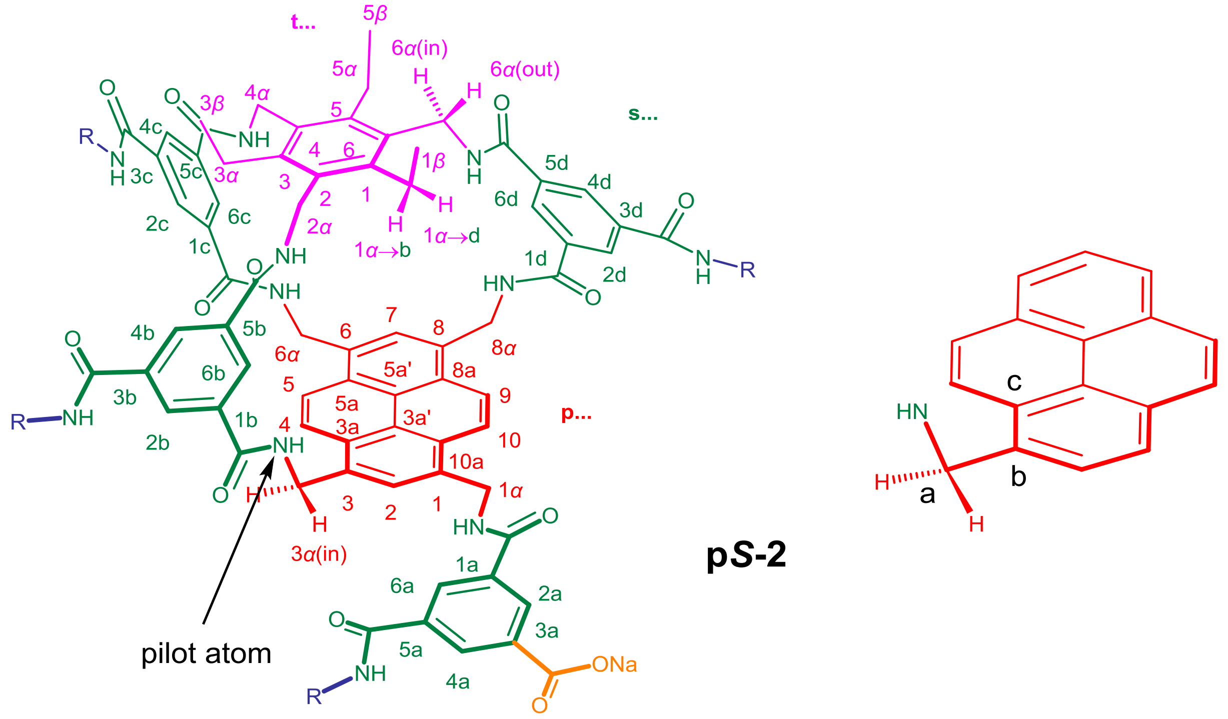

p*S*-2

Figure S35. Configurational assignment of planar chiral receptor **2**, illustrated for the p*S* enantiomer.

[6] E. L. Eliel, S. H. Wilen and L. N. Mander, *Stereochemistry of Organic Compounds*, Wiley, New York, 1994, pp 1121-1122.

As we could not find an unambiguous NMR assignment of *N*-acetyl-D-glucosamine **8** in the literature, it was decided to perform this characterisation as part of the present work. Spectra of **8** in D_2O at 25 °C, and a full assignment of both anomeric forms are given in Figure S39 to
Figure **S44**.

To determine which enantiomer bound more strongly to *N*-acetyl-D-glucosamine **8** (see Figure S25 for the titration experiment) we analysed a mixture of (±)-**2** + **8** by means of {^1H-^1H}-NOESY and -TOCSY NMR spectroscopy at 600 MHz. The concentrations were selected to maximise the number of resonances that could be resolved (particularly the inwards facing s6's), while at the same time having a reasonable concentration of the complex with the stronger binding receptor. We used a 0.42 mM total receptor concentration (so 0.21 mM of each enantiomer) and an *N*-acetyl-D-glucosamine **8** concentration of 10.6 mM. This was chosen by gradually increasing the concentration of **8**; see stacked spectra in Figure S36. Under these conditions the receptor with binding constant K_a = 1280 M^{-1} will be approximately 93% bound, while the weaker binder (K_a = 80 M^{-1}) will be about 46% bound to **8**. With the stronger bound complex about twice as populous as the weaker bound complex, it is to be expected that the stronger bound complex could be (partially) assigned with NMR spectroscopy and attributed to receptor p*R*-**2** or p*S*-**2**. The resulting spectra are shown in
Figure **S45** to
Figure **S52** (NOESY, τ = 500 ms) and
Figure **S53** to
Figure **S55** (TOCSY), including a nearly full assignment of the stronger bound enantiomer (and several assignments of the weaker bound enantiomer). These spectra are also labelled with two capital letters which represent the spectral region on the vertical and horizontal axes respectively: **A** (the aromatic region of 7.65 – 8.70 p.p.m.), **B** (the methylene region of 4.30 – 6.20 p.p.m.), **C** (the carbohydrate region of 3.20 – 3.90 p.p.m.) and **D** (the aliphatic region of 0.50 – 2.70 p.p.m.).[7]

[7] NB: after fully processing the 2D spectra it appeared that the calibration was slightly in error. The chemical shift values in Figures S45-S55 are thus offset by about 0.1 p. p. m.

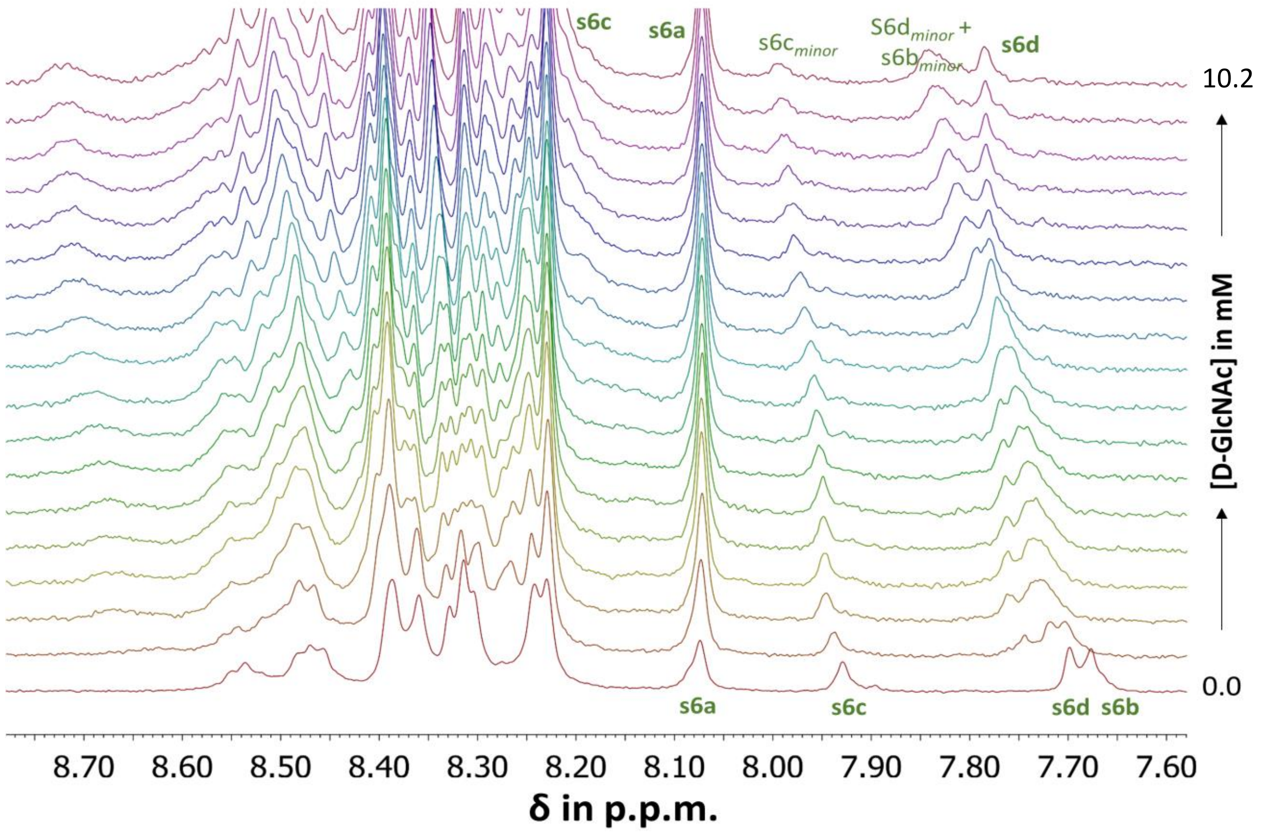

Figure S36. Partial ^{1}H NMR spectra of the titration in D_2O employed to generate the solution of (±)-**2** + **8** for structural studies. The final total concentration of *N*-acetyl-D-glucosamine **8** is 10.6 mM. The concentration of receptors is 0.42 mM in total (0.21 mM of each).

The entry point into the spin system of the complexes are the **s6c** protons (7.93 ppm). It is clear that during the titration this resonance is shifted downfield for both enantiomers, but that the limiting chemical shift for one is much larger than for the other; already early on in the 'titration' the visible **s6c** integrates as one proton, meaning that the other **s6c** has shifted (to 8.19 ppm, as appeared during the assignment). This is also the case for protons **s6b**, although the resonance of the major species could not be identified. s6b$_{minor}$ is overlapped with s6d$_{minor}$ around 7.84 ppm. **s6d** (major) can clearly identified at 7.76 ppm and **s6a** is essentially unperturbed and appears as a single peak at 8.07 ppm.

It was possible to almost completely assign the resonances for the enantiomer representing the major species present. There were sufficient resolved nuclear Overhauser effect cross peaks (NOEs) between this enantiomer and the carbohydrate. The resonances of the minor species were obviously less abundant and were broadened (e.g. s6b$_{minor}$, s6c$_{minor}$ and S6d$_{minor}$ resonances) yielding

only very weak NOEs. Only clearly distinguished NOEs of the major species were used in the analysis to safeguard against possible confounding correlations arising from the minor species, however weak these might be. A full labelling scheme for both enantiomers of **2**, as well as the numbering for D-GlcNAc (**8**), are shown in

Figure S37.

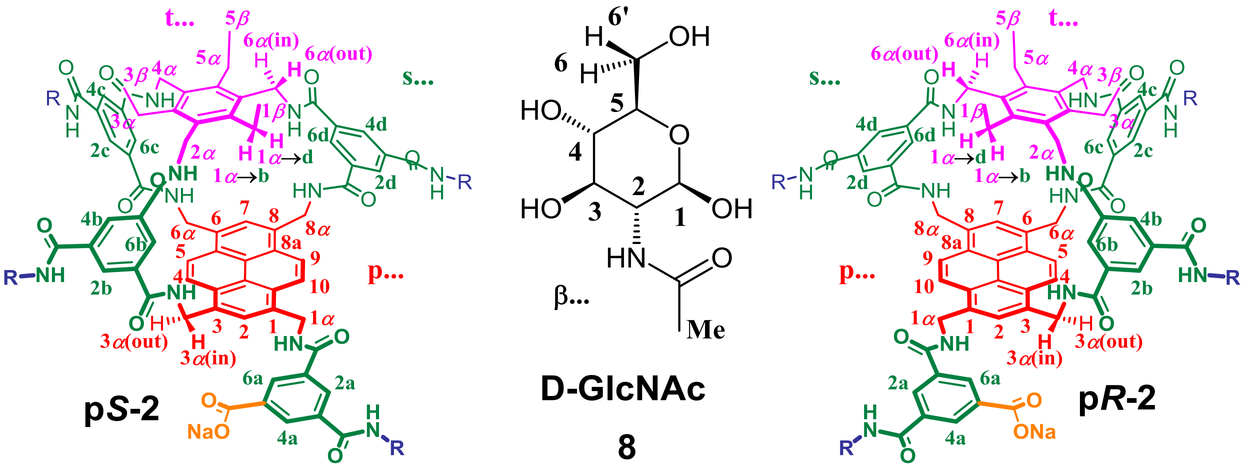

Figure S37. Labelling system used for the enantiomers of **2** and for D-GlcNAc **8**.

From the carbohydrate region (3.2 – 3.9 ppm, **C**), it is evident that the **α** and the **β** isomers were present, but that the resonances of the **β** were broadened. These resonances were sharp in a spectrum of pure D-GlcNAc (see Figure S39). None of the **α** protons display an NOE with any of the receptor resonances whereas there are very clear NOEs with the other hydrogen atoms of D-GlcNAc; **β-2**, **β-5**, **β-6**, **β-6'**, and **β-Me**. Potential NOEs with the anomeric proton **β-1** (should come around 4.62 ppm) were obscured by the overlapping water resonance and indeed no such NOEs could be observed. Protons **β-3** (~3.48 ppm) and **β-4** (~3.38 ppm) were be identified (although overlapping with **α-4**). There were no NOEs discernible with **β-3** and only two very weak NOEs with **β-4** (*NB: one of these is crucial to differentiate between p*R-***2*** *and p*S-***2***). This means they must be pointing towards the aromatics, furthest away from other H-atoms.

The carbohydrate can be approximately positioned inside the cage by considering the NOEs with the acetyl methyl resonance **β-Me** (1.976 ppm) and the carbohydrate's methylene resonances **β-6** (3.833 ppm) and **β-6'** (3.679 ppm). NOEs to the **β-Me** indicate that this unit is emerging from the largest portal, between **t1α/β** and **p2**, and in close proximity to **s6d**, **p1α** and **p10**. The

carbohydrate's CH_2OH is positioned in the smallest portal, between **t5α/β** and **p7**, and is also close to **s6c** (with **β-6** pointing towards **s6c** and **β-6'**pointing towards **t5α/β**).

That the methylene (particularly **β-6'**) is placed close to **t5α/β** while **β-Me** is positioned close to **p2** provides information on the orientation of D-GlcNAc relative to the triethylbenzene and pyrenyl aromatics; **β-1/3/5** point 'downwards' towards pyrene while **β-2/4** point 'upwards' towards triethylbenzene. Indeed, **β-5** is close to **p7** and **p5**, while **β-2** is close to **t1α/β**.

The imaginary line **β-Me→β-2→β-5→β-6/6'** through D-GlcNAc is straight and can thus fit equally well in both p*R*-**2** and p*S*-**2** with **β-Me** emerging from the largest (below **t1α/β**) and **β-6/6'** from the smallest (below **t5α/β**) portal (NB: the C5-C6 bond can rotate so that **β-6/6'** can always point to **s6c**). There are no NOEs observed with **β-1** and **β-3**. This makes the NOE observed between **β-4** and **t1β** crucial (Figure S51); this NOE can only exist in [p*R*-**2** · D-GlcNAc]. Indeed, in [p*S*-**2** · D-GlcNAc] **t1β** is located opposite **β-4**. This NOE was also observed in a NOESY experiment at 35 °C.

It is therefore concluded that D-GlcNAc **8** in bound as the β-anomer preferentially by the p*R* enantiomer of **2**, in the orientation shown in Figure S38a. A 3D model consistent with the NOE data is shown in Figure S38b.

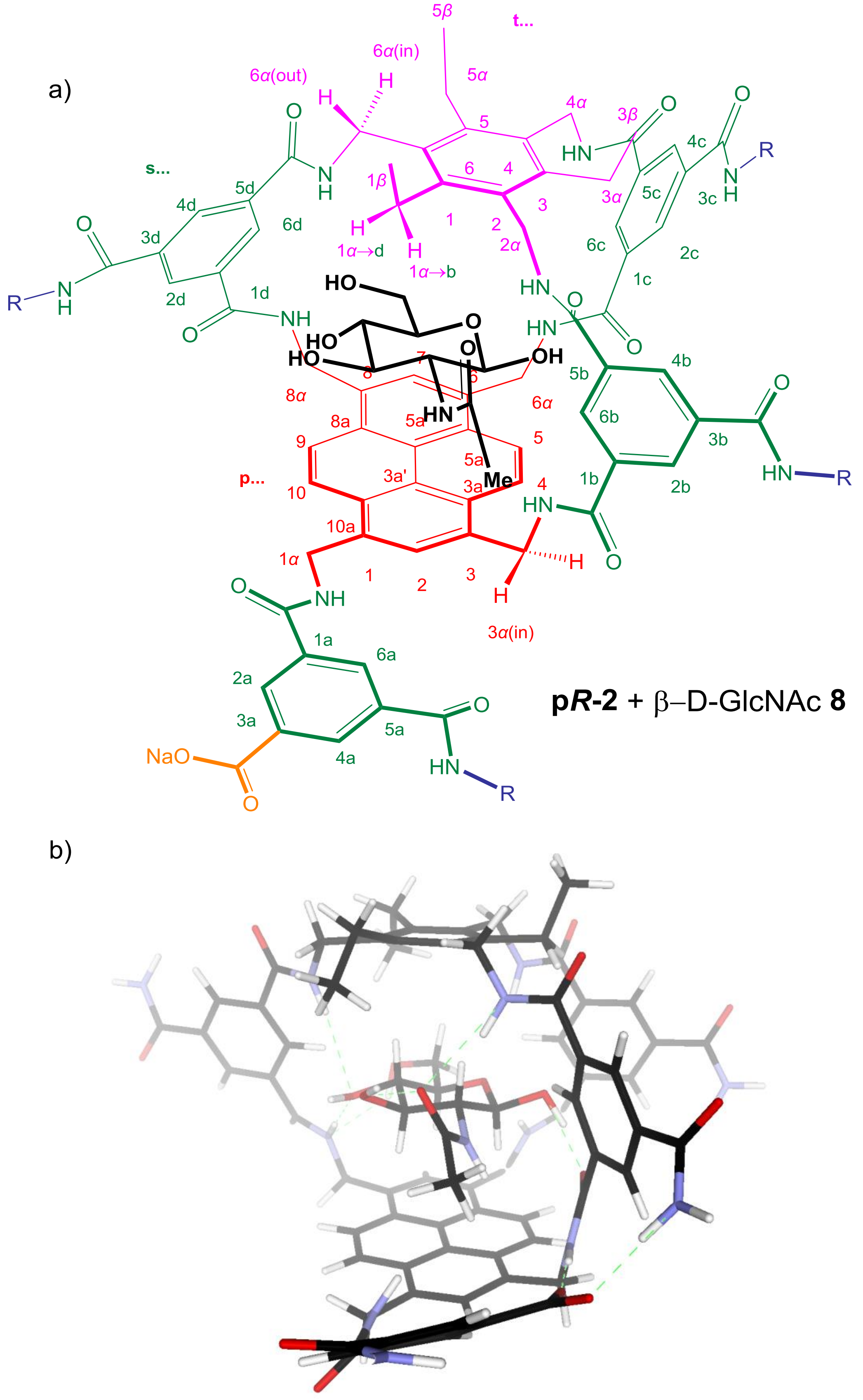

Figure S38. a) The orientation of β-D-GlcNAc **8** in the stronger-binding p*R* enantiomer of **2**, as deduced by ^{1}H NMR NOE spectroscopy. b) 3D model of the complex after minimisation with Spartan molecular modelling software (Spartan ’16 v 2.0.3, molecular mechanics minimisation using the MMFF force field with explicit aqueous solvation)**.**

NMR spectra and assignments of *N*-acetyl-D-glucosamine **8**

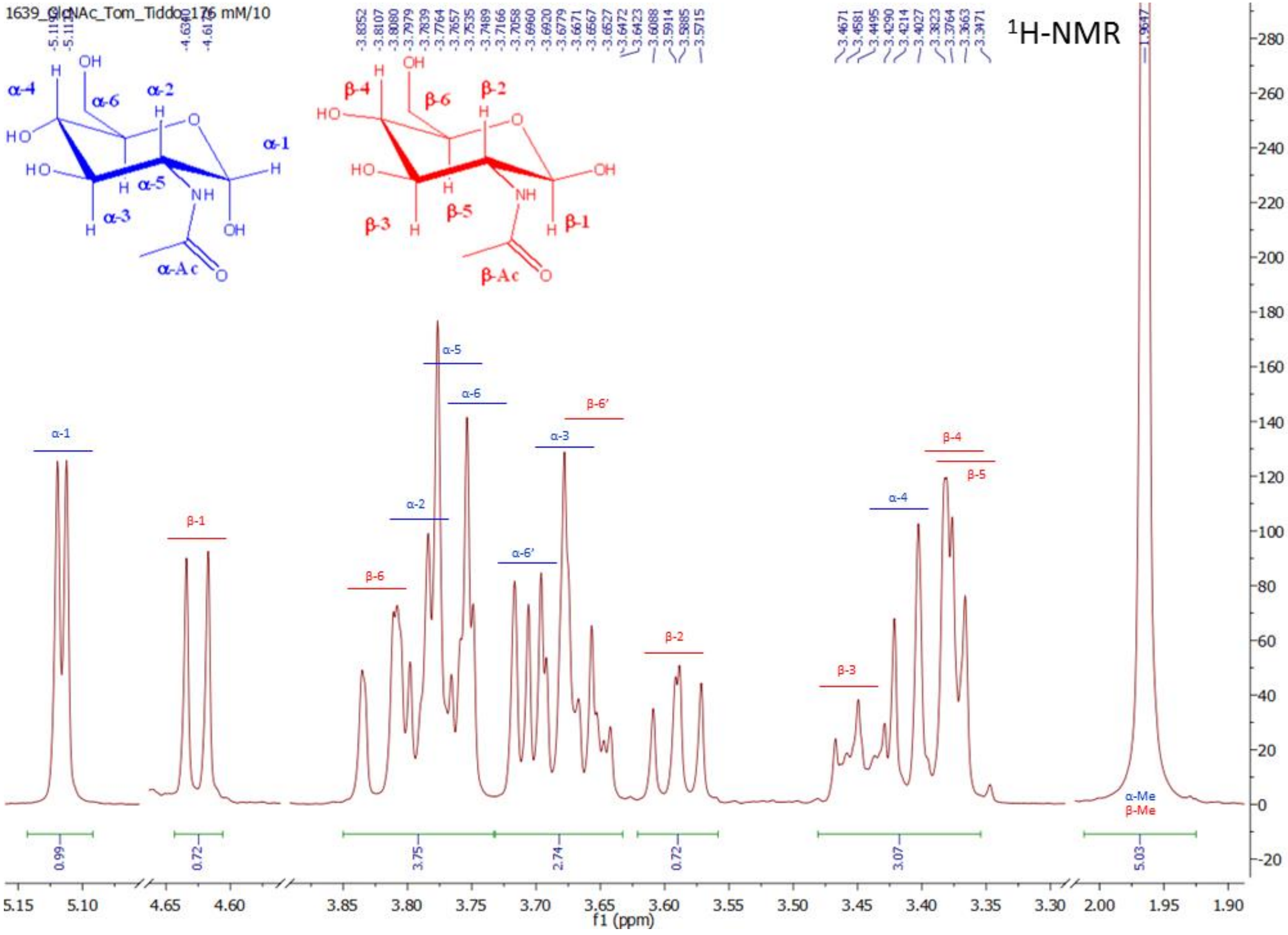

Figure S39. ^{1}H NMR spectrum of 176 mM *N*-acetyl-D-glucosamine **8** in D_2O including assignments of the alpha (in blue) and beta (in red) anomers**.**

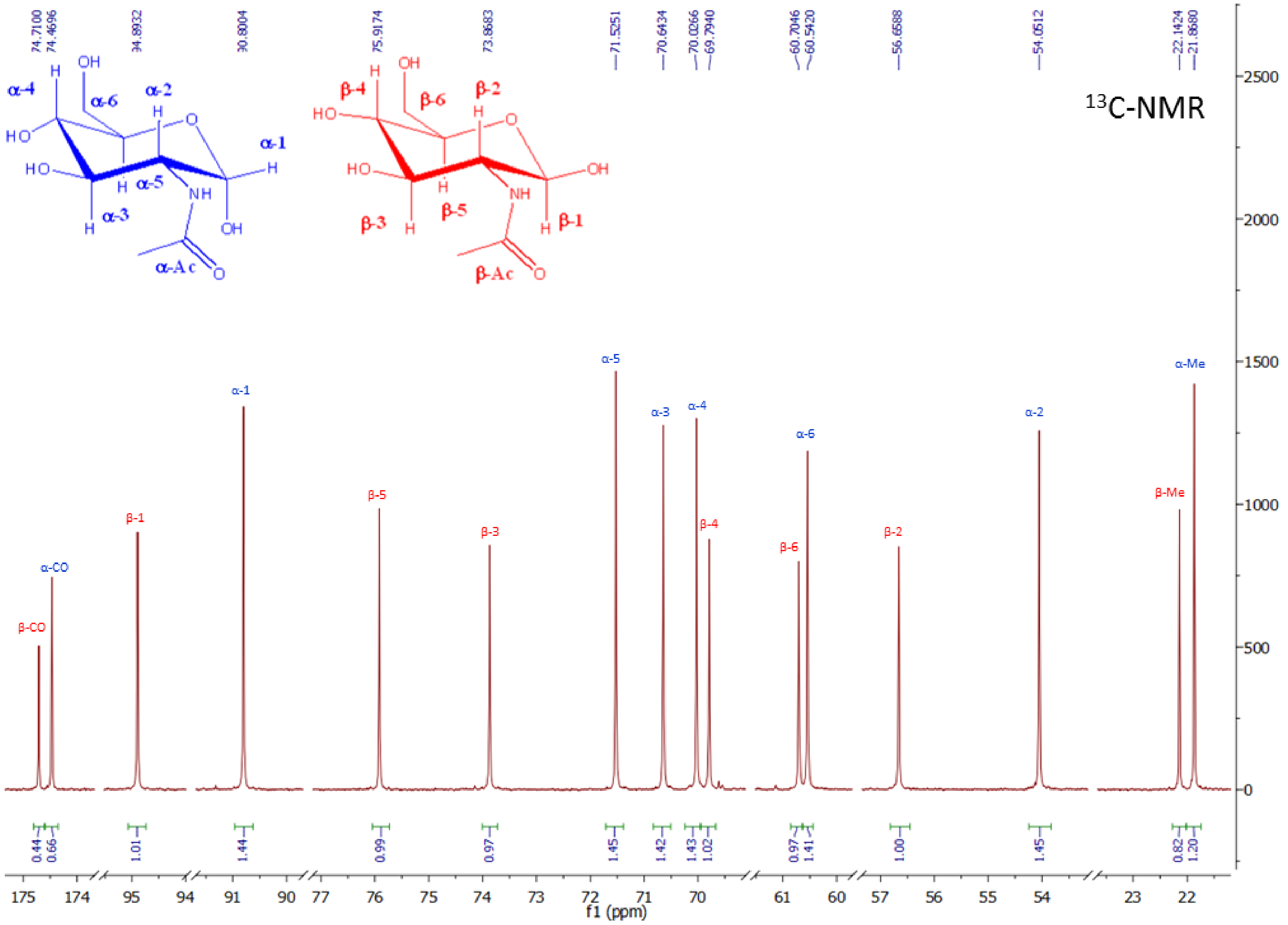

Figure S40. ^{13}C NMR spectrum of 176 mM *N*-acetyl-D-glucosamine **8** in D_2O including assignments of the alpha (in blue) and beta (in red) anomers.

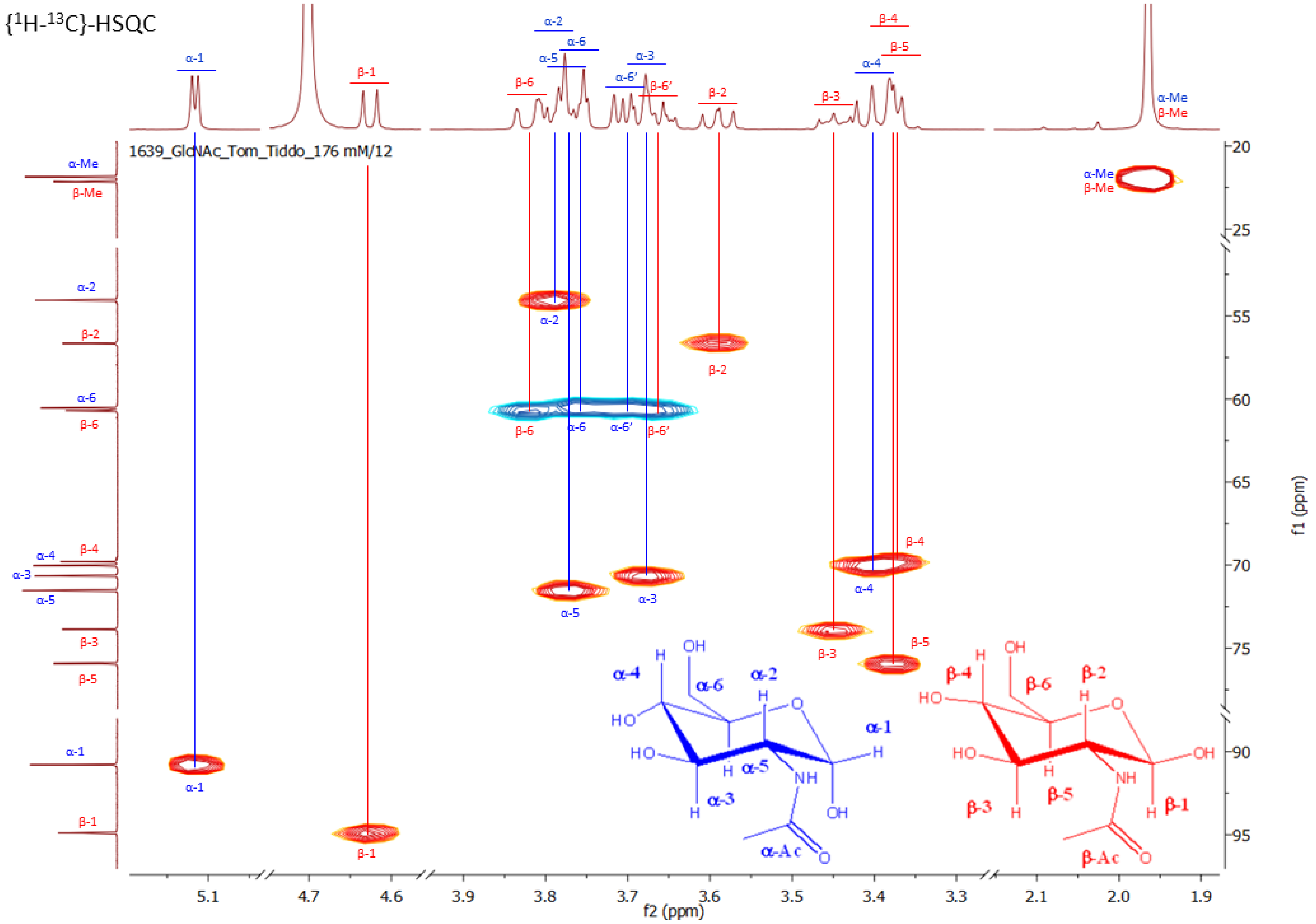

Figure S41. {^{1}H-^{13}C}-HSQC NMR spectrum of 176 mM *N*-acetyl-D-glucosamine **8** in D_2O including assignments of the alpha (in blue) and beta (in red) anomers.

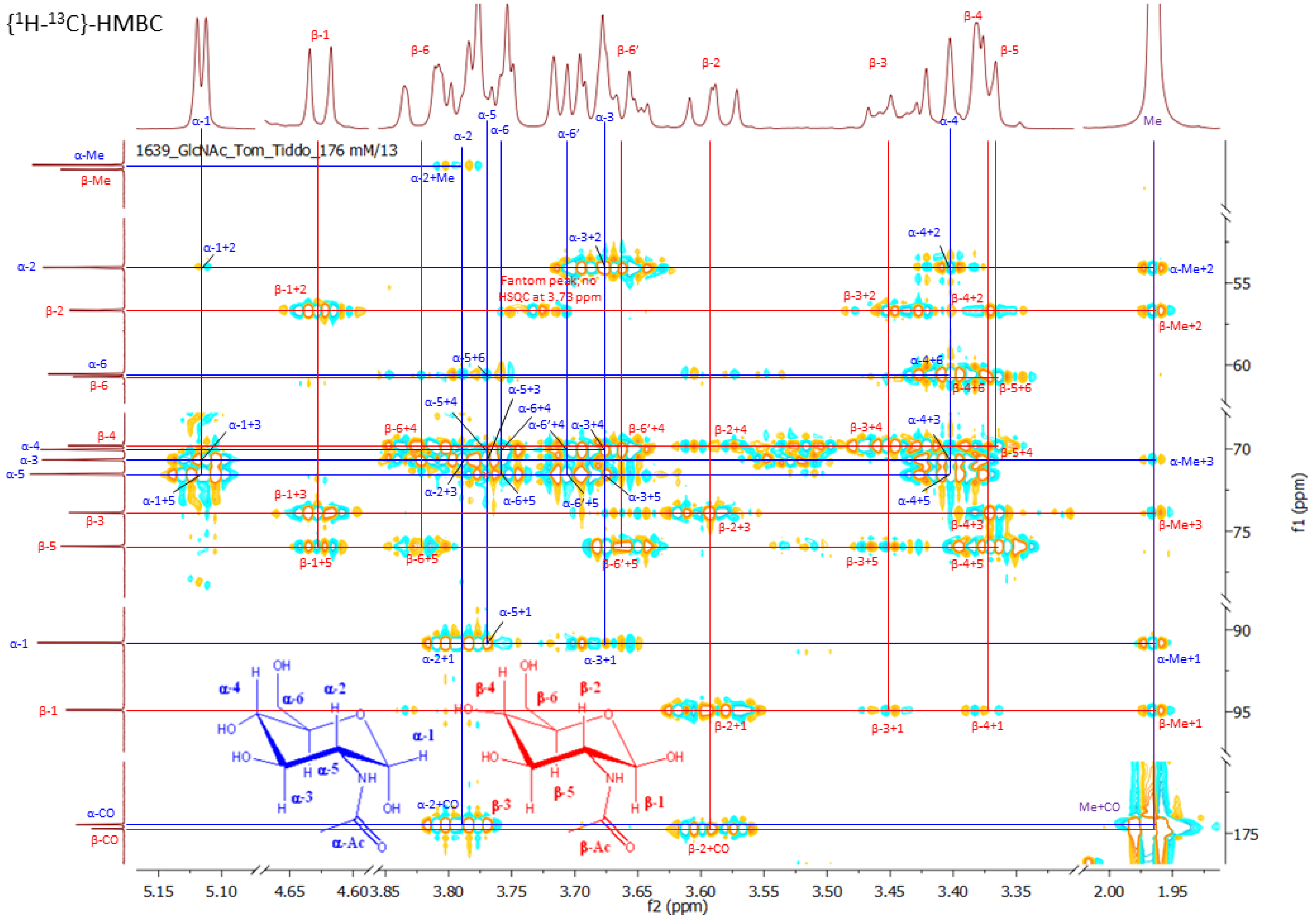

Figure S42. {^{1}H-^{13}C}-HMBC NMR spectrum of 176 mM *N*-acetyl-D-glucosamine **8** in D_2O including assignments of the alpha (in blue) and beta (in red) anomers. See Figure **S43** for a zoom-out.

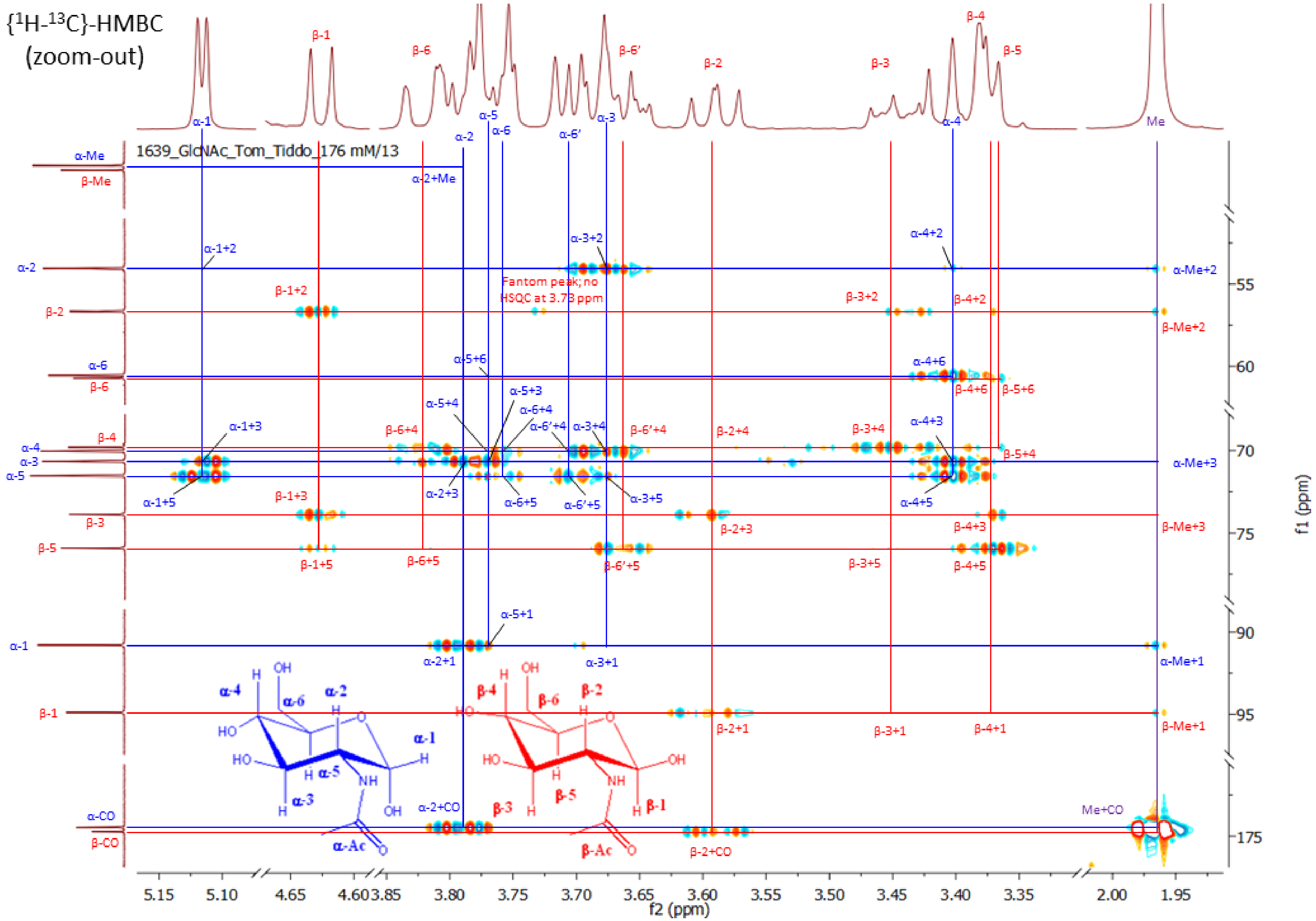

Figure S43. {^{1}H-^{13}C}-HMBC NMR spectrum of 176 mM *N*-acetyl-D-glucosamine **8** in D_2O including assignments of the alpha (in blue) and beta (in red) anomers. See Figure **S42** for a zoom-in.

{^{1}H-^{1}H}-NOESY

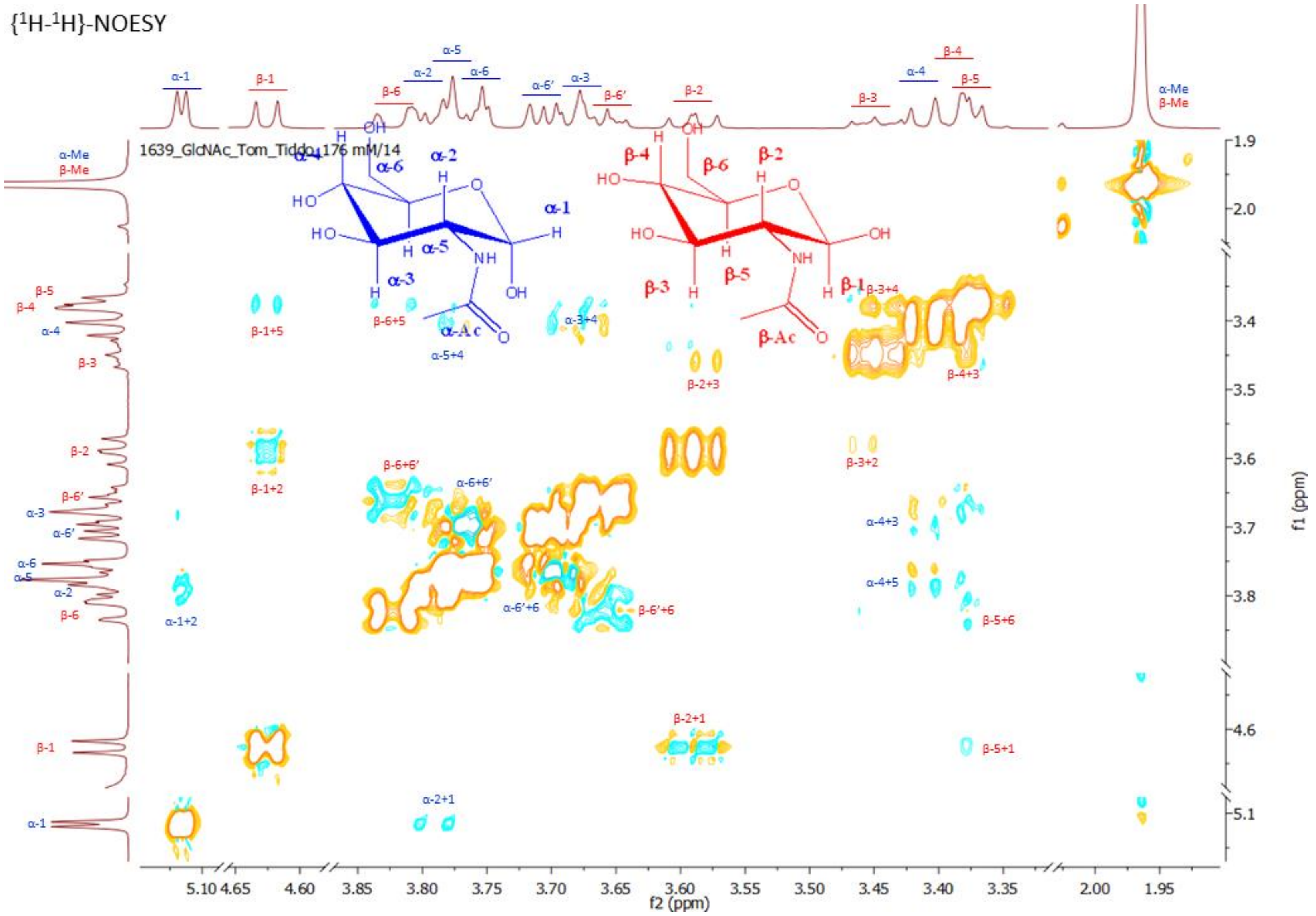

Figure S44. {^{1}H-^{1}H}-NOESY NMR spectrum of 176 mM *N*-acetyl-D-glucosamine **8** in D_2O including assignments of the alpha (in blue) and beta (in red) anomers.

NMR spectra and assignments of of (±)-**2** + **8**

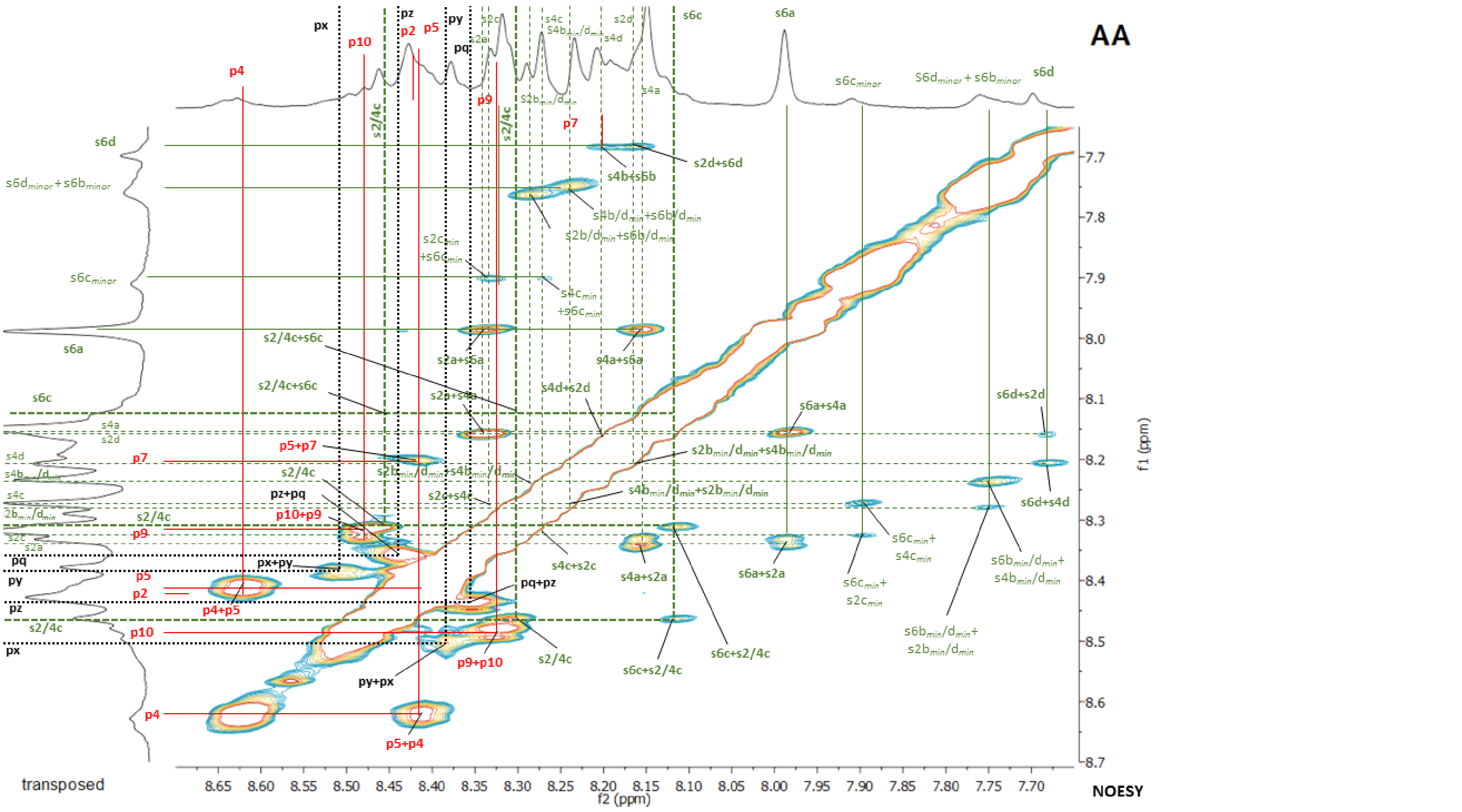

Figure S45. Partial {^{1}H-^{1}H}-NOESY NMR spectrum of receptors (±)-**2** (0.21 mM each) and *N*-acetyl-D-glucosamine **8** (10.6 mM) and in D_2O including assignments. Mixing time = 500 ms; the time domains were transposed to improve visual resolution on the horizontal axis. Note that chemical shifts are offset by about 0.1 p.p.m.[7]

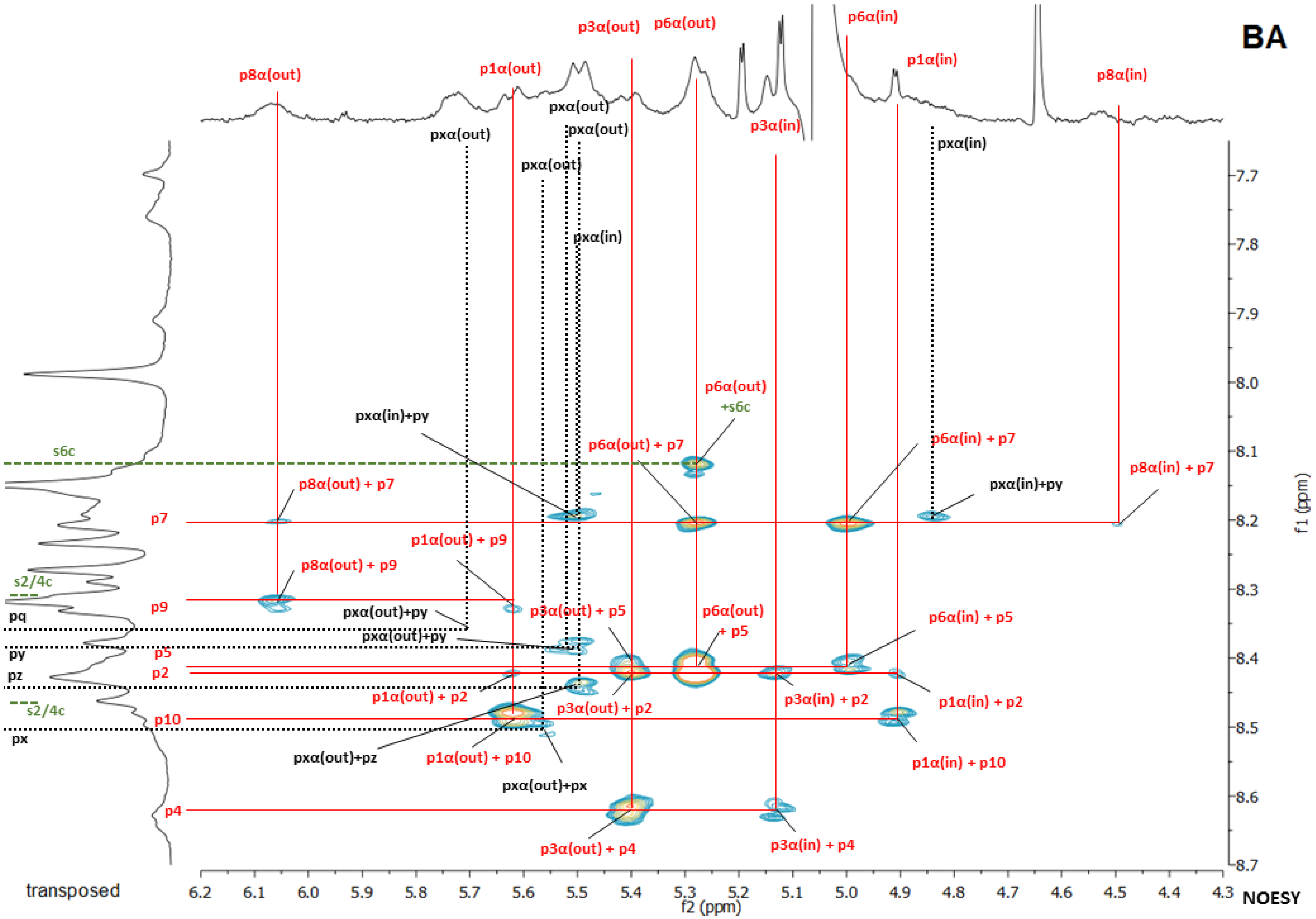

Figure S46. Partial {^{1}H-^{1}H}-NOESY NMR spectrum of receptors (±)-**2** (0.21 mM each) and *N*-acetyl-D-glucosamine **8** (10.6 mM) and in D_2O including assignments. Mixing time = 500 ms; the time domains were transposed to improve visual resolution on the horizontal axis. Note that chemical shifts are offset by about 0.1 p.p.m.[7]

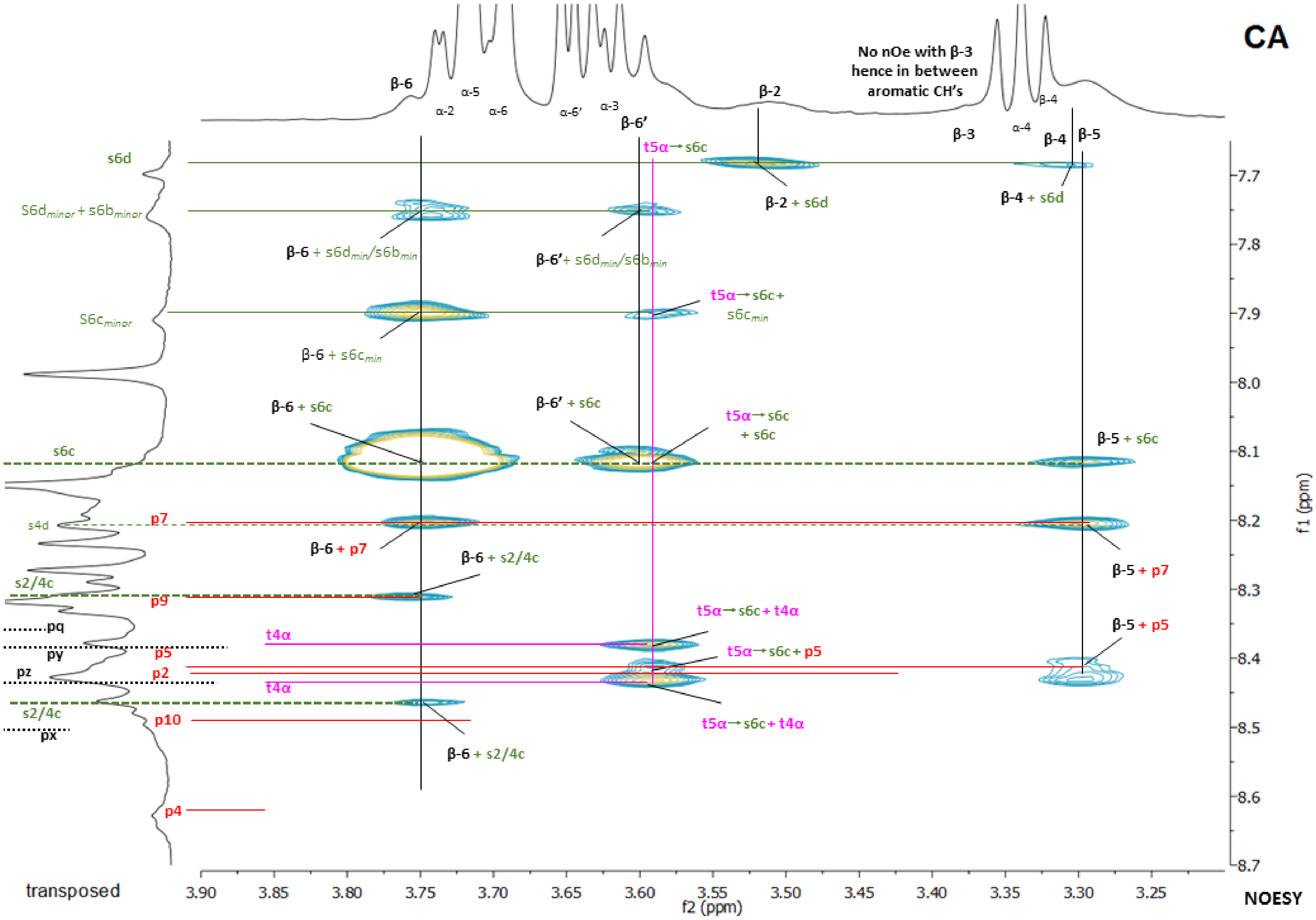

Figure S47. Partial {^{1}H-^{1}H}-NOESY NMR spectrum of receptors (±)-**2** (0.21 mM each) and *N*-acetyl-D-glucosamine **8** (10.6 mM) and in D_2O including assignments. Mixing time = 500 ms; the time domains were transposed to improve visual resolution on the horizontal axis. Note that chemical shifts are offset by about 0.1 p.p.m.[7]

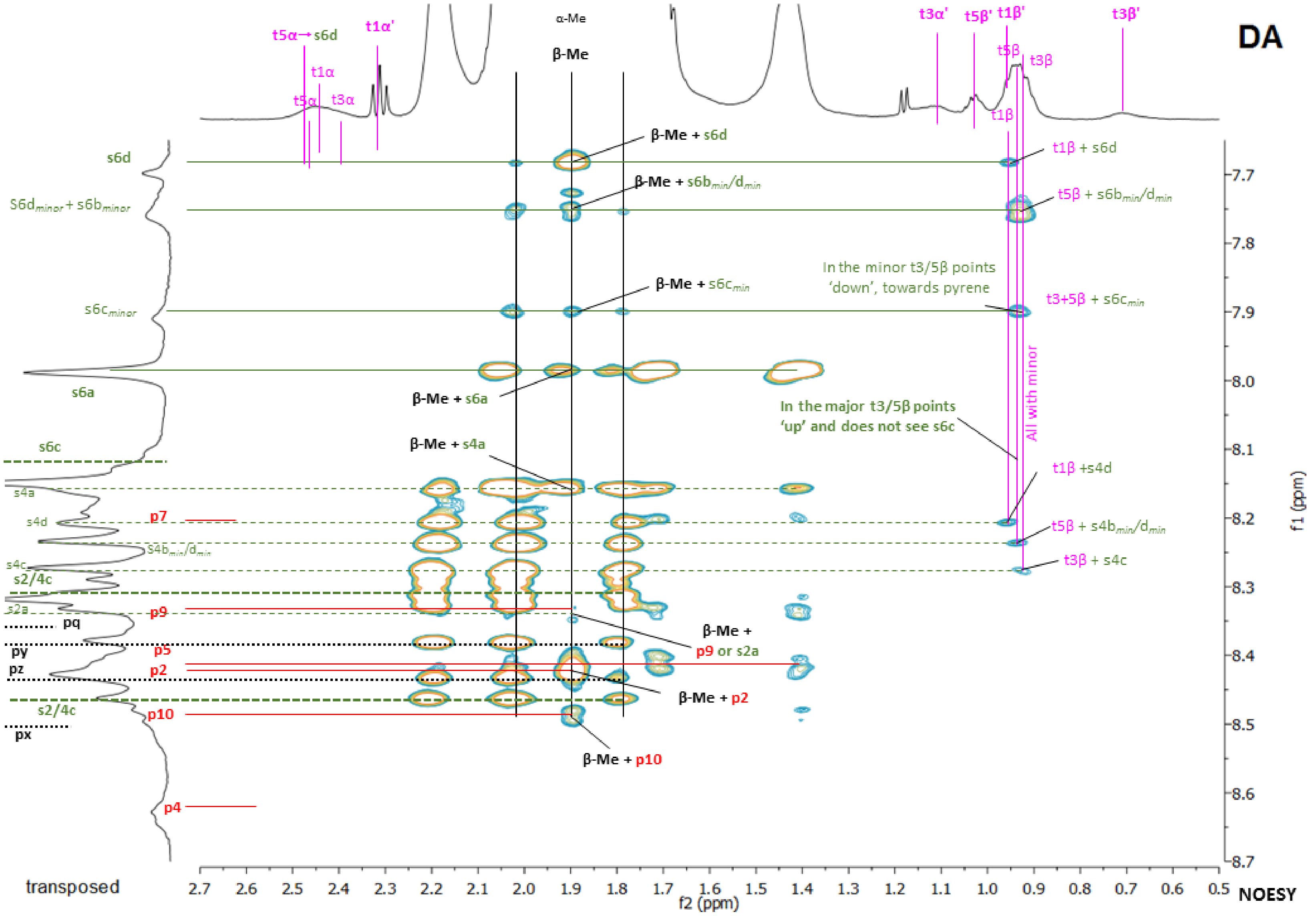

Figure S48. Partial {^{1}H-^{1}H}-NOESY NMR spectrum of receptors (±)-**2** (0.21 mM each) and *N*-acetyl-D-glucosamine **8** (10.6 mM) and in D_2O including assignments. Mixing time = 500 ms; the time domains were transposed to improve visual resolution on the horizontal axis. Note that chemical shifts are offset by about 0.1 p.p.m.[7]

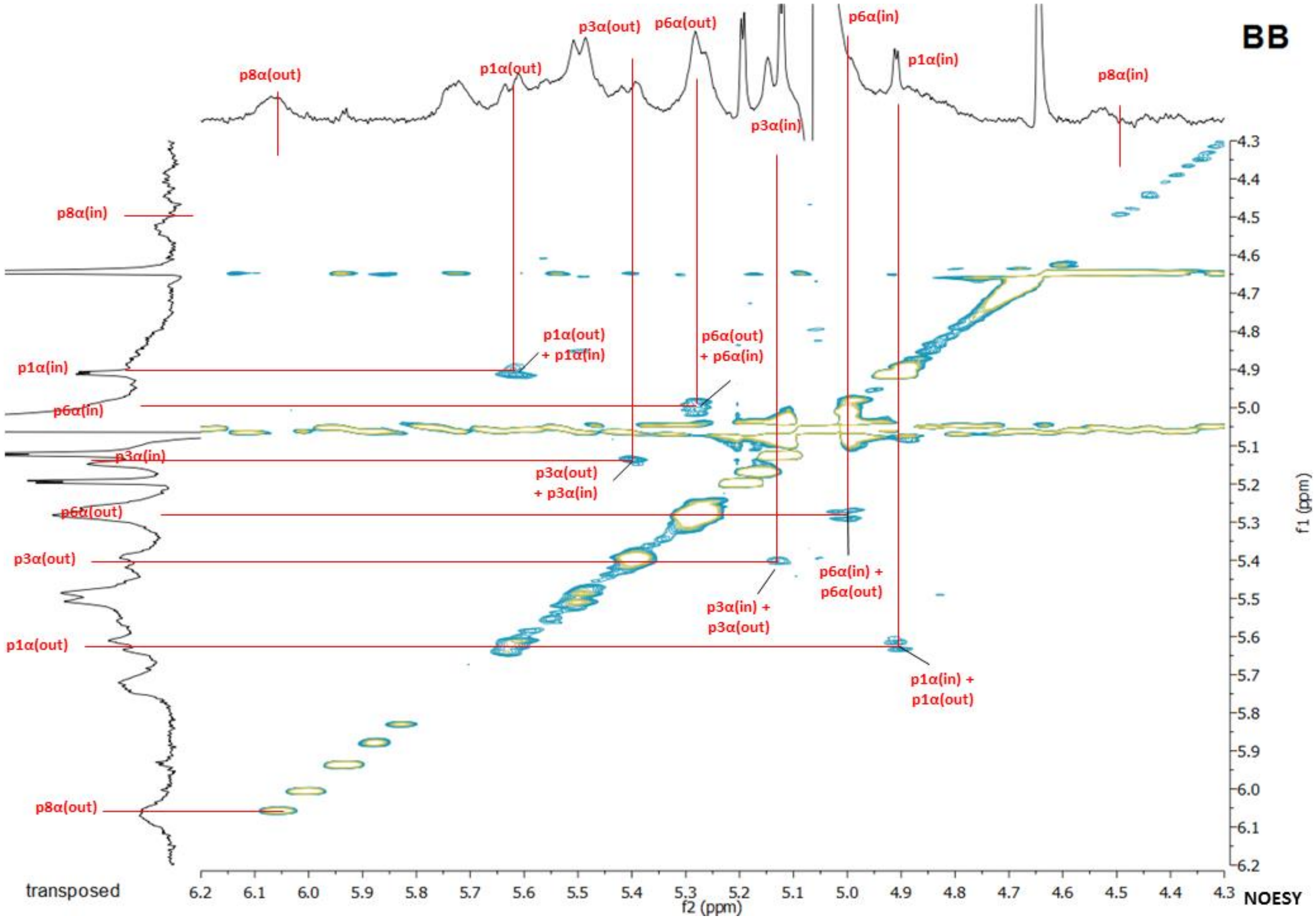

Figure S49. Partial {^{1}H-^{1}H}-NOESY NMR spectrum of receptors (±)-**2** (0.21 mM each) and *N*-acetyl-D-glucosamine **8** (10.6 mM) and in D_2O including assignments. Mixing time = 500 ms; the time domains were transposed to improve visual resolution on the horizontal axis. Note that chemical shifts are offset by about 0.1 p.p.m.[7]

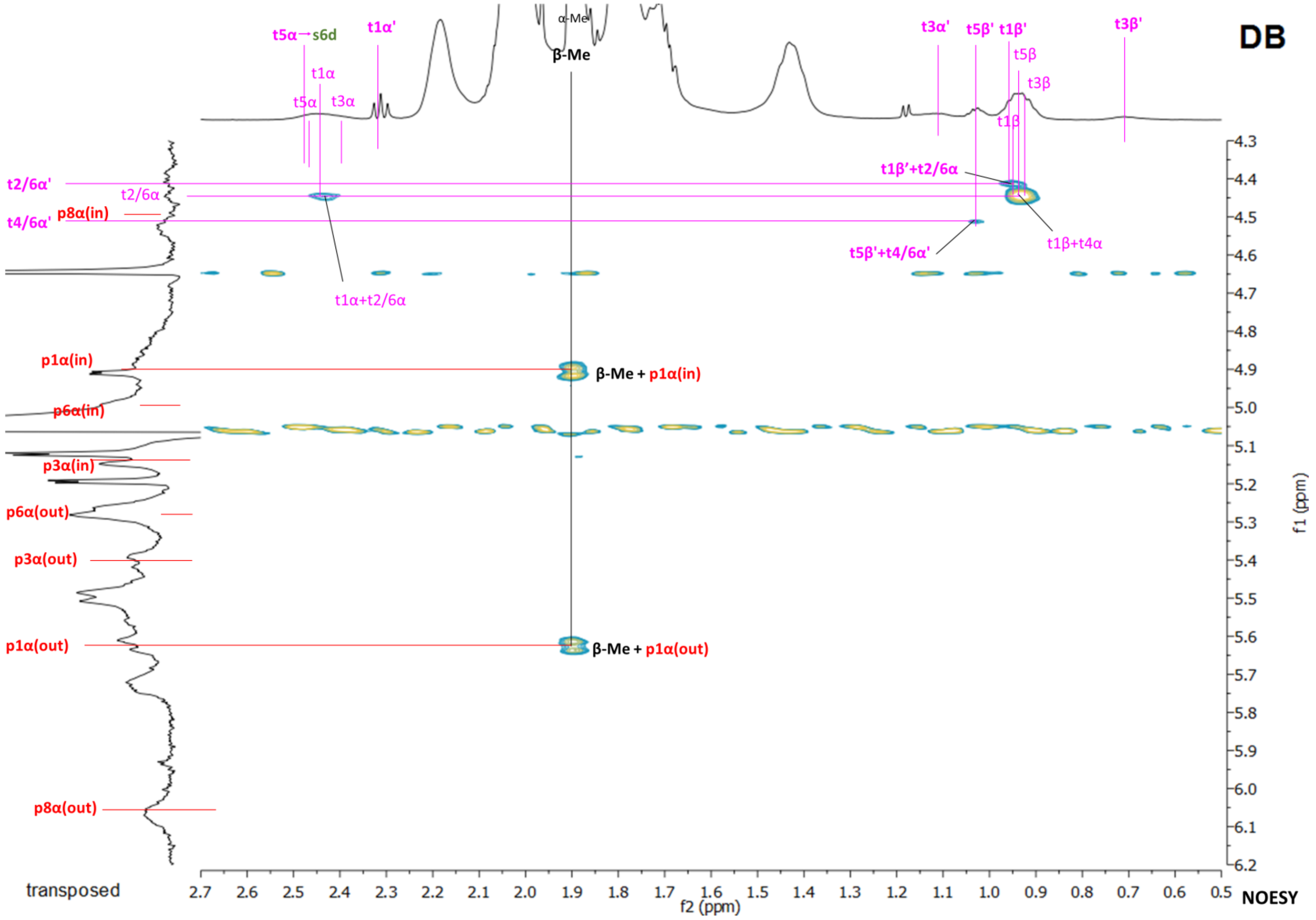

Figure S50. Partial {^{1}H-^{1}H}-NOESY NMR spectrum of receptors (±)-**2** (0.21 mM each) and *N*-acetyl-D-glucosamine **8** (10.6 mM) and in D_2O including assignments. Mixing time = 500 ms; the time domains were transposed to improve visual resolution on the horizontal axis. Note that chemical shifts are offset by about 0.1 p.p.m.[7]

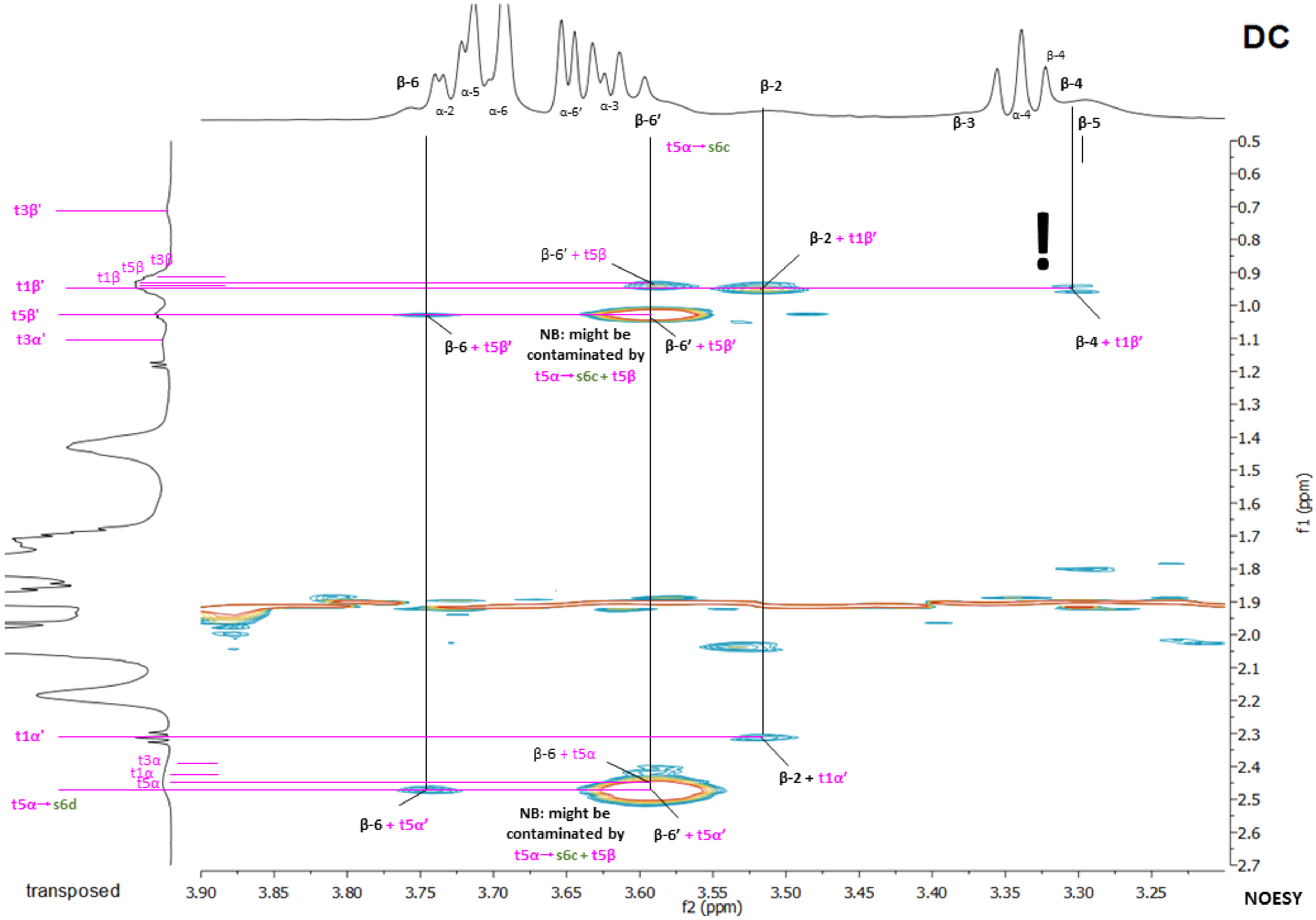

Figure S51. Partial {^{1}H-^{1}H}-NOESY NMR spectrum of receptors (±)-**2** (0.21 mM each) and *N*-acetyl-D-glucosamine **8** (10.6 mM) and in D_2O including assignments. Mixing time = 500 ms; the time domains were transposed to improve visual resolution on the horizontal axis. Note that chemical shifts are offset by about 0.1 p.p.m.[7]

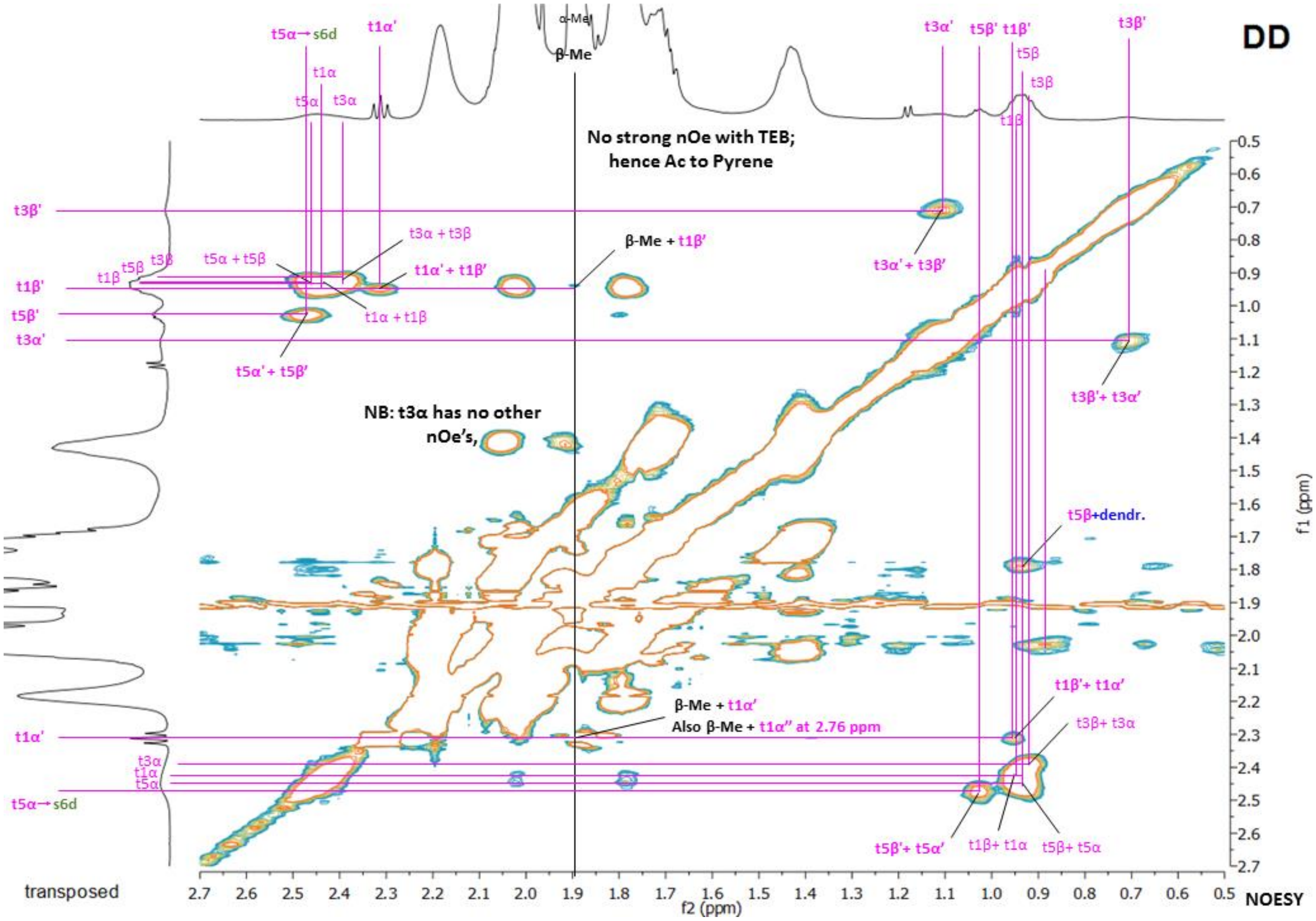

Figure S52. Partial {^{1}H-^{1}H}-NOESY NMR spectrum of receptors (±)-**2** (0.21 mM each) and *N*-acetyl-D-glucosamine **8** (10.6 mM) and in D_2O including assignments. Mixing time = 500 ms; the time domains were transposed to improve visual resolution on the horizontal axis. Note that chemical shifts are offset by about 0.1 p.p.m.[7]

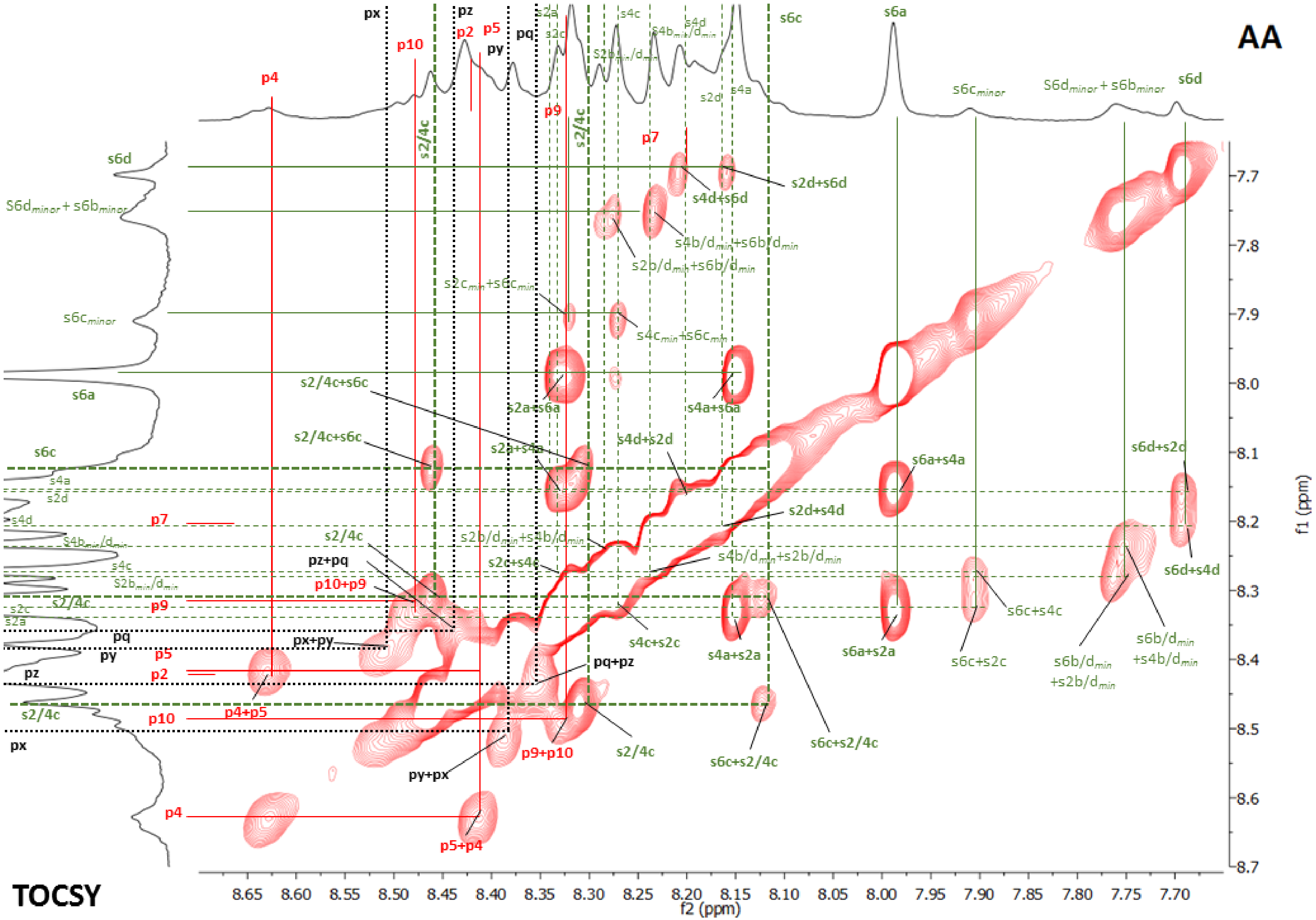

Figure S53. Partial {^{1}H-^{1}H}-TOCSY NMR spectrum of receptors (±)-**2** (0.21 mM each) and *N*-acetyl-D-glucosamine **8** (10.6 mM) and in D_2O including assignments. Note that chemical shifts are offset by about 0.1 p.p.m.[7]

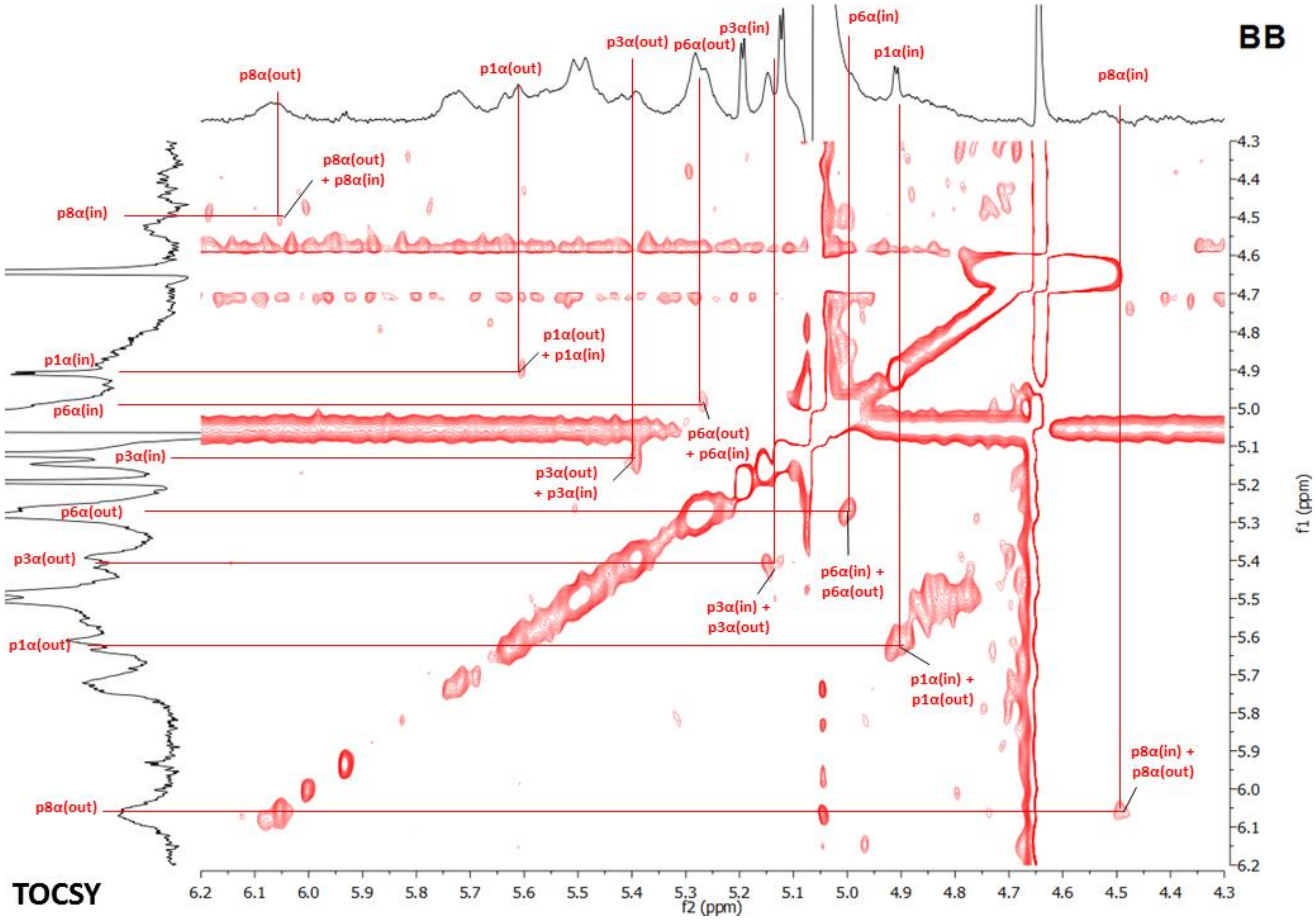

Figure S54. Partial {^{1}H-^{1}H}-TOCSY NMR spectrum of receptors (±)-**2** (0.21 mM each) and *N*-acetyl-D-glucosamine **8** (10.6 mM) and in D_2O including assignments. Note that chemical shifts are offset by about 0.1 p.p.m.[7]

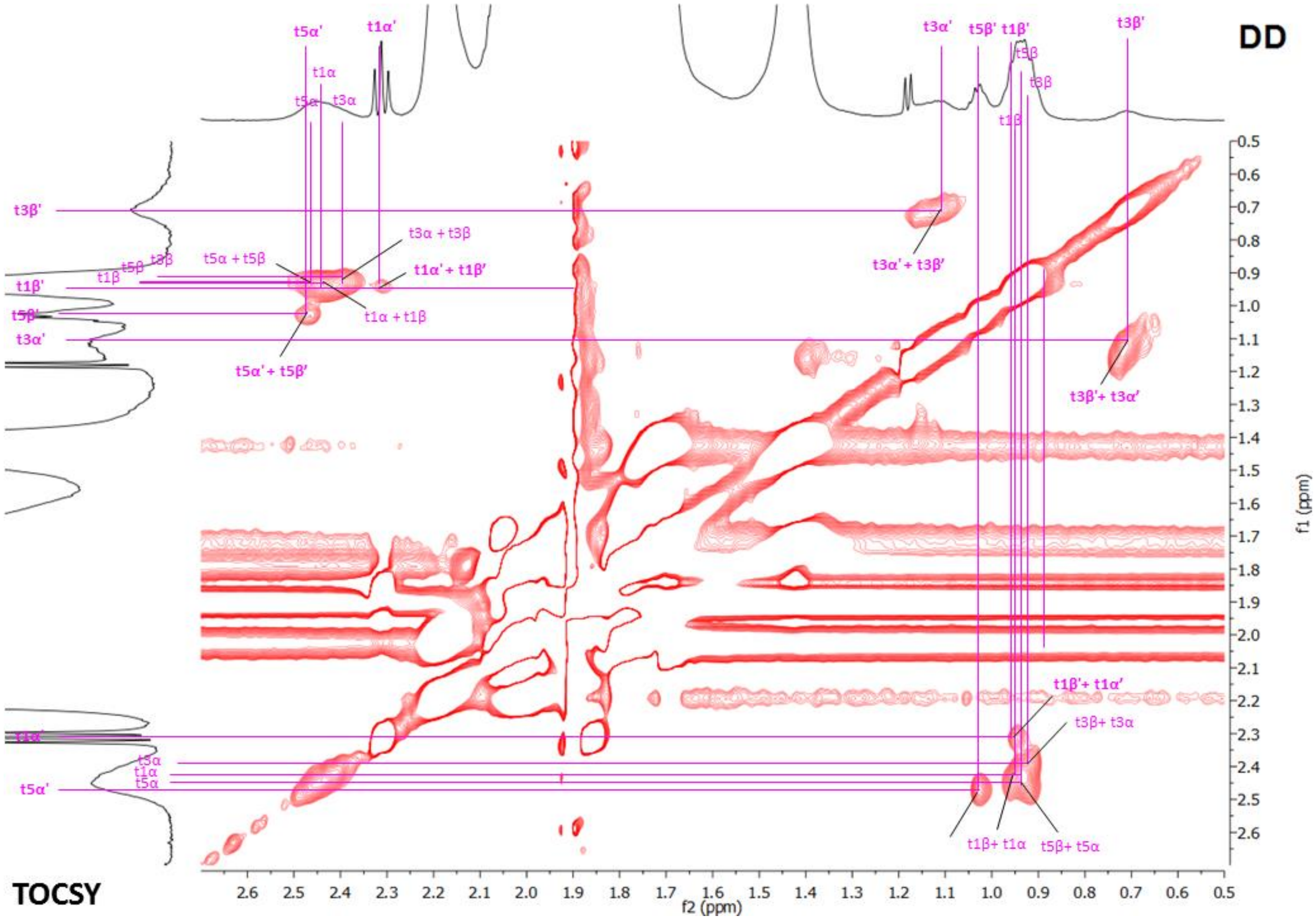

Figure S55. Partial {^{1}H-^{1}H}-TOCSY NMR spectrum of receptors (±)-**2** (0.21 mM each) and *N*-acetyl-D-glucosamine **8** (10.6 mM) and in D_2O including assignments. Note that chemical shifts are offset by about 0.1 p.p.m.[7]
